# Supplementary material for: Structure-function relationship of alpha-synuclein fibrillar polymorphs derived from distinct synucleinopathies
Source: Mol Syst Biol. 2026 Mar 11;22(6):868–901. doi: 10.1038/s44320-026-00199-5 (PMC13230553; doi:10.1038/s44320-026-00199-5)
Supplement: Supplementary file 2 — Appendix [file 44320_2026_199_MOESM2_ESM.pdf]

Appendix for:

Structure-function relationship of alpha-synuclein fibrillar polymorphs derived from distinct synucleinopathies

Table of Contents:

Appendix Figure S1 pages 3-4
Appendix Figure S2 page 5
Appendix Figure S3 page 6
Appendix Figure S4 page 7
Appendix Figure S5 page 8
Appendix Figure S6 page 9
Appendix Figure S7 page 10
Appendix Figure S8 page 11
Appendix Figure S9 page 12
Appendix Figure S10 page 13
Appendix Figure S11 page 14
Appendix Figure S12 page 15
Appendix Figure S13 page 16
Appendix Figure S14 page 17
Appendix Figure S15 page 18
Appendix Figure S16 page 19
Appendix Figure S17 page 20
Appendix Figure S18 page 21
Appendix Figure S19 page 22
Appendix Figure S20 page 23
Appendix Figure S21 page 24
Appendix Figure S22 page 25
Appendix Figure S23 page 26
Appendix Figure S24 page 27
Appendix Figure S25 page 28
Appendix Figure S26 page 29
Appendix Figure S27 page 30
Appendix Figure S28 page 31
Appendix Figure S29 page 32
Appendix Figure S30 page 33
Appendix Table S1 page 34
Appendix Table S2 page 35
Appendix Table S3 page 36
Appendix Table S4 page 37
Appendix Table S5 page 38
Appendix Table S6 page 39
Appendix Table S7 page 40

Appendix Table S8 page 41
Appendix Table S9 page 42
Appendix Table S10 page 43

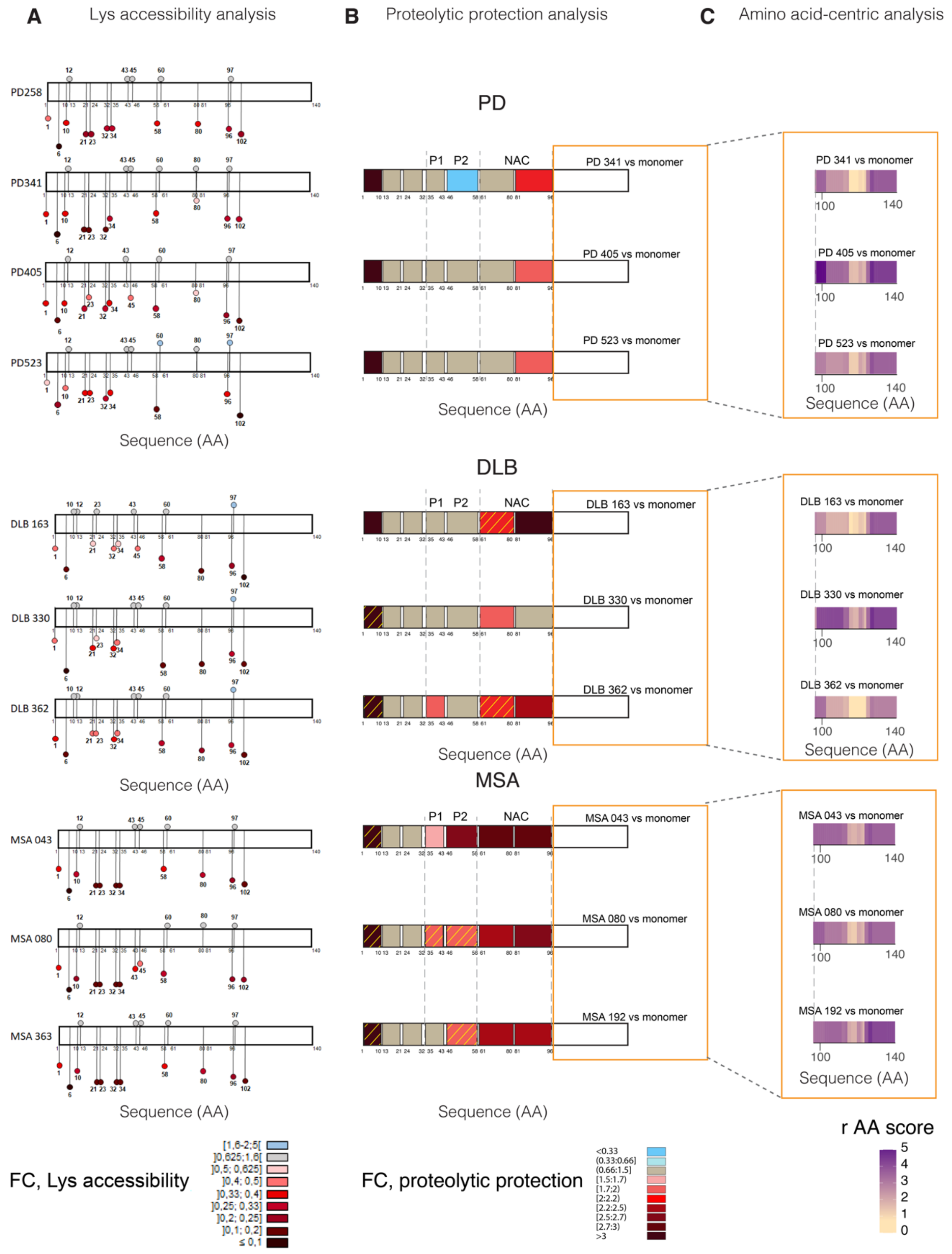

Appendix Figure S1. Disease-specific structural features of PMCA-amplified fibrils from individual patients. Lys accessibility (**A**), proteolytic protection (**B**) and amino acid-centric analysis of C-terminus proteolysis (**C**) are shown. (**A**) Covalent painting-based Lys accessibility analysis of PD, DLB and MSA derived αSyn fibrils for individual patients vs αSyn monomer. The color scale shows fold change of lysine accessibility vs monomer at all lysines within αSyn primary structure; darker hues indicate decreased accessibility (n=4 patients for PD, and n=3

patients each for DLB and MSA). (**B**) LiP-MS-based proteolytic protection analysis for PD, DLB, and MSA fibrils vs αSyn monomer for individual patients. The color scale shows fold change of proteolytic protection vs monomer along αSyn primary structure; darker hues show increased protection (n=3 patients per disease, n=4 technical replicates per sample). The hatched area indicates the regions exhibiting FC>1.5 that did not pass the significance cut-off (p-val<0.05), and corresponding statistically significant changes were recorded for the same region for at least one other patient. (**C**) LiP-MS-based amino acid-centric analysis of proteolytic patterns in αSyn C-terminal moiety of PD, DLB and MSA fibrils for individual patients vs αSyn monomer. The color scale shows the r score, a measure of the change in protease accessibility per amino acid, plotted along the αSyn C terminal primary structure. Non-significantly different regions are colored in beige. Significantly changing regions are colored in violet. Darker violet color indicates stronger difference. N=3 patients per disease, n=4 technical replicates per sample.

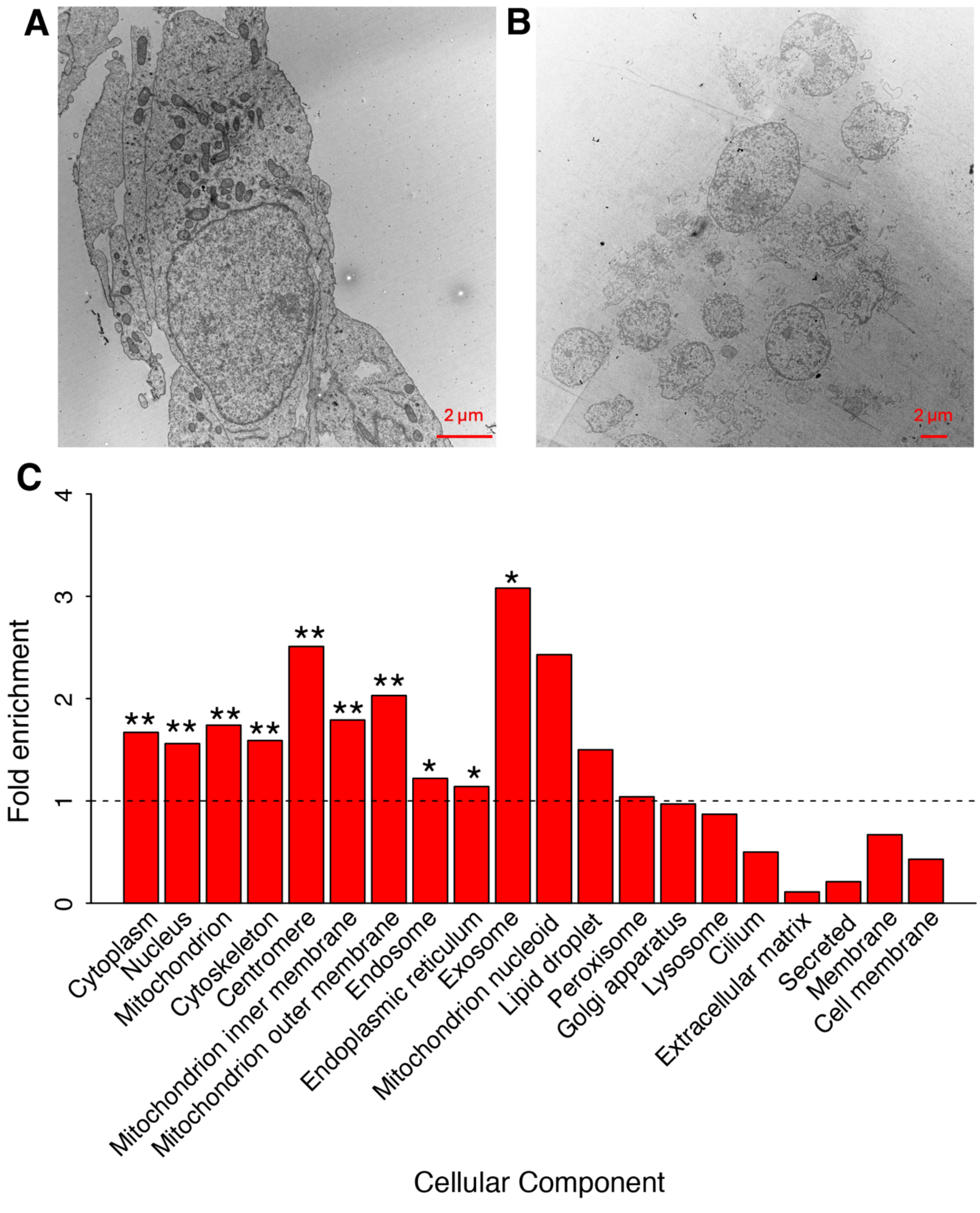

Appendix Figure S2. Evaluation of cell lysis efficiency. (**A**, **B**) Electron microscopy of intact (A) and lysed (B) SH-SY5Y cells. (**C**) Enrichment analysis (Uniport Keywords cellular component) of proteins represented by semi-tryptic peptides (i.e., those produced by PK cleavage and detected in SH-SY5Y lysate). Stars indicate statistical significance (*q-val <0.05, **q-val<0.01)

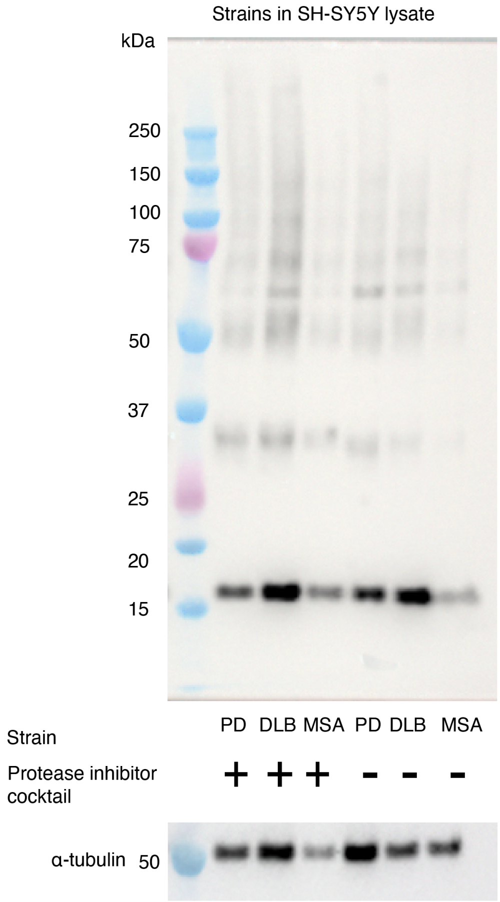

Appendix Figure S3. Effect of protease inhibitors on αSyn cleavage in SH-SY5Y lysate. Western blot against αSyn (stained with the 5G4 anti-αSyn antibody, epitope aa 46-53) after incubation of the same amount of αSyn fibrillar strains (1.5 µg) in the presence or absence of a cOmplete protease inhibitors cocktail (Roche) for 15 min in cell lysate. α-tubulin served as a loading control. The same blot is shown in Figure 2D, cropped to highlight a subset of experimental conditions.

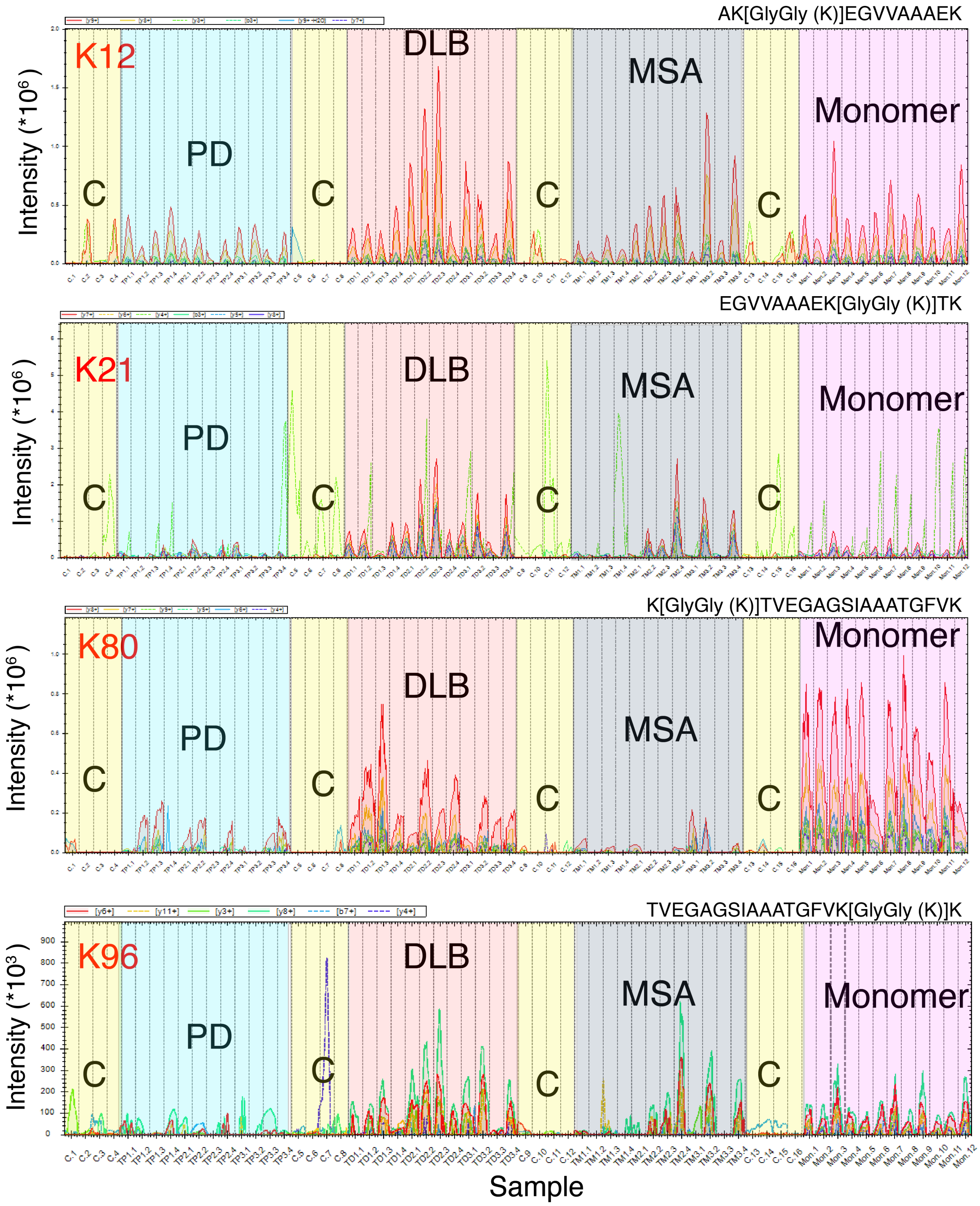

Appendix Figure S4. Quality control of the identification of ubiquitinated peptides. Extracted ion chromatograms of fragment ions corresponding to identified ubiquitinated peptides of αSyn upon spiking into SH-SY5Y lysate for 15 min. Different colors represent different fragments. Dashed peaks correspond to contaminants. Conditions are labeled as follows: PD, DLB, MSA, Monomer for spiked αSyn, C- control SH-SY5Y lysate. Normalization on protein abundance was performed at the later data analysis stage.

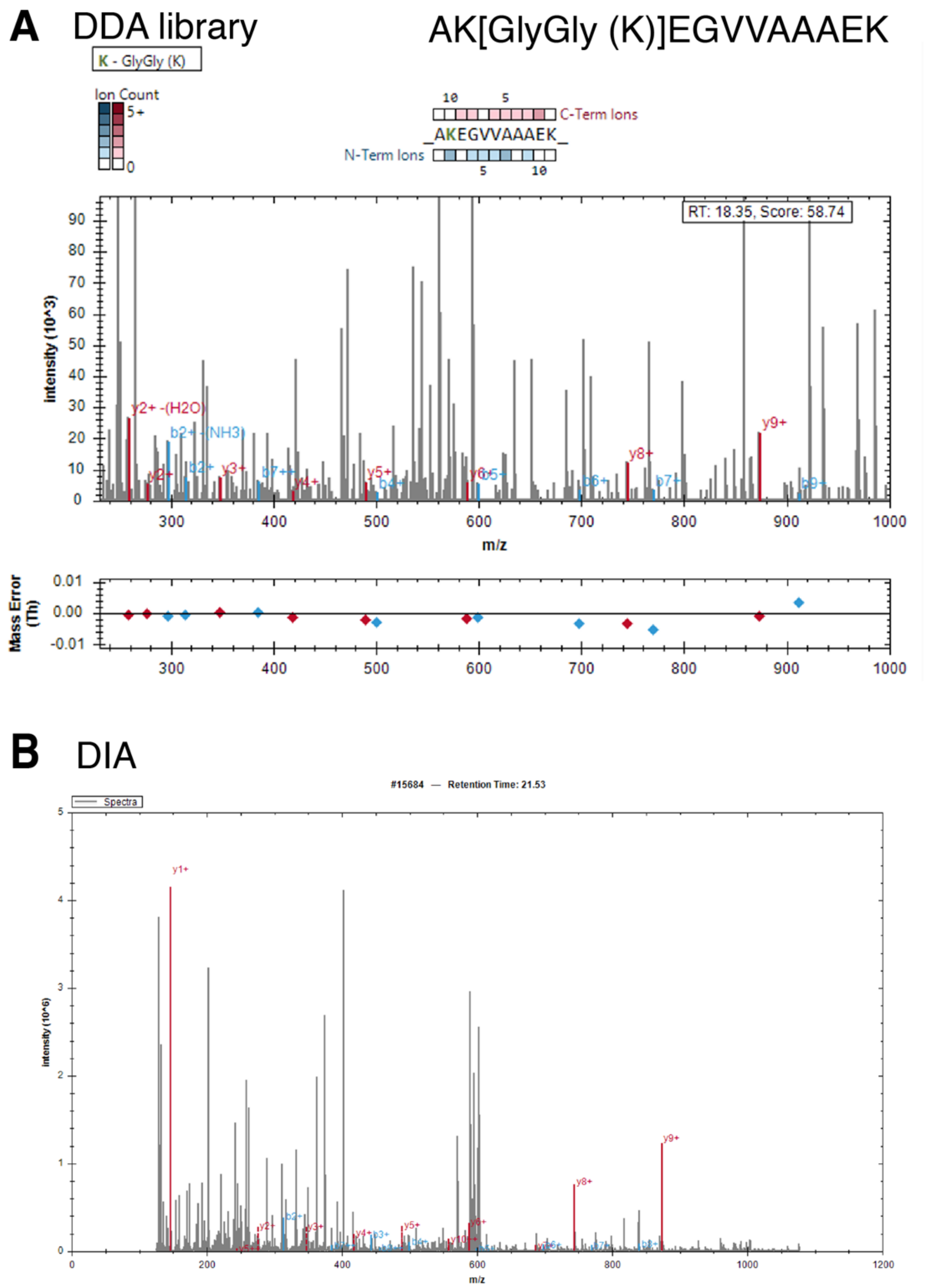

Appendix Figure S5. Quality control of the identification of the peptide AK[GlyGly(K)]EGVVAAAEK. MS2 spectra corresponding to identified ubiquitinated peptides of αSyn upon spiking into SH-SY5Y lysate acquired in (**A**) DDA (for library) and (**B**) DIA mode.

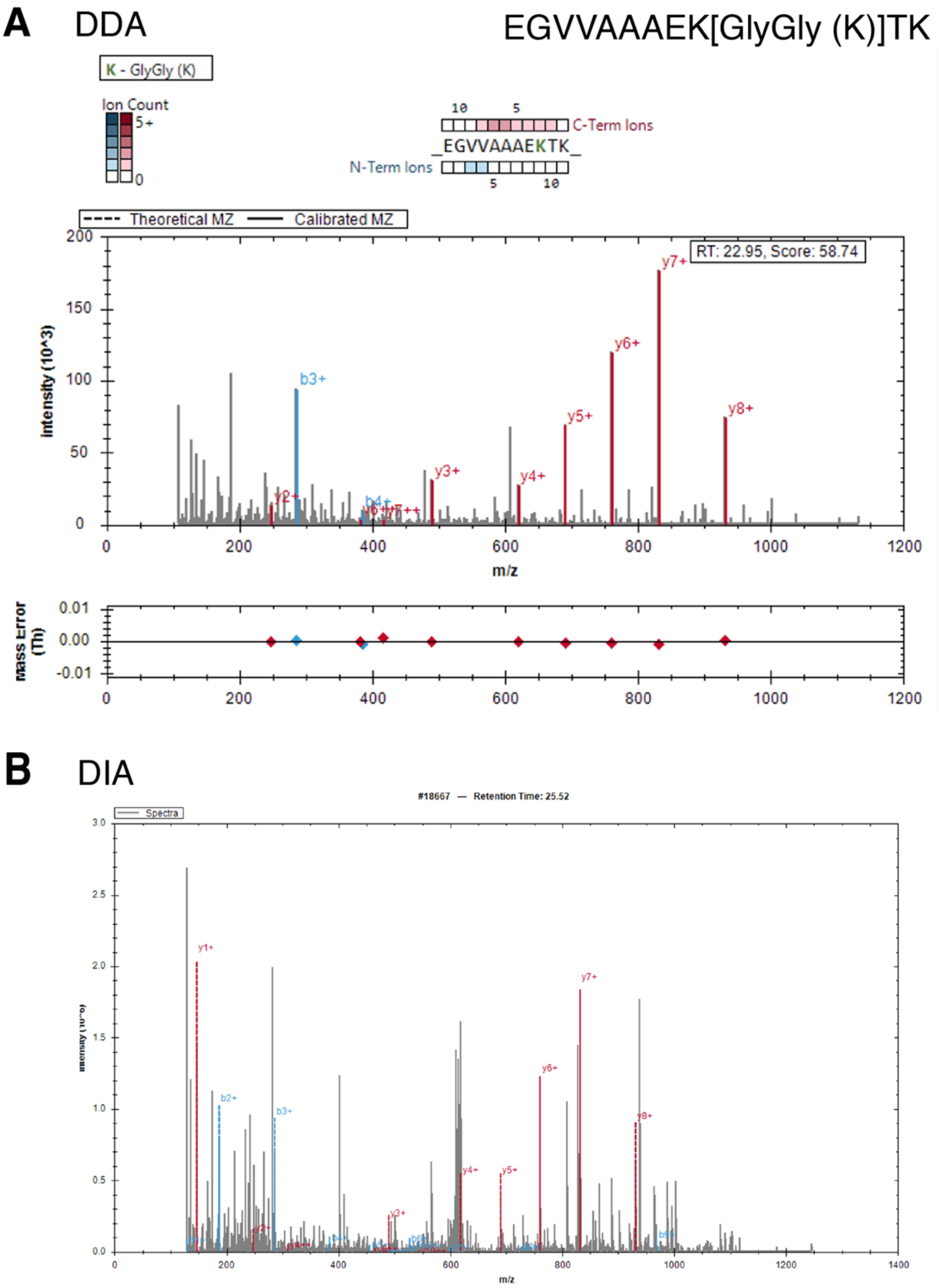

Appendix Figure S6. Quality control of the identification of the peptide EGVVAAAEK[GlyGly(K)]TK. MS2 spectra corresponding to identified ubiquitinated peptides of αSyn upon spiking into SH-SY5Y lysate acquired in (**A**) DDA (for library) and (**B**) DIA mode.

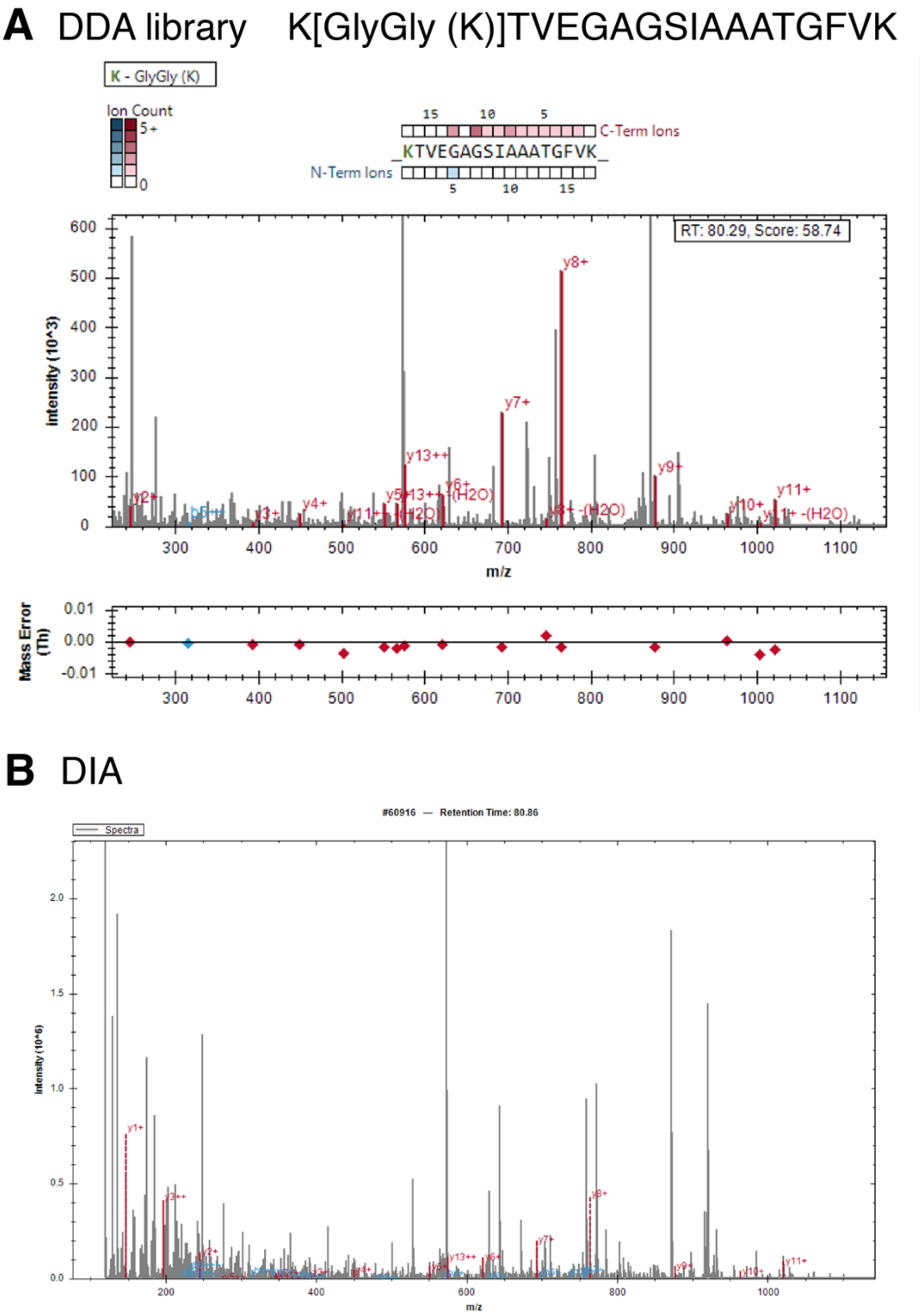

Appendix Figure S7. Quality control of the identification of the peptide K[GlyGly(K)]TVEGAGSIAAATGFVK. MS2 spectra corresponding to identified ubiquitinated peptides of αSyn upon spiking into SH-SY5Y lysate acquired in (**A**) DDA (for library) and (**B**) DIA mode.

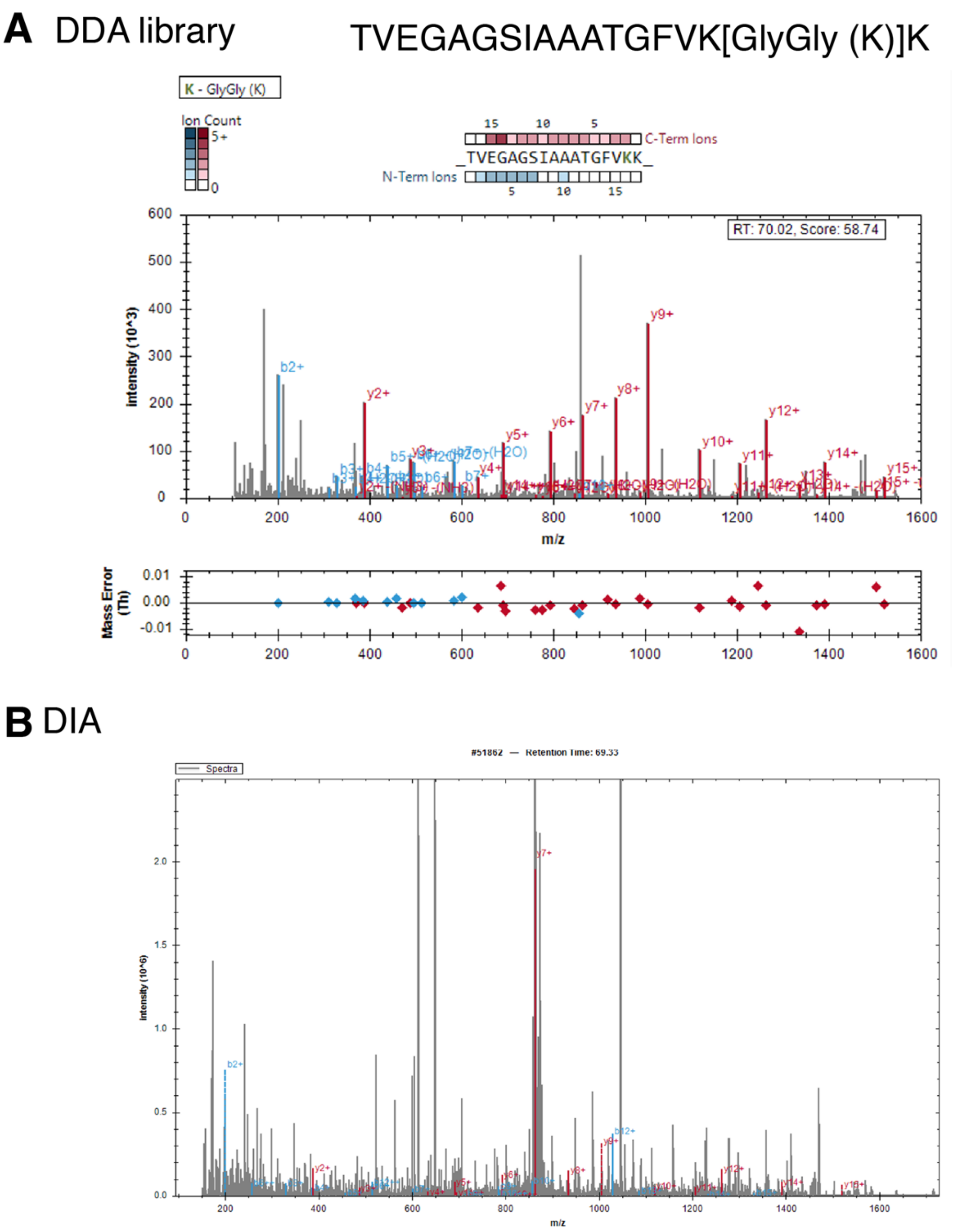

Appendix Figure S8. Quality control of the identification of the peptide TVEGAGSIAAATGFVK[GlyGly(K)]K. MS2 spectra corresponding to identified ubiquitinated peptides of αSyn upon spiking into SH-SY5Y lysate acquired in (**A**) DDA (for library) and (**B**) DIA mode.

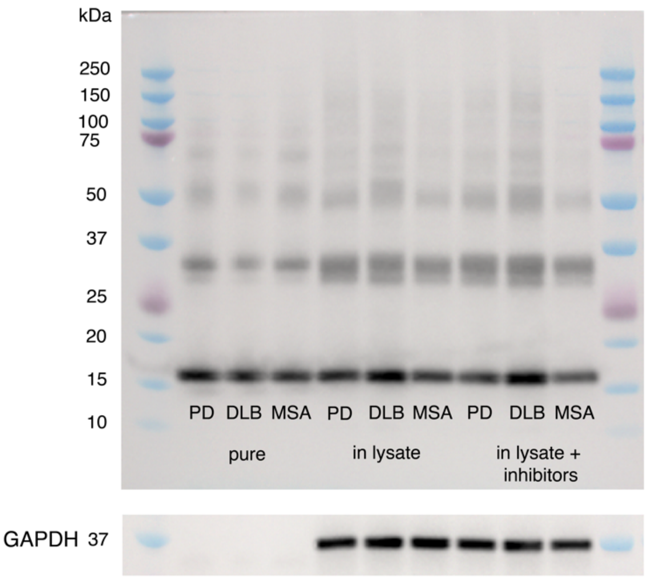

Appendix Figure S9. Western blot analysis of αSyn fibrillar polymorphs in pure form and after incubation in cell lysate in the absence or presence of proteases inhibitors. The Western blot against αSyn (stained with 5G4 anti-αSyn antibodies) for amplified pure PD, DLB and MSA strains (first three lanes). Western blot against αSyn after incubation of the same amount of αSyn (1.5 µg) for 15 min in SH-SY5Y cell lysate in the absence or the presence of a cOmplete protease inhibitors cocktail (Roche). GAPDH was used as a loading control (anti-GAPDH antibody GA1R). The samples were incubated for 60h in 8M urea to disassemble the fibrils.

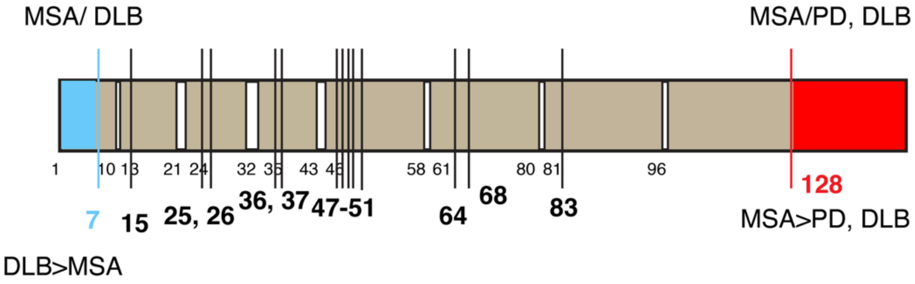

Appendix Figure S10. Mapping cell lysate endogenous protease cleavages on the sequence of αSyn. Vertical lines show endogenous cleavage sites, detected based on semi-tryptic peptides found in tryptic control data (i.e., where no PK is added). Cleavage products that are significantly different between strains are labeled in blue and red.

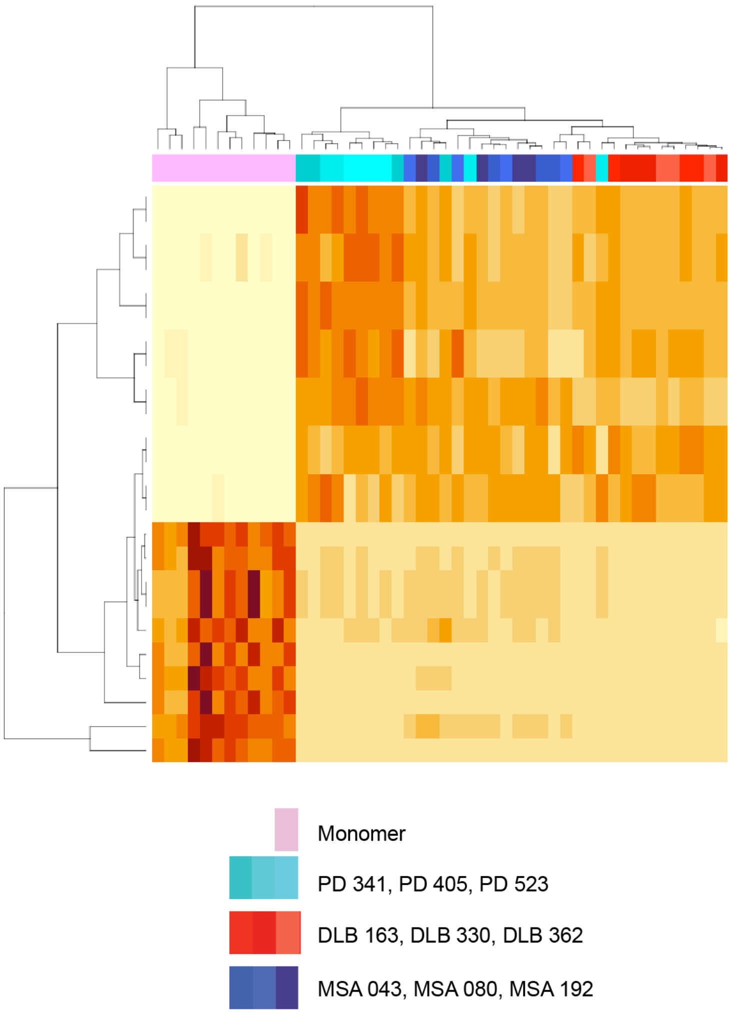

Appendix Figure S11. **Conformation-specific peptide patterns of αSyn species.** Clustering analysis of all peptides for monomeric αSyn (pink) and PD (cyan), DLB (red) and MSA (dark blue) fibrils. Individual patients are shown with different shades of each colour. N=3 patients per disease, n=4 technical replicates per sample.

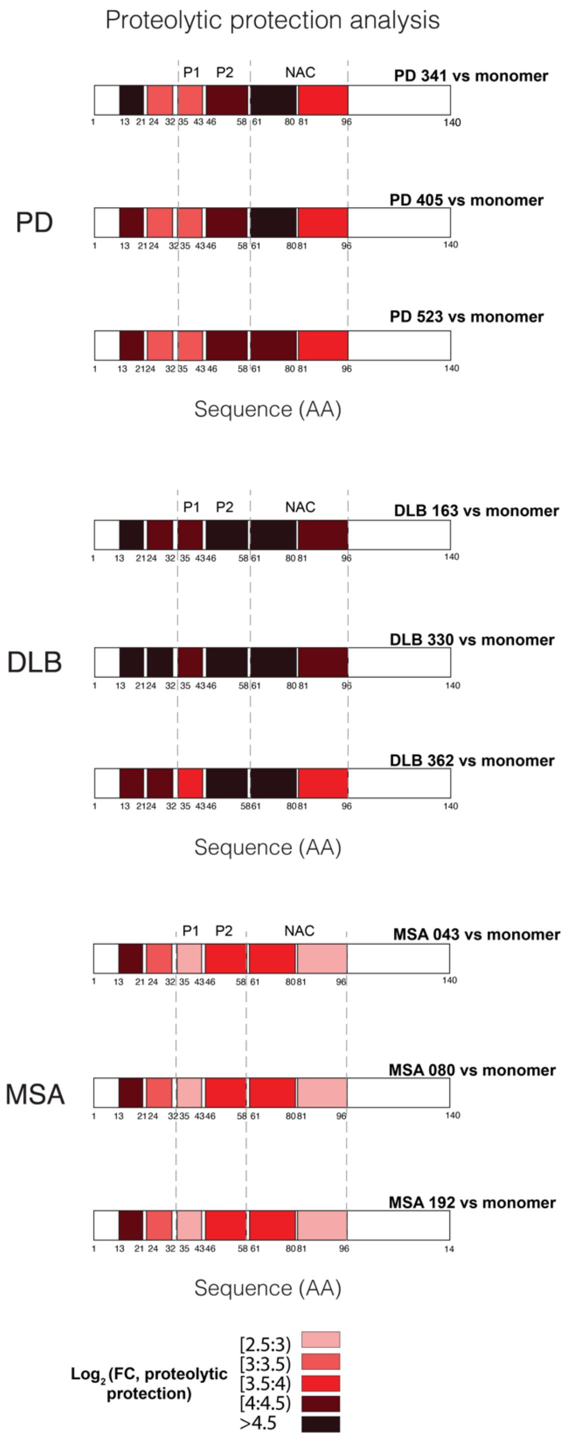

Appendix Figure S12. Disease-specific structural features of PMCA-amplified fibrils from individual patients *in situ* (lysate). LiP-MS-based proteolytic protection analysis for PD, DLB, and MSA fibrils vs αSyn monomer for individual patients. The colour scale shows fold change of proteolytic protection vs monomer along the αSyn primary structure; darker hues show increased protection (n=3 patients per disease, n=4 technical replicates per sample).

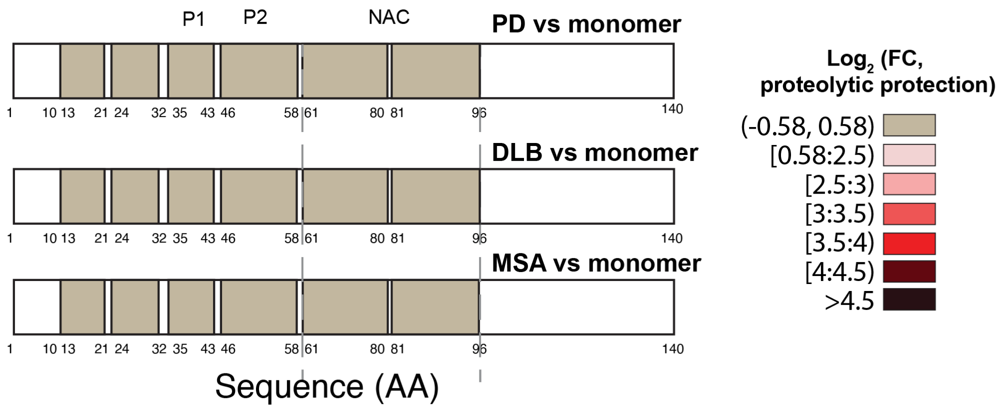

Appendix Figure S13. Tryptic control data shows no protection in fully tryptic peptides of αSyn. Grey bars correspond to non-significantly different peptides between the fibrillar polymorphs and the monomer in trypsin only digestion condition. This data supports that additional protection of fully peptides in LiP condition is due to difference in structure.

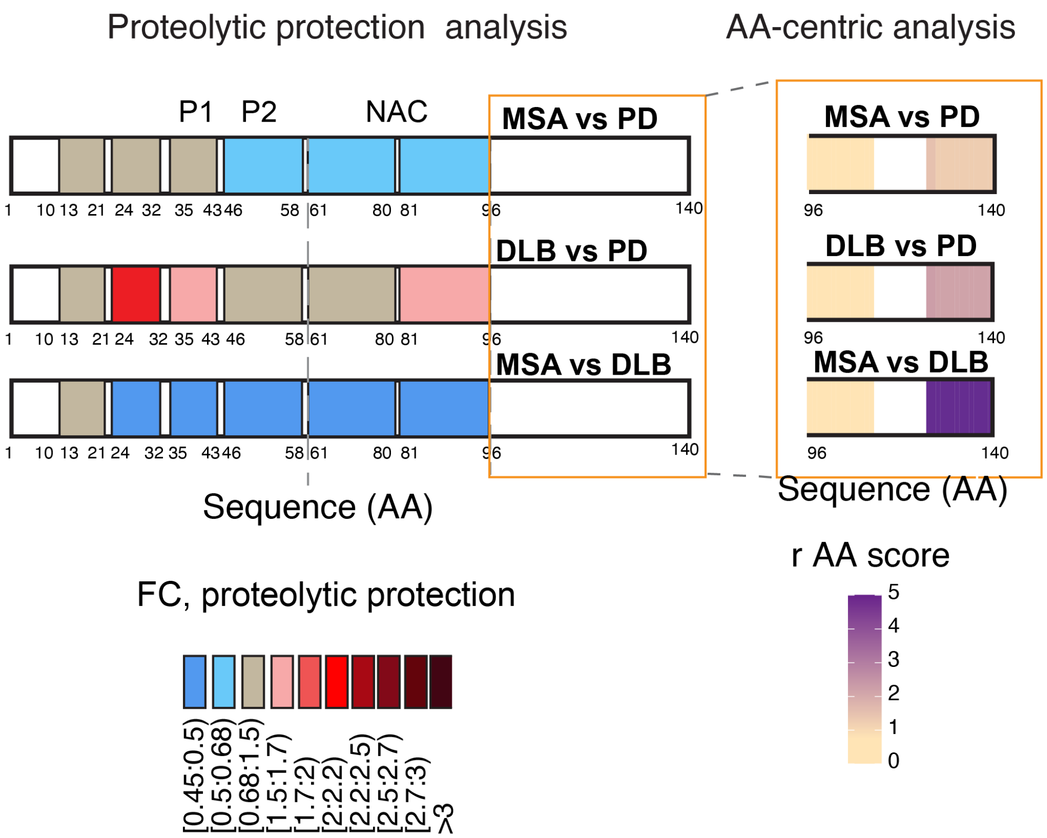

Appendix Figure S14. Direct structural comparison of PD, DLB and MSA strains in SH-SY5Y cellular lysate. LiP-MS-based proteolytic protection analysis for PD, DLB, and MSA derived αSyn fibrils vs each other in cellular lysate (left). The color scale shows the fold change of proteolytic protection vs each other along the αSyn sequence; darker red hues show increased protection, and darker blue hues indicate decreased protection (n=3 patients per disease, n=4 technical replicates per sample). LiP-MS-based amino acid-centric analysis of proteolytic patterns in αSyn C-terminal moiety of PD, DLB, and MSA derived αSyn fibrils vs each other (right). The color scale shows the r score, a measure of the change in protease susceptibility per amino acid, plotted along the αSyn C-terminal primary structure (n=3 patients per disease, n=4 technical replicates per sample).

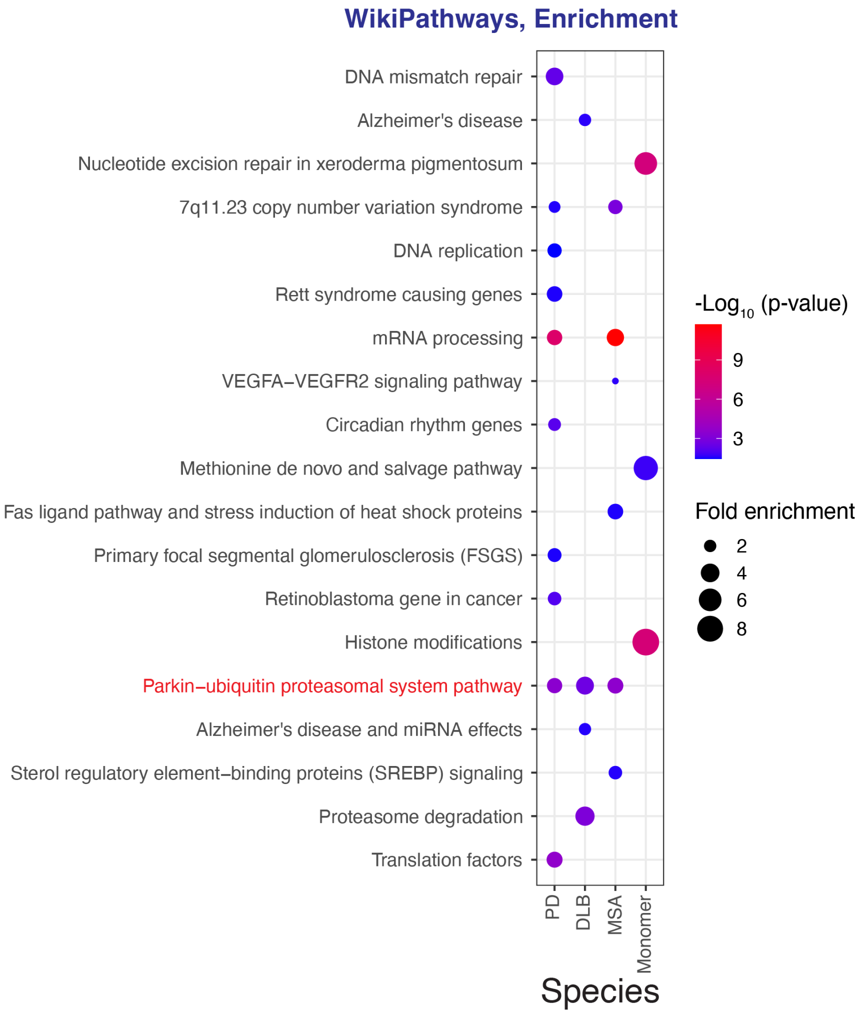

Appendix Figure S15. Pathways enrichment analysis for interactors of the different αSyn species. Functional enrichment analysis (WikiPathways) for the set of proteins that show structural changes upon spiking of the different αSyn species into SH-SY5Y lysate. All significant enrichments are shown (p-val<0.05).

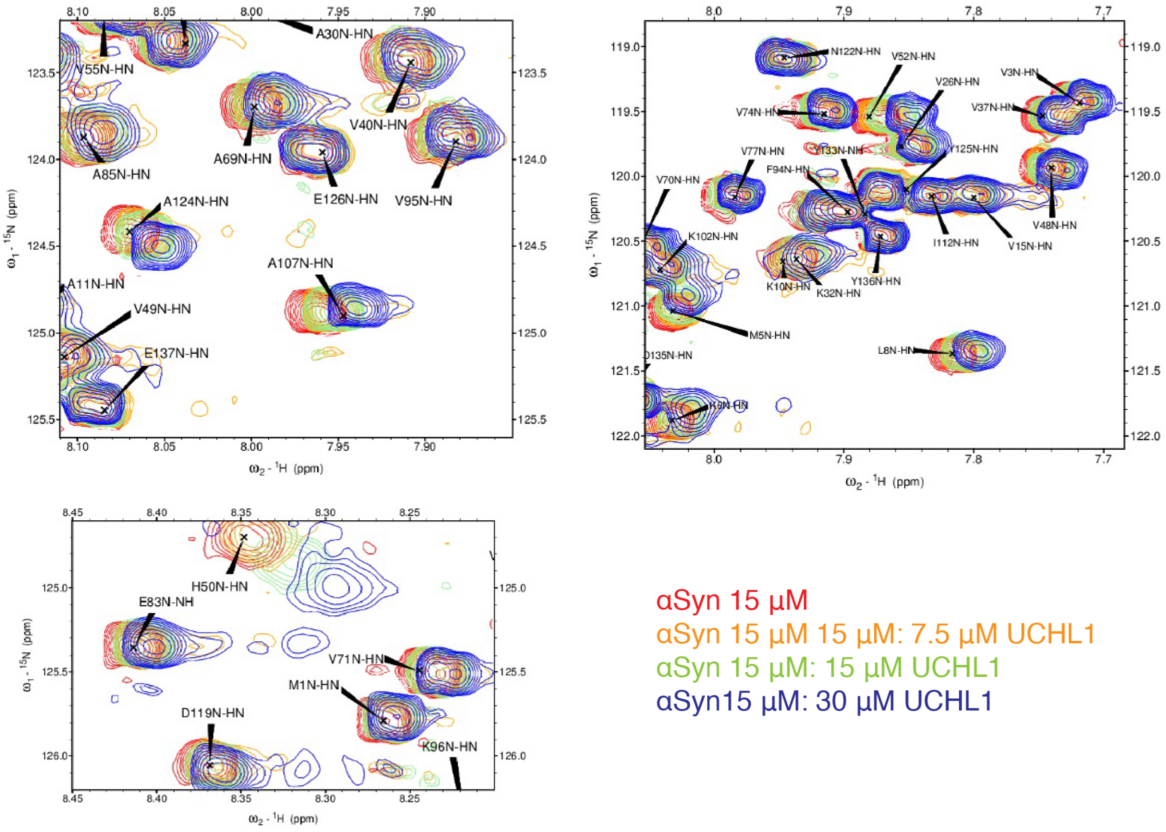

Appendix Figure S16. NMR validation of interaction between UCHL1 and αSyn monomer. 2D [^{15}N,^{1}H] HMQC NMR spectra of 15 µM ^{15}N labelled αSyn in the absence (red) or presence of increasing quantities of purified UCHL1 (yellow, green, and magenta) in PBS. Black arrowheads are used to label the peaks.

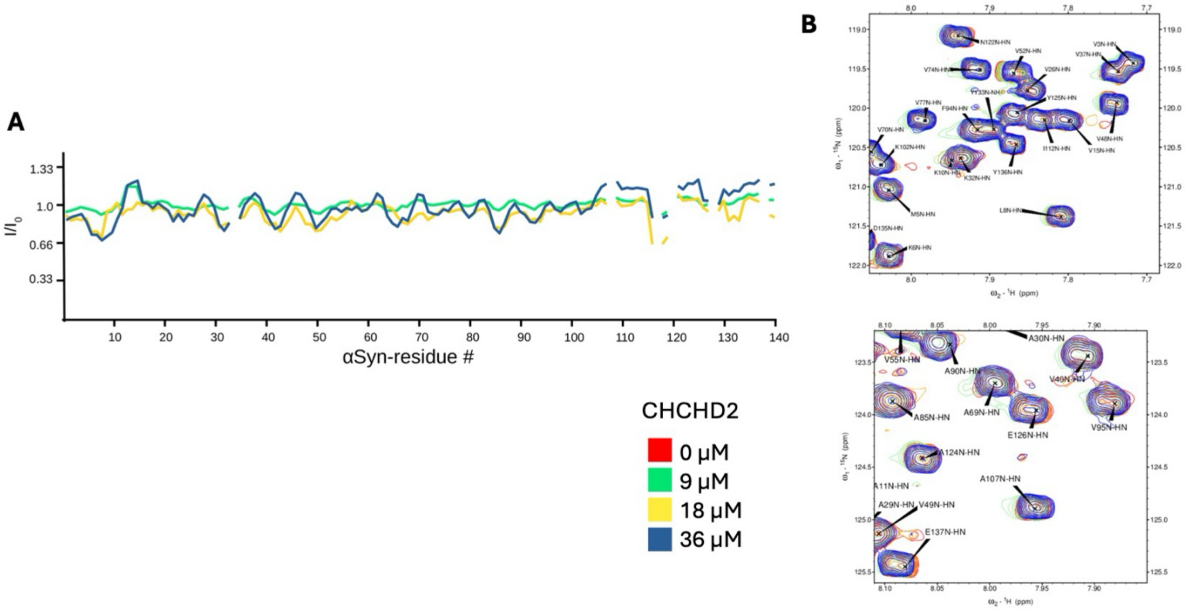

Appendix Figure S17. Control of αSyn interaction specificity measured by NMR. No direct interaction between αSyn with CHCHD2 measured on ^{15}N-labeled αSyn. 2D [^{15}N,^{1}H] HMQC NMR of 18µM wild-type αSyn in PBS at pH 7.4 with varying concentrations of human CHCHD2. (**A**) Peak intensity ratios of wild-type αSyn. (**B**) Chemical shift perturbations (CSPs) of wild-type αSyn with subsections of the HMQC spectrum with peak labels (lower panel).

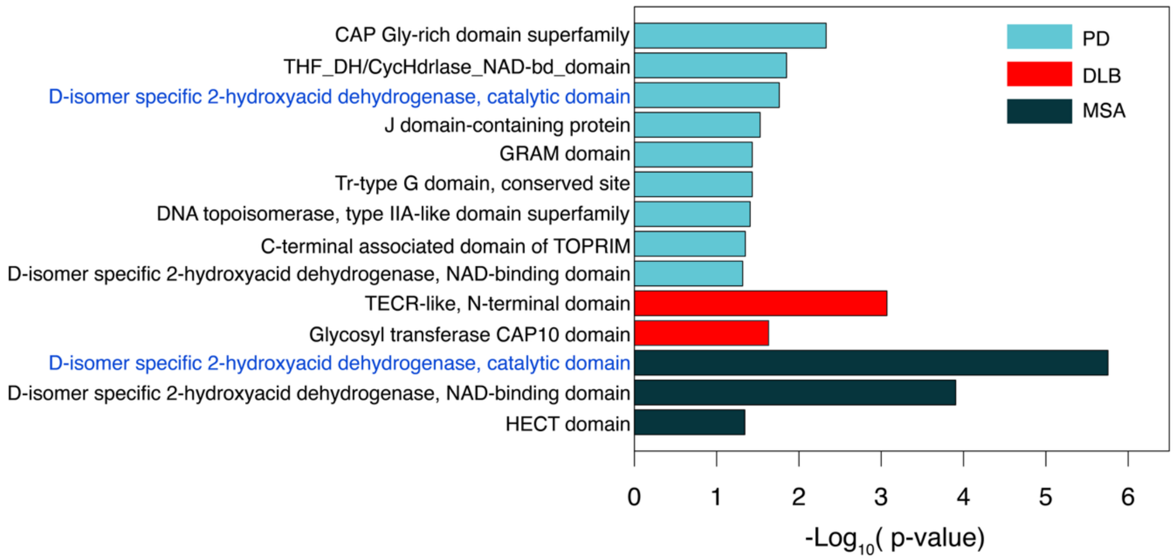

Appendix Figure S18. Enrichment analysis of the protein domains for interactomes of αSyn disease-specific strains revealed by LiP-MS. The domains enriched for multiple αSyn fibrillar polymorphs are shown in blue.

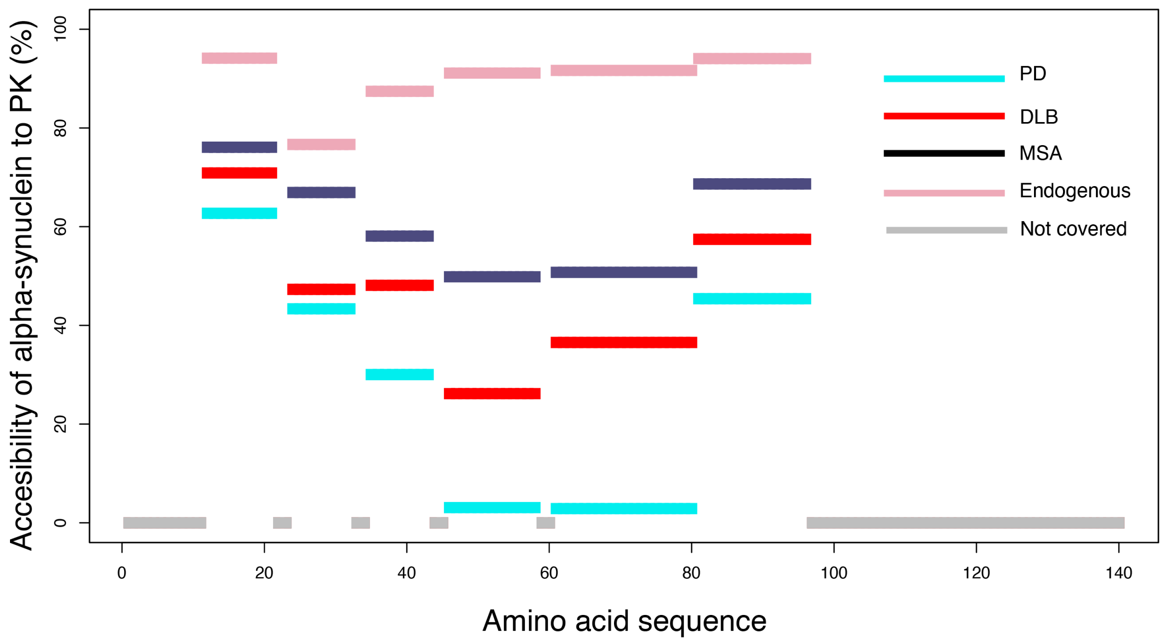

Appendix Figure S19. Accessibility of αSyn to PK in SH-SY5Y cell seeding model. Percentage of the loss of intensity of fully tryptic peptides under PK digestion of αSyn in the cell seeding model (accessibility to PK). The accessibility of endogenous αSyn (without seeding) in SH-SY5Y cells is given in pink. Peptides are mapped along αSyn sequence. The αSyn sequence regions that are not covered by fully tryptic peptides are shown in grey.

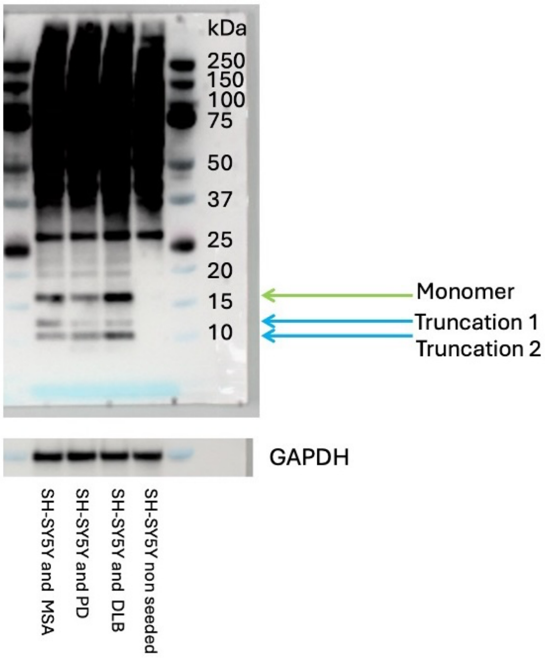

Appendix Figure S20. Two truncated species of αSyn revealed after strains uptake by live SH-SY5Y cells. Western blot analysis of αSyn strains using a mixture of two antibodies (5G4 (aa 47-52) and 42/α-Synuclein (aa 91-99)) in lysates of living SH-SY5Y cells that were incubated with 250 nM of each αSyn strain for 24h. Green arrow indicates the monomer. Cyan arrows indicate truncated forms. SDS-PAGE was done using MES buffer. The higher molecular weight signal is primarily due to antibody nonspecific binding in the SH-SY5Y lysate. GAPDH served as a loading control using GA1R anti-GAPDH antibody on the same blot. The same blot is shown in Figure 4C, cropped to highlight the lower molecular weight range.

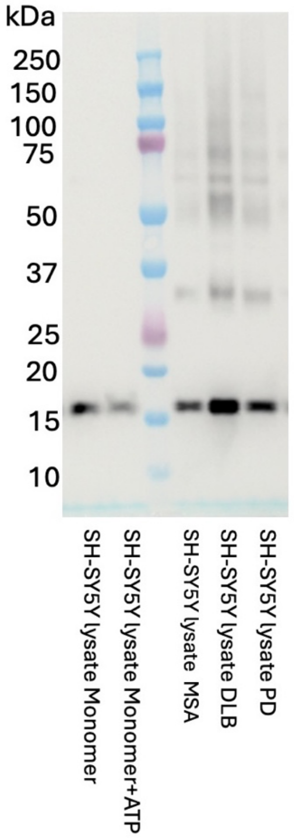

Appendix Figure S21. Western blot analysis of αSyn monomer and depolymerized αSyn fibril strains spiked into SH-SY5Y lysate using a mixture of two antibodies 5G4 (epitope aa 47-52) and 42/α-Synuclein (aa 91-99) antibody.

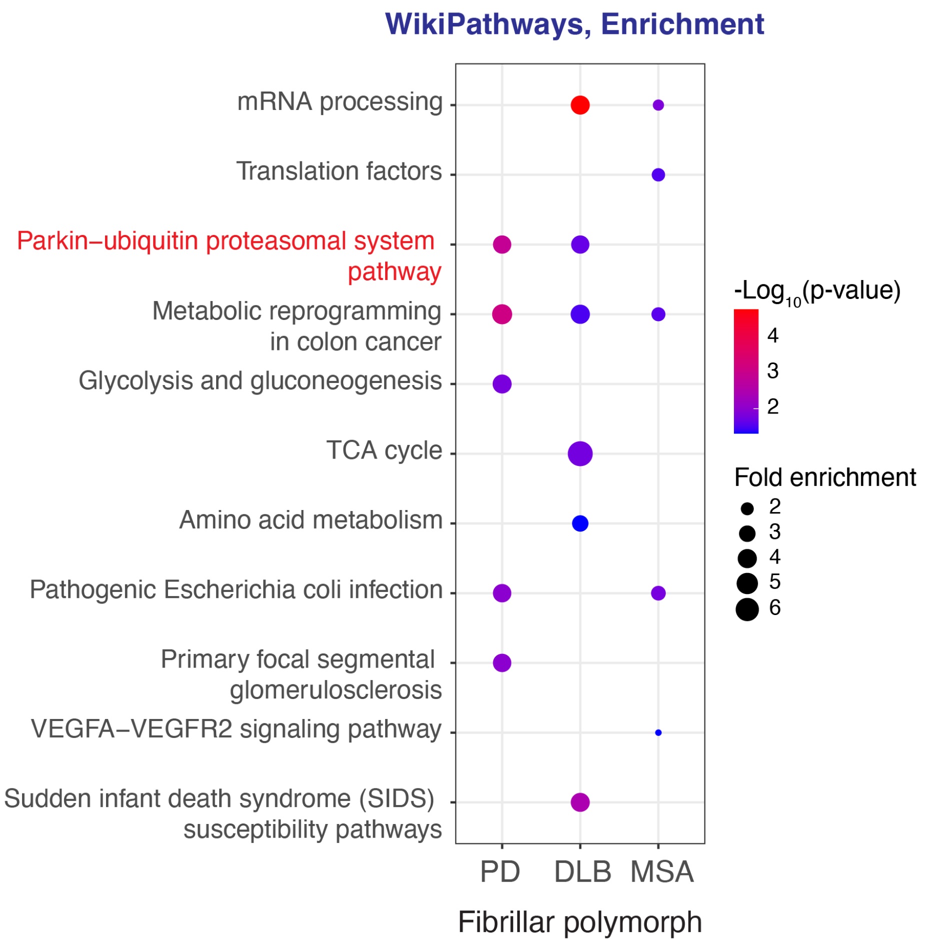

Appendix Figure S22. Pathway enrichment analysis for the set of proteins that structurally respond to the different αSyn fibrillar polymorphs in living SH-SY5Y cells. Functional enrichment analysis (WikiPathways) for the set of proteins that show structural changes upon cell infection with the different αSyn fibrillar polymorphs. All significant enrichments are shown (p-val<0.05).

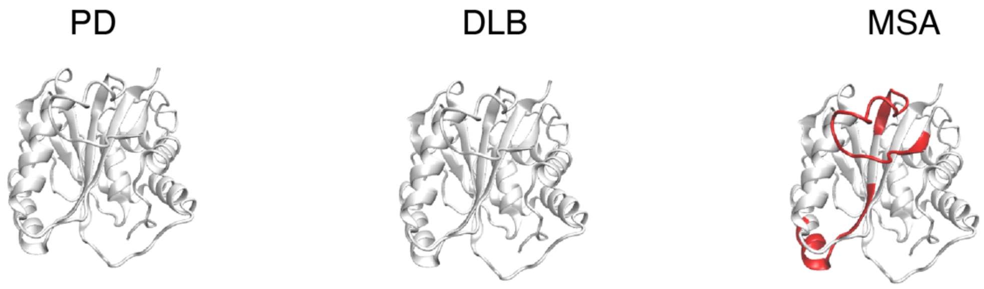

Appendix Figure S23. UCHL1 response to the uptake of αSyn PD, DLB, and MSA fibrillar polymorphs in SH-SY5Y. Structural models show LiP-MS hit peptides mapped onto UCHL1 (PDBID 2etl) structure that change upon uptake of the different αSyn fibrillar polymorphs (red: FC>2, q-val<0.05).

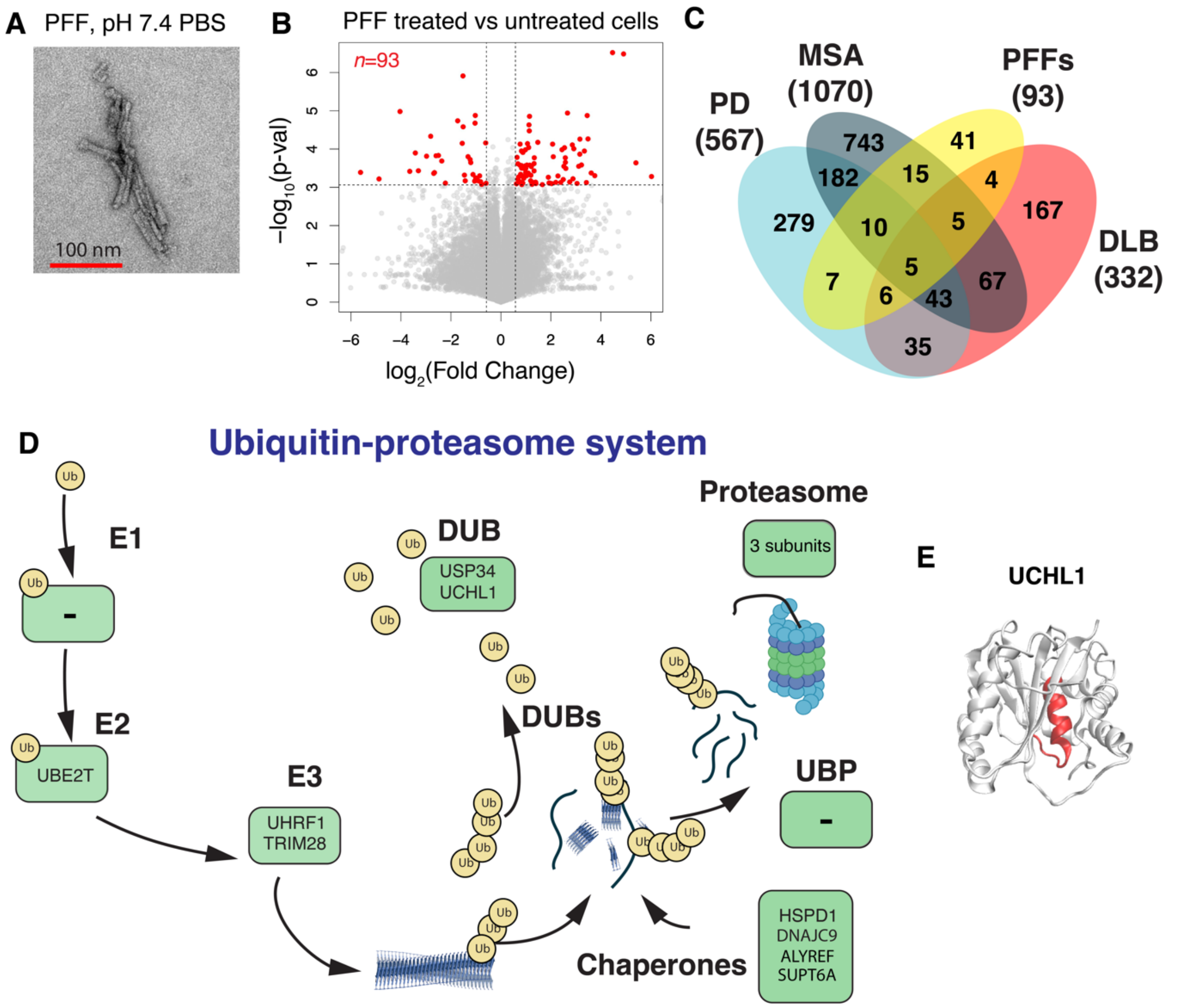

Appendix Figure S24. SH-SY5Y cells differentially respond to αSyn PFFs, PD, DLB, and MSA polymorphs. (**A**) TEM image of fragmented PFFs assembled at pH 7.4 in PBS. (**B**) Volcano plot highlights peptides for which susceptibility to PK changes significantly upon treatment with PFFs. (**C**) The Venn diagram shows the overlap of proteins undergoing protease susceptibility changes upon cell infection with the different disease-derived αSyn fibrillar polymorphs and PFFs (FC>1.5 q-val<0.05). (**D**) The diagram shows proteins annotated to the ubiquitin-proteasomal pathway where at least one peptide is subject to changes upon uptake of αSyn PFFs. (**E**) Structural model shows LiP-MS hit peptide mapped onto UCHL1 (PDBID 2etl) structure that change upon uptake of the αSyn PFFs (red: FC>2, q-val<0.05).

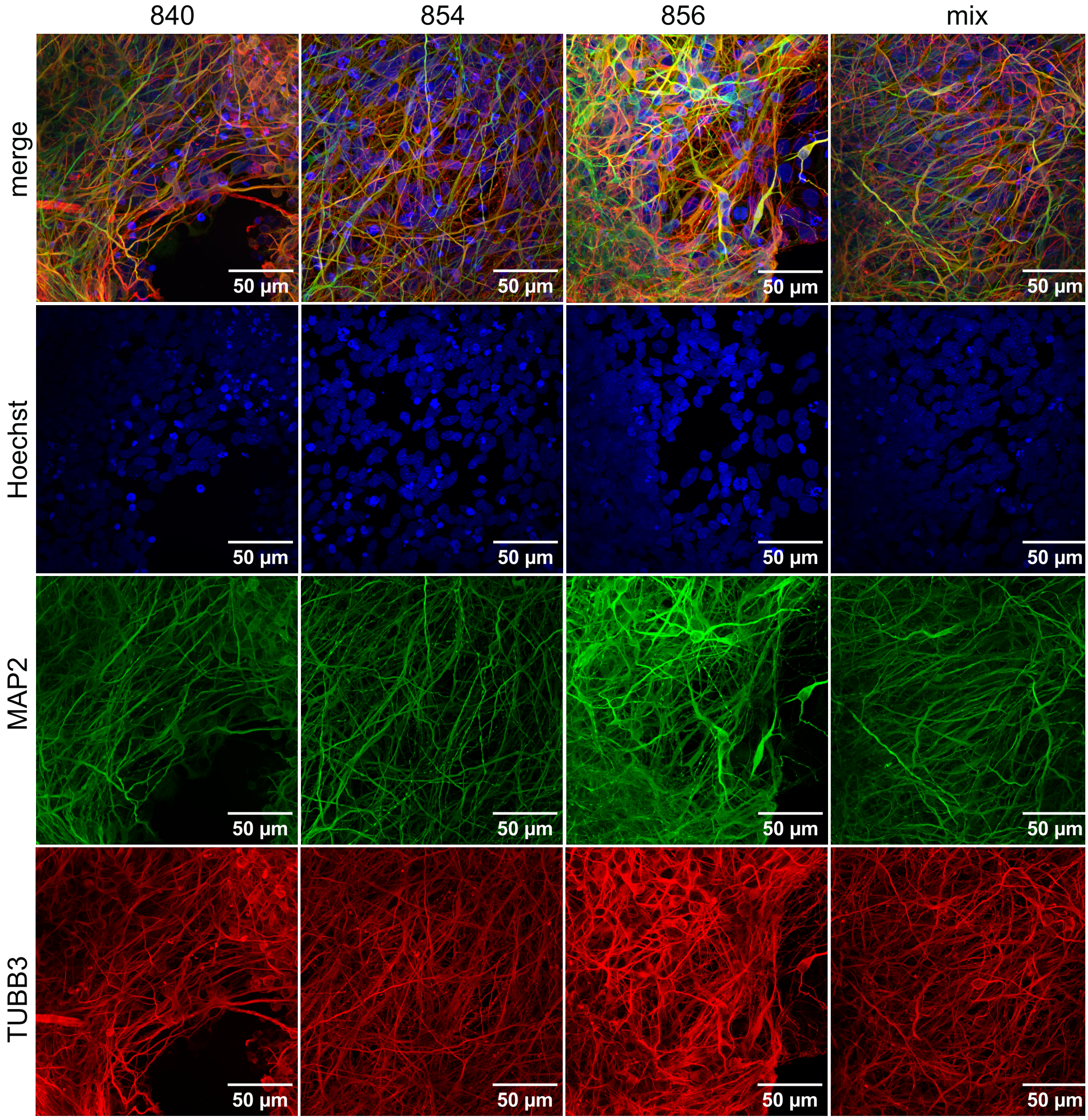

Appendix Figure S25. Fluorescence microscopy analysis of iPSCs-derived neurons. A mix of neurons derived from three patients (840, 854, and 856) was differentiated for 56 days and immunostained with Hoechst, anti-MAP2 (Millipore, MAB378, 1:300) and anti-beta 3 tubulin (TUBB3, BioLegend, 801202, 1:300) antibodies.

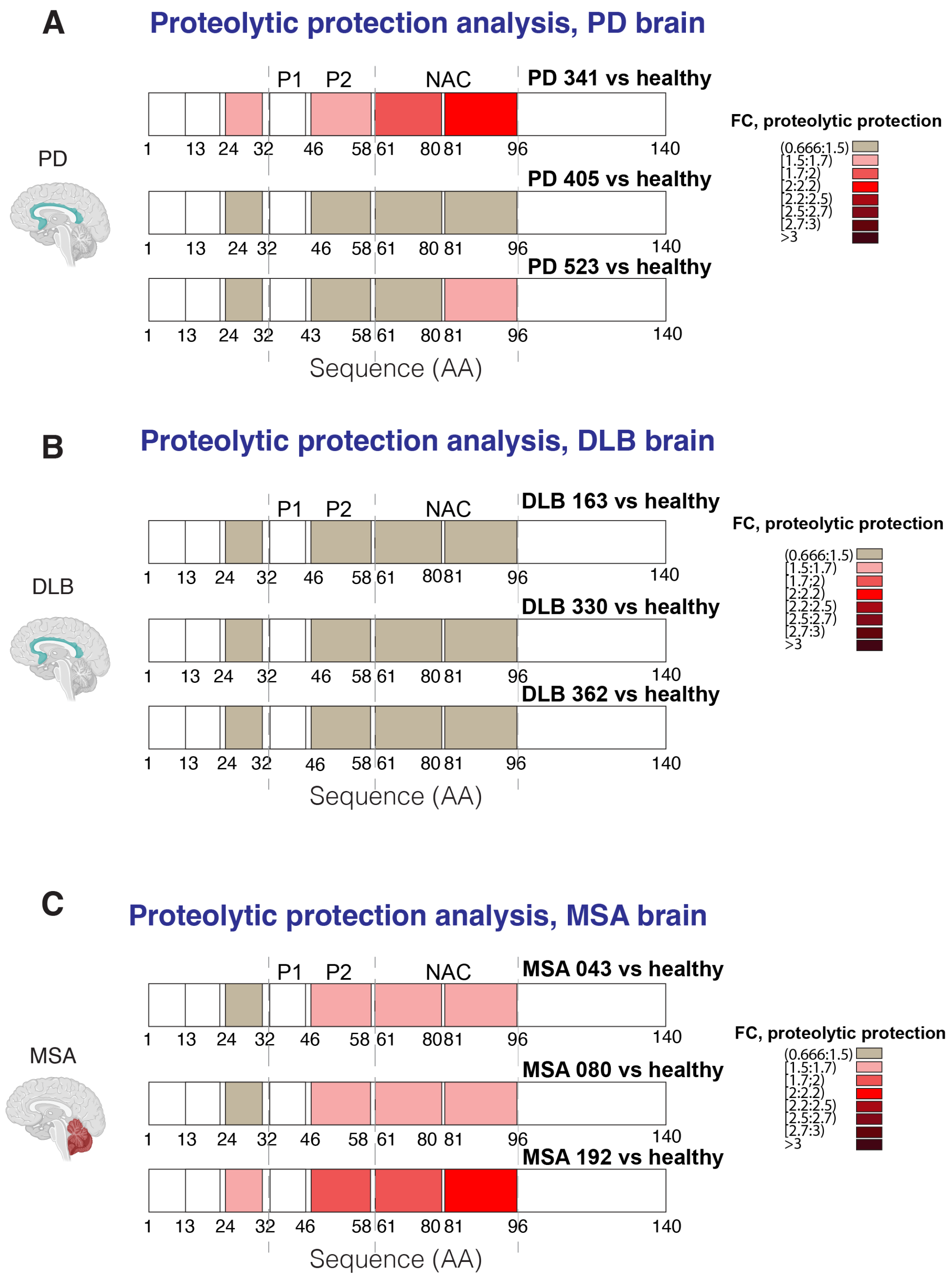

Appendix Figure S26. Disease-specific structures of αSyn in brain lysates from individual patients. LiP-MS-based proteolytic protection analysis of αSyn in PD, DLB, and MSA brain homogenates vs αSyn in healthy group, shown for individual patients. The colour scale shows fold change of proteolytic protection along the αSyn sequence; darker hues show increased protection (n=3 patients per disease and control, n=4 technical replicates per sample). We note that, for patient PD405, peptide 81-96 shows a fold change (1.42) just below that used for our significance cutoff (1.5).

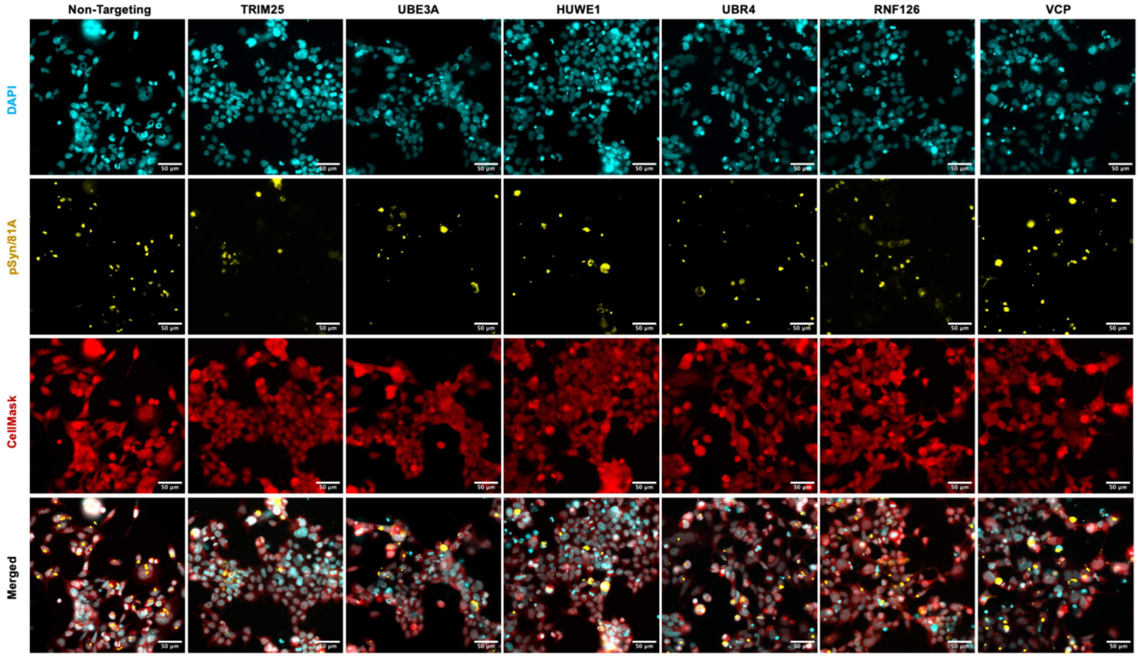

Appendix Figure S27. Functional testing of the effect of LiP-MS hits on αSyn accumulation upon uptake of PD fibrils. The images show cells in which the indicated putative αSyn regulatory factor was genetically upregulated using CRISPR, followed by incubation for 72h with αSyn PD fibrils. The fluorescence stains show pSer129 αSyn inclusions (pSer129/81A yellow), nuclei (DAPI, blue), and cytoplasm and nucleus (whole cell stain, red). NTG (Non-targeting control) indicates plasmids with a scrambled gRNA sequence. Scale bar 50 µm.

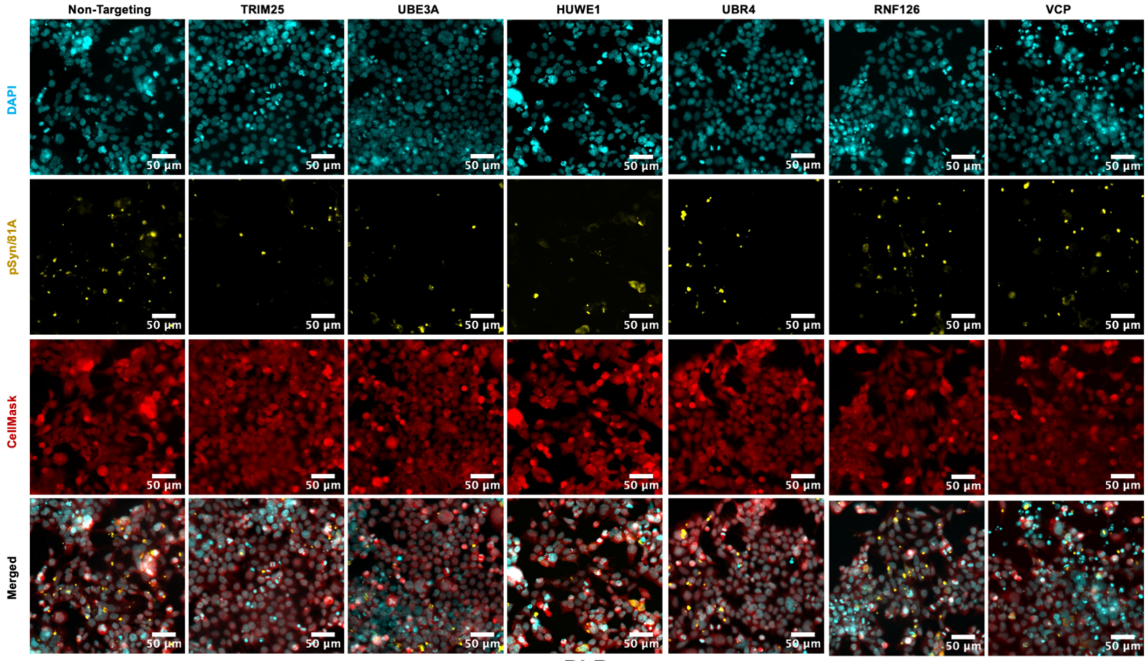

Appendix Figure S28. Functional testing of the effect of LiP-MS hits on αSyn accumulation upon uptake of DLB fibrils. The images show cells in which a single putative αSyn regulatory factor was genetically upregulated using CRISPR, followed by incubation for 72h with αSyn DLB fibrils. The fluorescence stains show pSer129 αSyn inclusions (pSer129/81A yellow), nuclei (DAPI, blue), and cytoplasm and nucleus (whole cell stain, red). NTG (Non-targeting control) indicates plasmids with a scrambled gRNA sequence. Scale bar 50 µm.

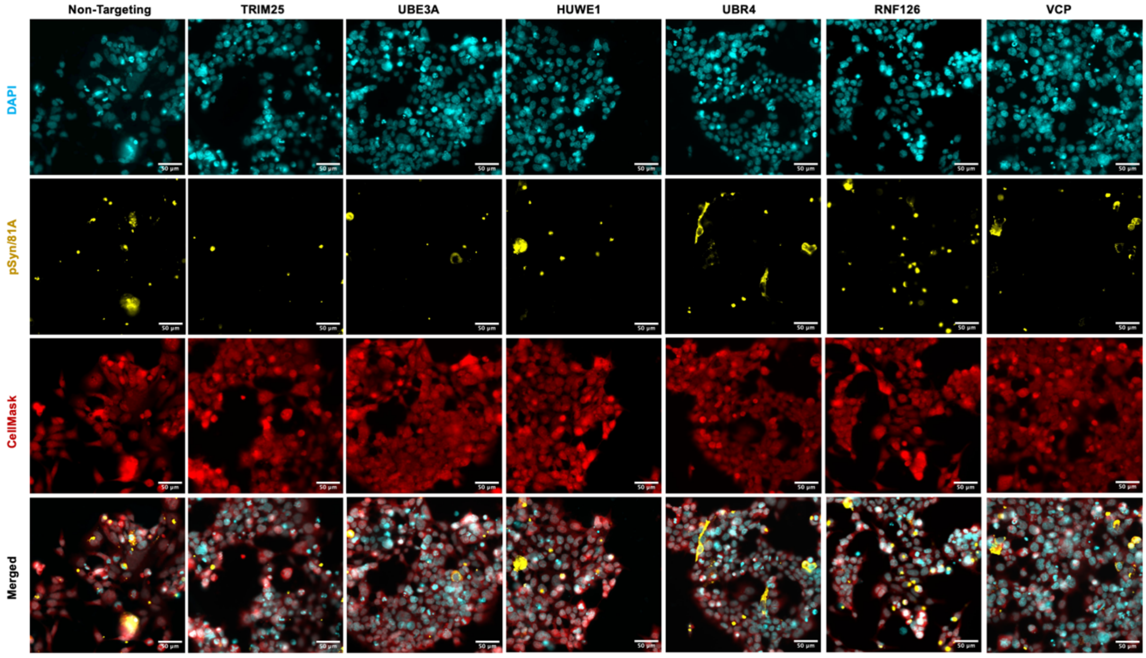

Appendix Figure S29. Functional testing of the effect of LiP-MS hits on αSyn accumulation upon uptake of MSA fibrils. The images show cells in which a single putative αSyn regulatory factor was genetically upregulated using CRISPR, followed by incubation for 72h with αSyn MSA fibrils. The fluorescence stains show pSer129 αSyn inclusions (pSer129/81A yellow), nuclei (DAPI, blue), and cytoplasm and nucleus (whole cell stain, red). NTG (Non-targeting control) indicates plasmids with a scrambled gRNA sequence. Scale bar 50 µm.

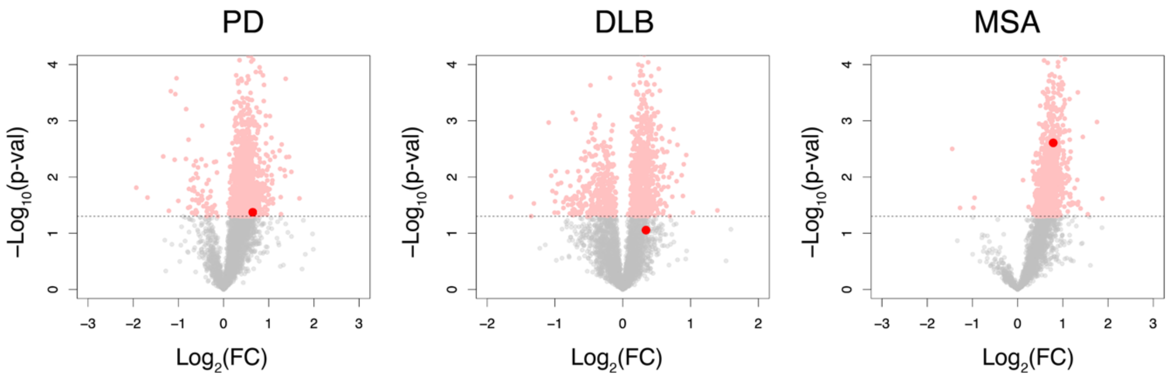

Appendix Figure S30. AP-MS validation of UBE3A interaction with the MSA and PD patient-derived fibrillar polymorph. UBE3A was immunoprecipitated from SH-SY5Y lysate that was pre-incubated with each of the αSyn fibrillar polymorph. The volcano plots show proteins that change upon anti-UBE3A immunoprecipitation versus isotype-specific IgG control; the red dot indicates αSyn and the significance threshold is indicated ($p\text{-val} < 0.05$).

	E3			DUBs	
**PD**	**DLB**	**MSA**	**PD**	**DLB**	**MSA**
DCAF13	FBXO22	HACE1	UCHL1	WDR48	WDR48
HUWE1	HUWE1	HUWE1	UCHL5	UCHL5	USP15
RANBP2	SKP1	IRF2BP1	USP7	UCHL1	USP5
SKP1	CUL4A	LRRC41	OTUB1	USP7	USP9X
UFL1	BIRC6	UHRF1	SENP3		VCPIP1
CAND1	CUL1	UBR4	UFSP2		OTUB1
DCUN1D1	TRIM28	UBE3A	HINT1		HINT1
UHRF1		RanBP2	USP13		UCHL1
UBR5		CACYBP	USP14		UCHL5
UBE4A		CDC23	USP5		USP11
FBXL18		CAND1			USP14
CACYBP		DDB1			USP4
CUL4B		ELOC			EIF3F
ELOC		HLTF			
PRPF19		LRRC41			
TRIM24		PRPF19			
TRIM28		TRIM28			
UBR4		UBR7			
UBE3A					

Appendix Table S1. Lists of E3 ligases and DUBs structurally responding to the disease fibrils after spiking into SH-SY5Y lysate for 15 min. Hits are colored (text) based on the significance cut off (black: FC>2, q-val<0.05; magenta: FC>1.5, q-val<0.05).

Proteases		
**PD**	**DLB**	**MSA**
COPS5	COPS5	AGTPBP1
XPNPEP1	RNPEP	XPNPEP1
NPEPPS	CAPN1	RNPEP
RNPEP	CPD	CPD
BLMH	MIPEP	CTSD
CNDP2	UCHL5	NUP98
ERAP1	UCHL1	TPP2
IDE	USP7	USP15
PMPCB	AGTPBP1	USP5
PSMC2	CLPP	USP9X
SCPEP1	THOP1	USP9Y
UCHL1		VCPIP1
UCHL5		AFG3L2
USP7		OTUB1
AGTPBP1		CNDP2
OTUB1		DPP3
PARK7		EIF3F
SENP3		HINT1
UFSP2		PEPD
CAPN1		PSMB6
CLPP		PSMC2
CTSD		PSMD14
HINT1		UCHL1
NLN		USP11
PSMD14		USP14
TPP2		USP4
USP13		
USP14		
USP5		

Appendix Table S2. Lists of proteases structurally responding to αSyn strains after spiking into SH-SY5Y lysate for 15 min. Hits are colored (text) based on the significance cut off (black: FC>2, q-val<0.05; magenta: FC>1.5, q-val<0.05).

Proteases		
**PD**	**DLB**	**MSA**
ACE	UFSP2	OTUD6B
BLMH	THOP1	NPEPPS
CPD	USP19	BLMH
ERAP1	USP47	CAPN1
HINT1	AFG3L2	CNDP2
AFG3L2	CLPP	CASP3
RNPEP	CTSL	CTSA
LTA4H		DPP3
LONP1		DPP9
PSMC2		IDE
USP14		NUP98
USP10		PREP
		UCHL1
		USP4
		ADAM10
		AFG3L2
		OTUB1
		PARK7
		UFSP2
		DPP6
		EIF3F
		HINT1
		NUP98
		PSMD14
		TPP2
		USP11
		USP35
		USP4

Appendix Table S3. Lists of proteases structurally responding to disease-derived αSyn fibrillar polymorph uptake by SH-SY5Y live cells. Hits are colored (text) based on the significance cut off (black: FC>2, q-val<0.05; magenta: FC>1.5, q-val<0.05).

Wikipathway	DLB hits Count	P value
WP534:Glycolysis and gluconeogenesis	10	1.05E-05
WP1946:Cori cycle	7	2.54E-05
WP4628:Aerobic glycolysis augmented	6	1.40E-04
WP4629:Aerobic glycolysis	6	1.40E-04
WP3888:VEGFA VEGFR2 signaling	27	2.26E-04
WP4290:Metabolic reprogramming in colon cancer	9	7.85E-04
WP5355:Metabolic epileptic disorders	11	0.0012922
WP383:Striated muscle contraction pathway	5	0.00187809
WP4018:Clear cell renal cell carcinoma pathways	9	0.00242735
WP5193:Cholesterol synthesis disorders	5	0.00657619
WP706:Sudden infant death syndrome SIDS susceptibility pathways	7	0.02174297
WP5426:HDAC6 interactions in the central nervous system	9	0.02179481
WP5333:Enterocyte cholesterol metabolism	5	0.0264029
WP289:Myometrial relaxation and contraction pathways	7	0.02683042
WP197:Cholesterol biosynthesis pathway	4	0.02831878
WP183:Proteasome degradation	7	0.0326534
WP2884:NRF2 pathway	6	0.03926318
WP5329:Cholesterol biosynthesis pathway in hepatocytes	5	0.04039139
WP2359:Parkin ubiquitin proteasomal system pathway	7	0.0592287

Appendix Table S4. Pathway enrichment analysis (WikiPathways) for the set of proteins that show structural changes in neurons treated with DLB fibrils (p-val <0.05).

Wikipathway	MSA hits Count	P value
WP183:Proteasome degradation	26	1.92E-06
WP5376:17p13 3 YWHAE copy number variation	11	6.21E-04
WP314:Fas ligand pathway and stress induction of heat shock proteins	13	9.72E-04
WP4718:Cholesterol metabolism with Bloch and Kandutsch Russell pathways	15	0.00177423
WP534:Glycolysis and gluconeogenesis	15	0.00177423
WP5193:Cholesterol synthesis disorders	10	0.00178096
WP3888:VEGFA VEGFR2 signaling	73	0.00213816
WP197:Cholesterol biosynthesis pathway	9	0.00261463
WP4629:Aerobic glycolysis	8	0.00381255
WP4190:Mevalonate arm of cholesterol biosynthesis pathway	8	0.00381255
WP4628:Aerobic glycolysis augmented	8	0.00381255
WP5333:Enterocyte cholesterol metabolism	12	0.00392345
WP5124:Alzheimer 39 s disease	43	0.00408537
WP2059:Alzheimer 39 s disease and miRNA effects	43	0.00408537
WP5355:Metabolic epileptic disorders	24	0.00555933
WP4290:Metabolic reprogramming in colon cancer	17	0.00656341
WP4018:Clear cell renal cell carcinoma pathways	19	0.00724847
WP2359:Parkin ubiquitin proteasomal system pathway	21	0.00762465
WP5329:Cholesterol biosynthesis pathway in hepatocytes	12	0.01280461
WP1946:Cori cycle	8	0.01298829
WP5304:Cholesterol metabolism	14	0.02001194
WP4949:16p11 2 proximal deletion syndrome	15	0.02333649
WP5153:N glycan biosynthesis	12	0.02418329
WP3963:Mevalonate pathway	5	0.02520266
WP4804:Cholesterol biosynthesis with skeletal dysplasias	5	0.02520266
WP5114:Nucleotide excision repair in xeroderma pigmentosum	18	0.03186722
WP3925:Amino acid metabolism	21	0.0322771
WP2369:Histone modifications	14	0.03315797
WP698:Glucuronidation	5	0.04852685
WP3871:Valproic acid pathway	5	0.04852685

Appendix Table S5 Pathway enrichment analysis (WikiPathways) for the set of proteins that show structural changes in neurons treated with MSA fibrils (p-val <0.05).

Wikipathway	PD hits Count	P value
WP2267~Synaptic vesicle pathway	20	4.65E-07
WP534~Glycolysis and gluconeogenesis	13	0.00121081
WP706~Sudden infant death syndrome (SIDS) susceptibility pathways	17	0.00249845
WP5352~10q11.21q11.23 copy number variation syndrome	13	0.00549347
WP4290~Metabolic reprogramming in colon cancer	13	0.00709031
WP5233~Arsenic metabolism and reactive oxygen species generation	6	0.0123602
WP78~TCA cycle (aka Krebs or citric acid cycle)	8	0.01556097
WP536~Calcium regulation in cardiac cells	20	0.01605956
WP500~Glycogen synthesis and degradation	10	0.01894123
WP383~Striated muscle contraction pathway	6	0.01976094
WP289~Myometrial relaxation and contraction pathways	18	0.02116647
WP2359~Parkin-ubiquitin proteasomal system pathway	14	0.03152441
WP4932~7q11.23 copy number variation syndrome	13	0.03619362
WP4756~Renin-angiotensin-aldosterone system (RAAS)	7	0.04016533
WP4698~Vitamin D-sensitive calcium signaling in depression	7	0.04016533
WP3298~Melatonin metabolism and effects	5	0.04053488
WP3679~Cell-type dependent selectivity of CCK2R signaling	5	0.04053488
WP2118~Arrhythmogenic right ventricular cardiomyopathy	11	0.04776238
WP5124~Alzheimer's disease	29	0.04855935
WP2059~Alzheimer's disease and miRNA effects	29	0.04855935

Appendix Table S6. Pathways enrichment analysis (WikiPathways) for the set of proteins that show structural changes in patient brains afflicted by PD (p-val <0.05).

Wikipathway	DLB hits Count	P value
WP534~Glycolysis and gluconeogenesis	19	6.63E-08
WP4290~Metabolic reprogramming in colon cancer	17	6.23E-05
WP706~Sudden infant death syndrome (SIDS) susceptibility pathways	18	0.00127209
WP2267~Synaptic vesicle pathway	15	0.00130544
WP2359~Parkin-ubiquitin proteasomal system pathway	17	0.00282581
WP4018~Clear cell renal cell carcinoma pathways	16	0.00297169
WP4917~Proximal tubule transport	9	0.00302602
WP4629~Aerobic glycolysis	7	0.00718467
WP5049~Glycolysis in senescence	6	0.00824285
WP1946~Cori cycle	7	0.01152649
WP2272~Pathogenic Escherichia coli infection	13	0.01183644
WP289~Myometrial relaxation and contraction pathways	19	0.01389298
WP2059~Alzheimer's disease and miRNA effects	32	0.01567581
WP5124~Alzheimer's disease	32	0.01567581
WP3888~VEGFA-VEGFR2 signaling	50	0.01644492
WP2431~Spinal cord injury	14	0.02023907
WP536~Calcium regulation in cardiac cells	20	0.02257138
WP5344~Cardiomyocyte signaling pathways converging on Titin	6	0.02273813
WP5220~Metabolic reprogramming in pancreatic cancer	10	0.04537814

Appendix Table S7. Pathways enrichment analysis (WikiPathways) for the set of proteins that show structural changes in patient brains afflicted by DLB (p-val <0.05).

Wikipathway	MSA hits Count	P value
WP706~Sudden infant death syndrome (SIDS) susceptibility pathways	30	4.73E-04
WP4290~Metabolic reprogramming in colon cancer	22	9.36E-04
WP534~Glycolysis and gluconeogenesis	20	0.00106238
WP2267~Synaptic vesicle pathway	24	0.00133559
WP2359~Parkin-ubiquitin proteasomal system pathway	28	0.00141021
WP4148~Splicing factor NOVA regulated synaptic proteins	20	0.00187179
WP4767~FGFR3 signaling in chondrocyte proliferation and terminal differentiation	8	0.00313195
WP5220~Metabolic reprogramming in pancreatic cancer	19	0.00343762
WP3925~Amino acid metabolism	32	0.00396205
WP4629~Aerobic glycolysis	10	0.00480077
WP5114~Nucleotide excision repair in xeroderma pigmentosum	20	0.00506305
WP4018~Clear cell renal cell carcinoma pathways	25	0.00648689
WP2369~Histone modifications	13	0.00953743
WP5049~Glycolysis in senescence	8	0.00990353
WP2453~TCA cycle and deficiency of pyruvate dehydrogenase complex (PDHc)	11	0.01062892
WP106~Alanine and aspartate metabolism	9	0.01071271
WP1946~Cori cycle	10	0.01087119
WP5085~Vasopressin-regulated water reabsorption	16	0.01215565
WP4159~GABA receptor signaling	14	0.01495281
WP78~TCA cycle (aka Krebs or citric acid cycle)	12	0.01810431
WP3871~Valproic acid pathway	6	0.02043411
WP179~Cell cycle	14	0.0237743
WP4549~Fragile X syndrome	42	0.02512852
WP2864~Apoptosis-related network due to altered Notch3 in ovarian cancer	13	0.02667579
WP5046~NAD metabolism in oncogene-induced senescence and mitochondrial dysfunction-associated senescence	10	0.03719724
WP98~Prostaglandin synthesis and regulation	10	0.03719724
WP500~Glycogen synthesis and degradation	16	0.03996587
WP2064~Neural crest differentiation	9	0.04120123
WP3888~VEGFA-VEGFR2 signaling	98	0.04228711

Appendix Table S8. Pathways enrichment analysis (WikiPathways) for the set of proteins that show structural changes in patient brains afflicted by MSA (p-val <0.05).

	E3			DUBs	
**PD**	**DLB**	**MSA**	**PD**	**DLB**	**MSA**
FBXO41	BRCC3	COMMD4	UCHL1	MINDY1	MINDY2
HECTD3	FBXO2	FBXL16	**USP14**	OTUB1	**OTUB1**
CUL1	FBXO41	FBXO2	USP5	HINT1	**EIF3F**
**CUL3**	FBXO44	FBXO41		**UCHL1**	**HINT1**
CUL4B	HUWE1	HACE1		USP5	**UCHL1**
**CAND1**	SKP1	HECW2			UCHL3
DDB1	UBAC1	**HUWE1**			UCHL5
ITCH	WSB2	**RANBP2**			USP10
MARCHF5	CUL3	SKP1			USP14
TRIM2	CUL4B	SUGT1			USP15
	DDB1	UBAC1			USP46
	MUL1	**UFL1**			USP47
	**UBR4**	CACYBP			USP5
	UBE4A	CUL1			USP9X
	**TRIM28**	**CUL2**			
		CUL4B			
		**CUL5**			
		**CAND1**			
		DDB1			
		GAN			
		RNF123			
		RBX1			
		**TRIM2**			
		**TRIM25**			
		**TRIM28**			
		UBR1			
		**UBR4**			
		UBE3C			
		UBE4A			

Appendix Table S9. Lists of E3 ligases and DUBs structurally altered in brains of patients suffering from PD, DLB, and MSA. Hits are colored (text) based on the significance cut off (black: FC>2, q-val<0.05; magenta: FC>1.5, q-val<0.05). Orange, cyan, and red shades highlight the overlapping LiP-MS hits (i.e., proteins that show structural changes) between the cell seeding experiment in SH-SY5Y cells (orange), in neurons (cyan), or in both (red) and the comparison of brain proteomes (q-val< 0.05, FC >1.5).

Sample	Repl. 1 Fold change mRNA	Repl. 2 Fold change mRNA	Fold change Protein level
TRIM25	2.1	2.9	2.2
UBE3A	2.0	2.1	1.6
VCP	2.4	11.0	1.5
HUWE1	21.4	2.6	1.1
RNF126	2.0	1.6	1.5
UBR4	1.2	1.9	1.1

Appendix Table S10. Control of gene activation in CRISPR-edited HEK293 cells. Fold change of mRNA and protein levels in HEK cells where corresponding gene was activated.
