# Supplementary figures and images for: Structure-function relationship of alpha-synuclein fibrillar polymorphs derived from distinct synucleinopathies
Source: Mol Syst Biol. 2026 Mar 11;22(6):868–901. doi: 10.1038/s44320-026-00199-5 (PMC13230553; doi:10.1038/s44320-026-00199-5)

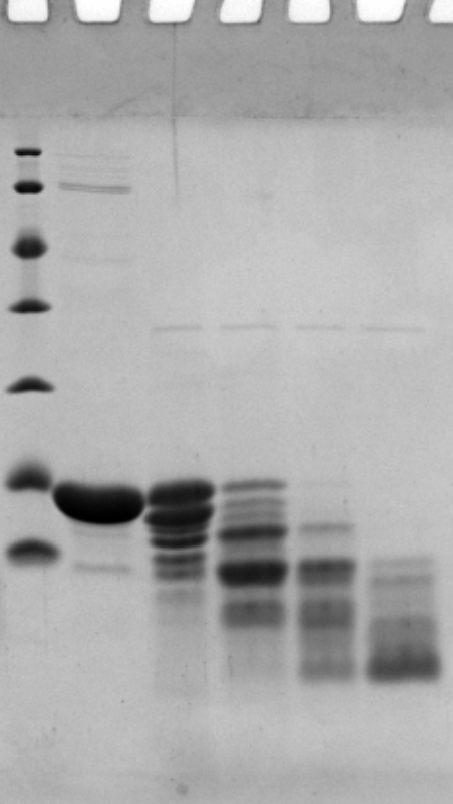

Supplement: Supplementary file 23 — Figure EV1 Source Data [file 44320_2026_199_MOESM23_ESM.zip › Figure_EV1/EV1B/Gel_DLB362_PK.tif]

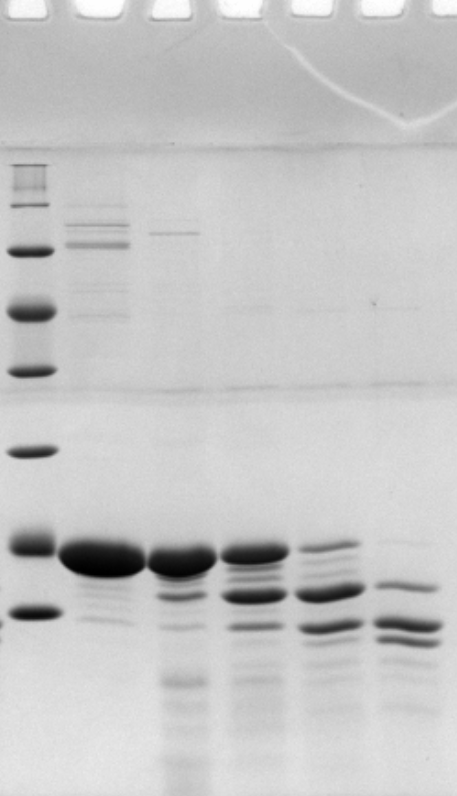

Supplement: Supplementary file 23 — Figure EV1 Source Data [file 44320_2026_199_MOESM23_ESM.zip › Figure_EV1/EV1B/Gel_PD341_PK.tif]

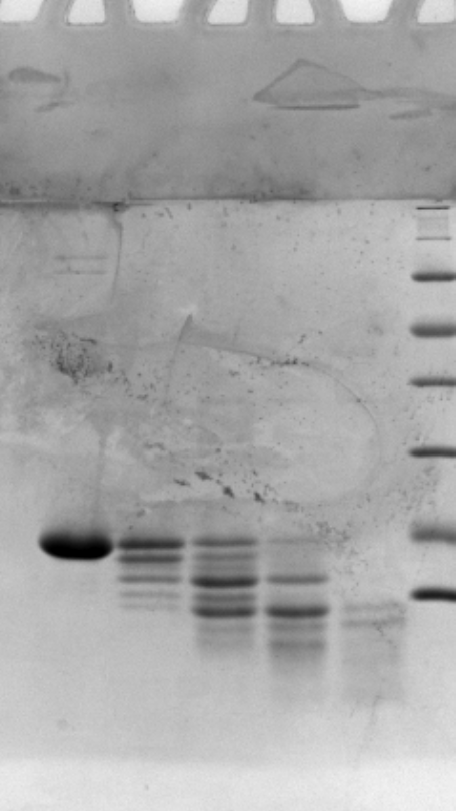

Supplement: Supplementary file 23 — Figure EV1 Source Data [file 44320_2026_199_MOESM23_ESM.zip › Figure_EV1/EV1B/Gel_DLB163_PK.tif]

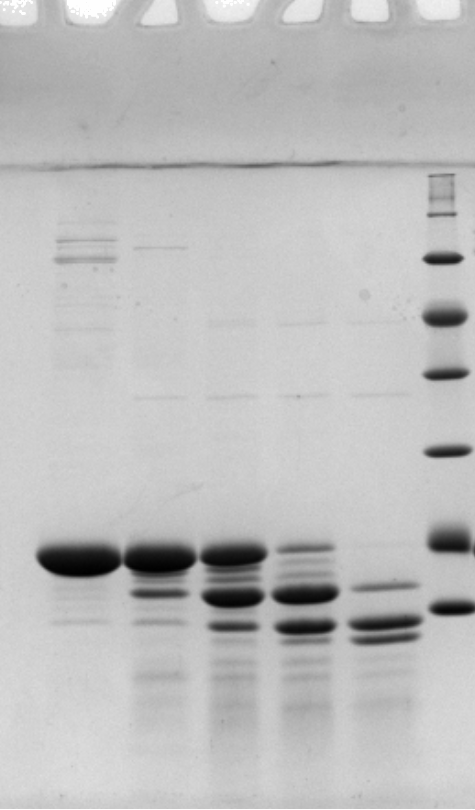

Supplement: Supplementary file 23 — Figure EV1 Source Data [file 44320_2026_199_MOESM23_ESM.zip › Figure_EV1/EV1B/Gel_MSA043_PK.tif]

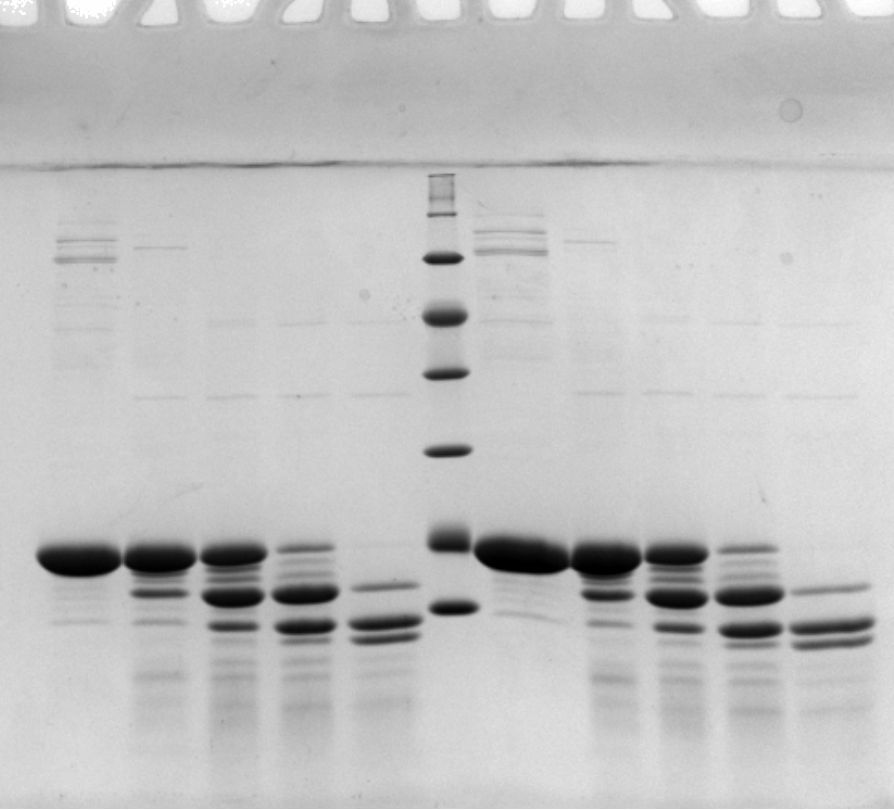

Supplement: Supplementary file 23 — Figure EV1 Source Data [file 44320_2026_199_MOESM23_ESM.zip › Figure_EV1/EV1B/Gel_MSA043 and MSA363_PK.tif]

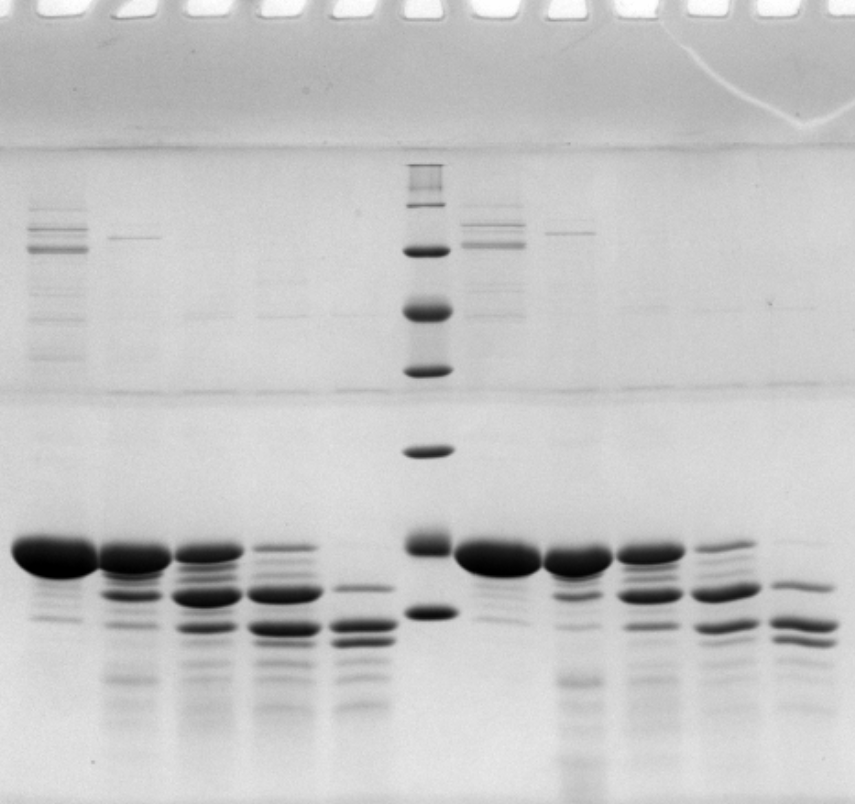

Supplement: Supplementary file 23 — Figure EV1 Source Data [file 44320_2026_199_MOESM23_ESM.zip › Figure_EV1/EV1B/Gel_PD258 and PD341_PK.tif]

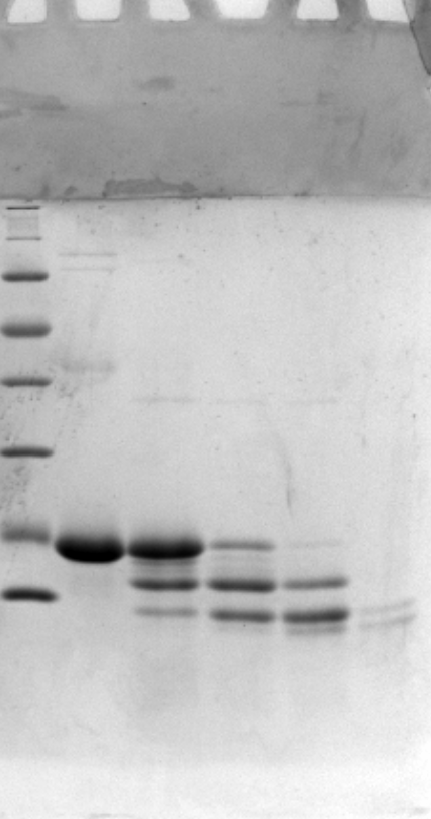

Supplement: Supplementary file 23 — Figure EV1 Source Data [file 44320_2026_199_MOESM23_ESM.zip › Figure_EV1/EV1B/Gel_MSA080_PK.tif]

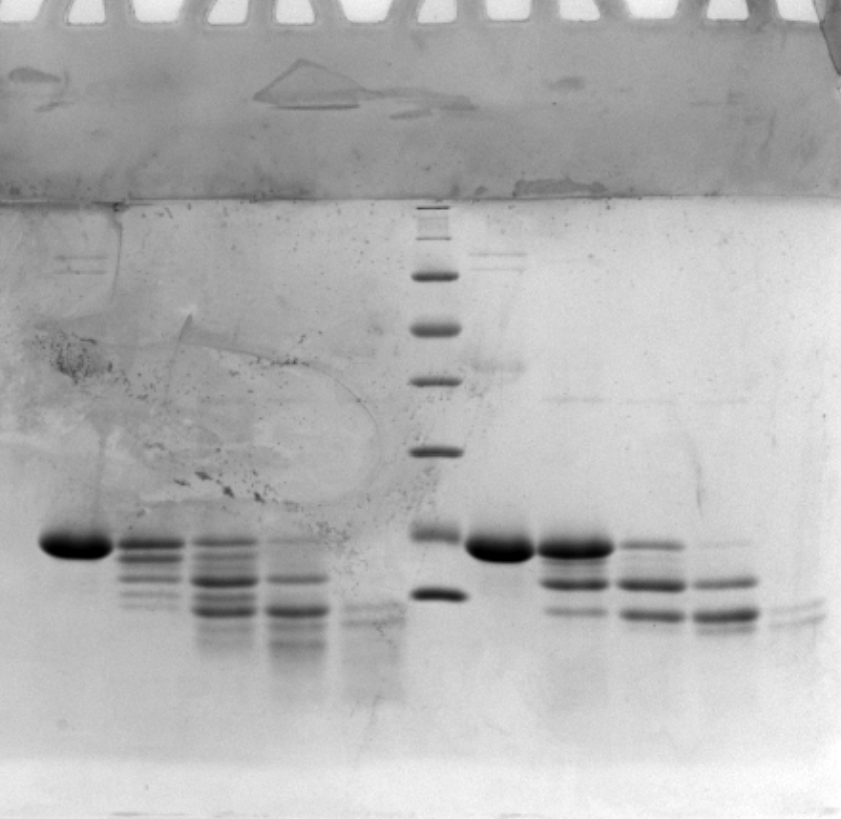

Supplement: Supplementary file 23 — Figure EV1 Source Data [file 44320_2026_199_MOESM23_ESM.zip › Figure_EV1/EV1B/Gel_DLB163 and MSA080_PK.tif]

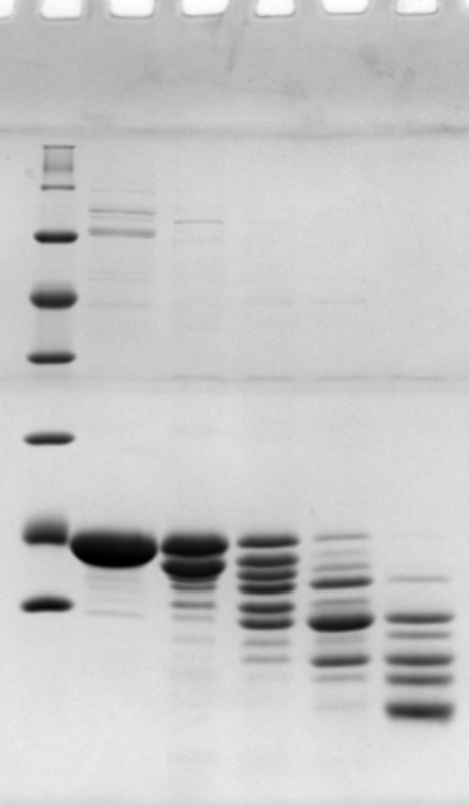

Supplement: Supplementary file 23 — Figure EV1 Source Data [file 44320_2026_199_MOESM23_ESM.zip › Figure_EV1/EV1B/Gel_DLB330_PK.tif]

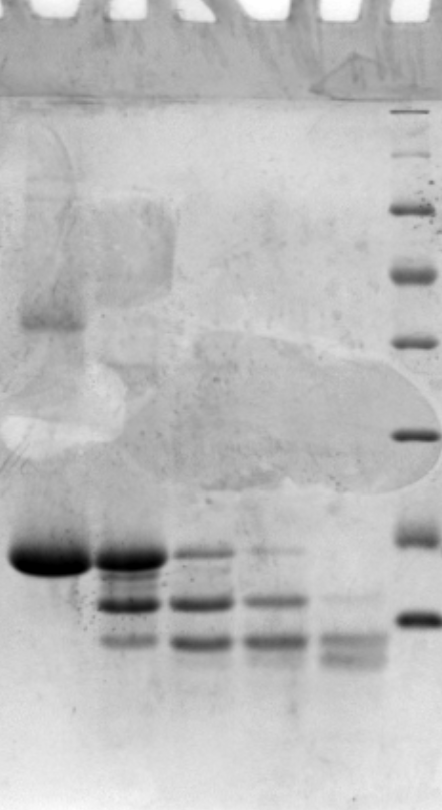

Supplement: Supplementary file 23 — Figure EV1 Source Data [file 44320_2026_199_MOESM23_ESM.zip › Figure_EV1/EV1B/Gel_PD405_PK.tif]

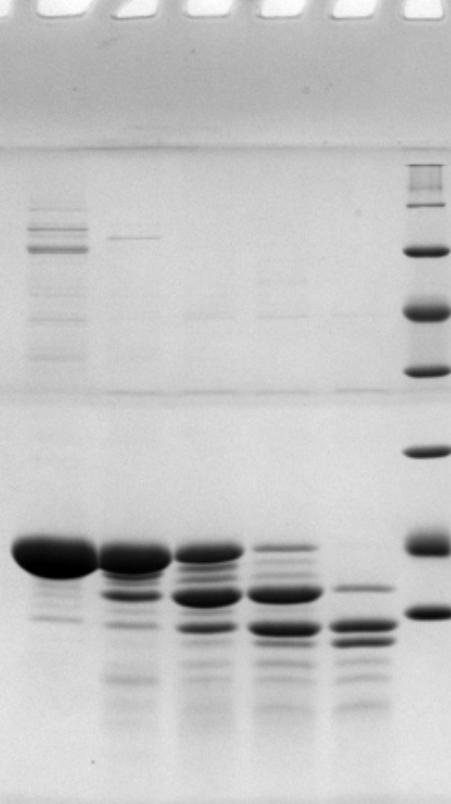

Supplement: Supplementary file 23 — Figure EV1 Source Data [file 44320_2026_199_MOESM23_ESM.zip › Figure_EV1/EV1B/Gel_PD258_PK.tif]

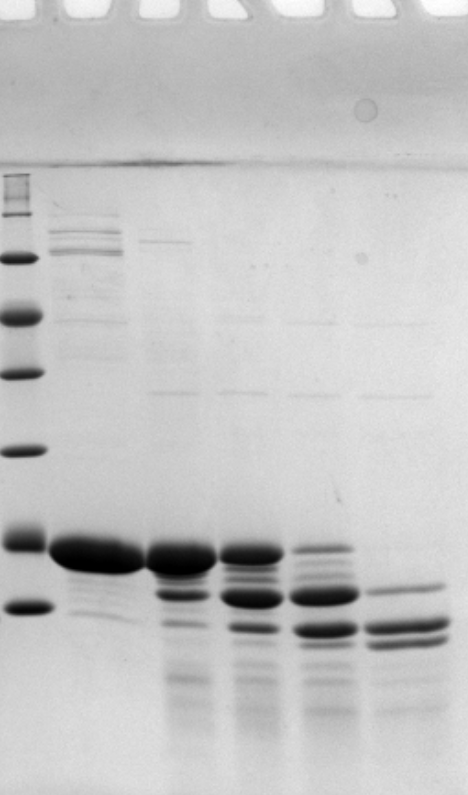

Supplement: Supplementary file 23 — Figure EV1 Source Data [file 44320_2026_199_MOESM23_ESM.zip › Figure_EV1/EV1B/Gel_MSA363_PK.tif]

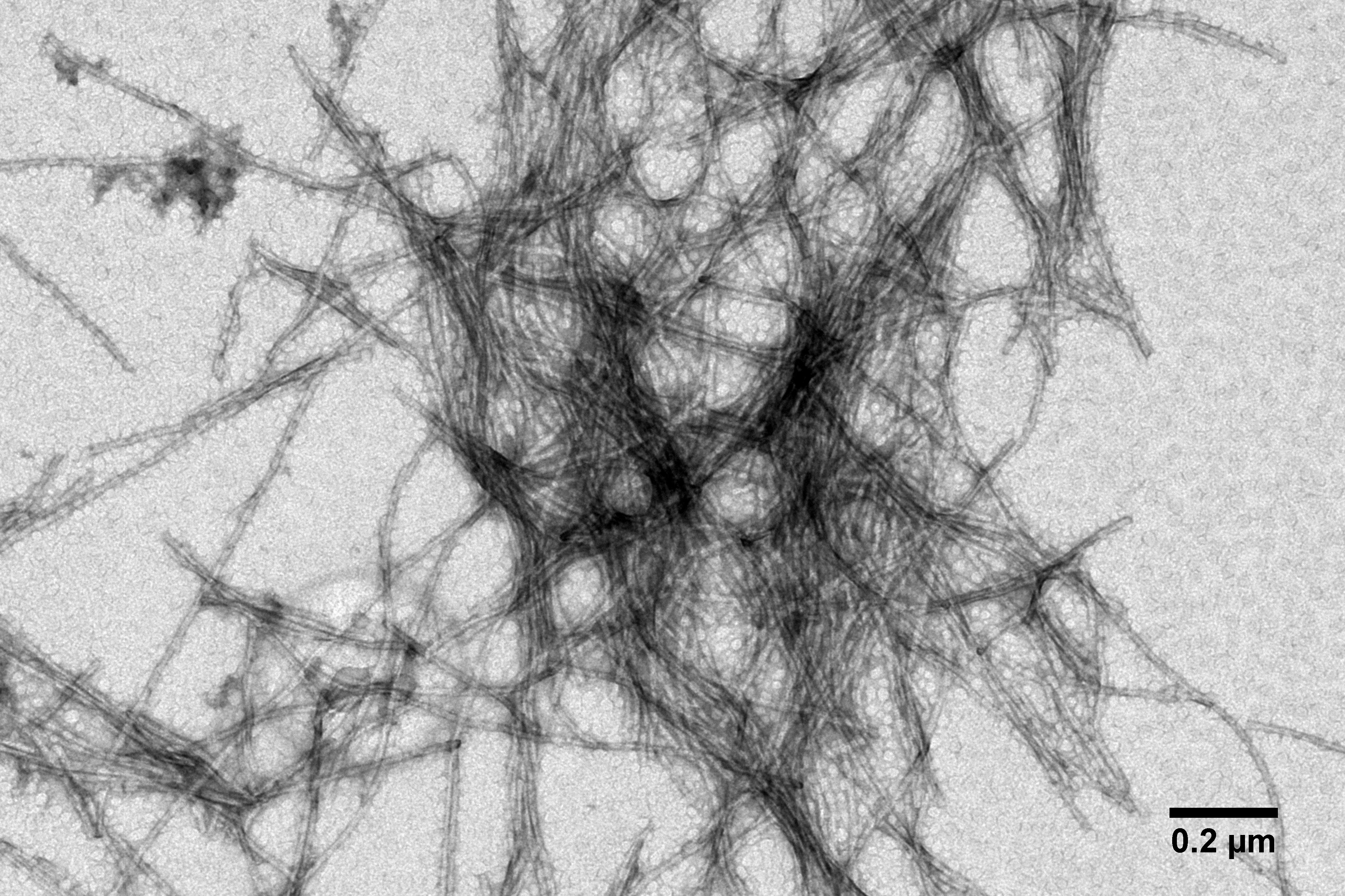

Supplement: Supplementary file 23 — Figure EV1 Source Data [file 44320_2026_199_MOESM23_ESM.zip › Figure_EV1/EV1A/PD405.jpg]

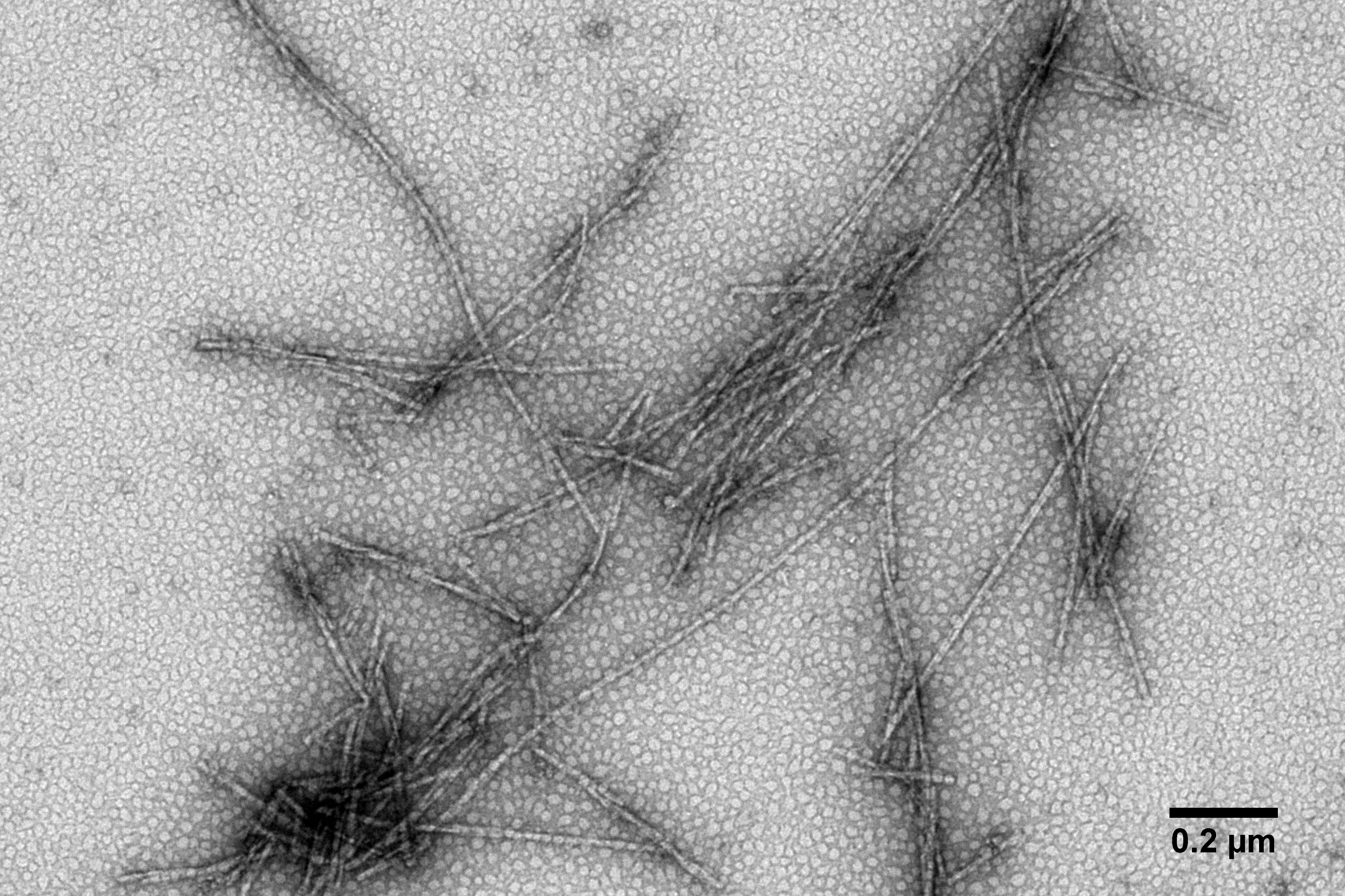

Supplement: Supplementary file 23 — Figure EV1 Source Data [file 44320_2026_199_MOESM23_ESM.zip › Figure_EV1/EV1A/DLB163.jpg]

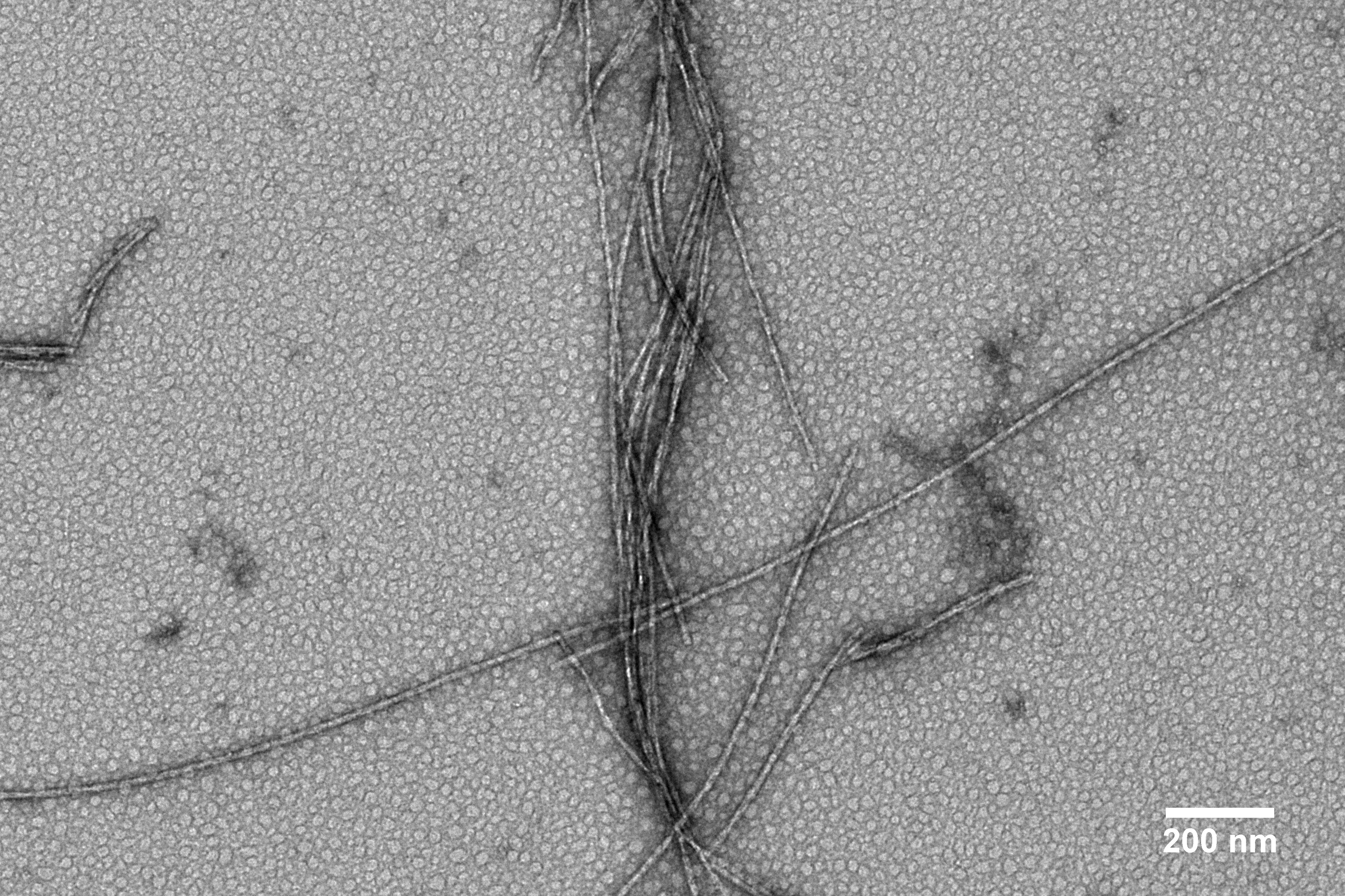

Supplement: Supplementary file 23 — Figure EV1 Source Data [file 44320_2026_199_MOESM23_ESM.zip › Figure_EV1/EV1A/DLB362.jpg]

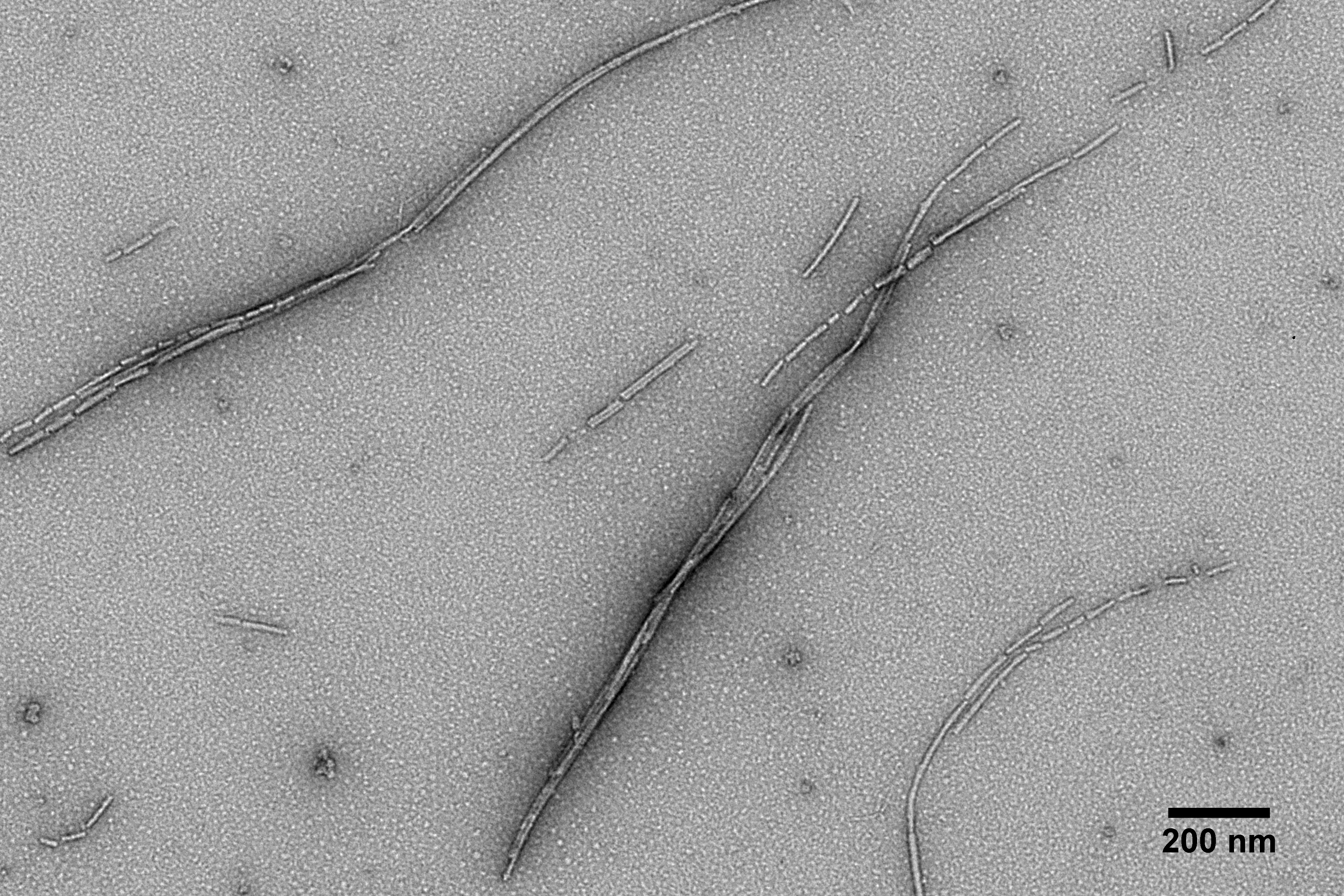

Supplement: Supplementary file 23 — Figure EV1 Source Data [file 44320_2026_199_MOESM23_ESM.zip › Figure_EV1/EV1A/MSA363.jpg]

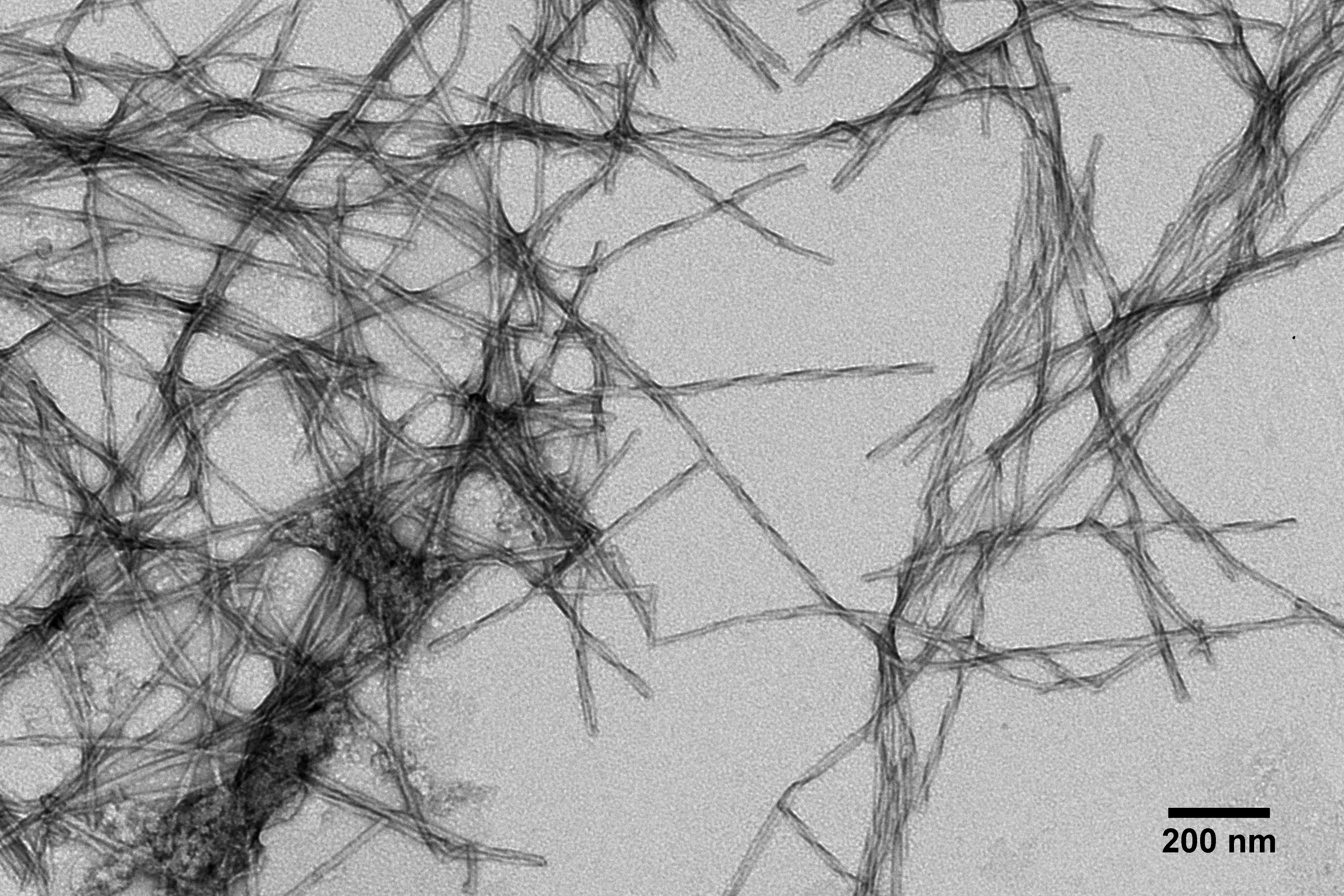

Supplement: Supplementary file 23 — Figure EV1 Source Data [file 44320_2026_199_MOESM23_ESM.zip › Figure_EV1/EV1A/PD258.jpg]

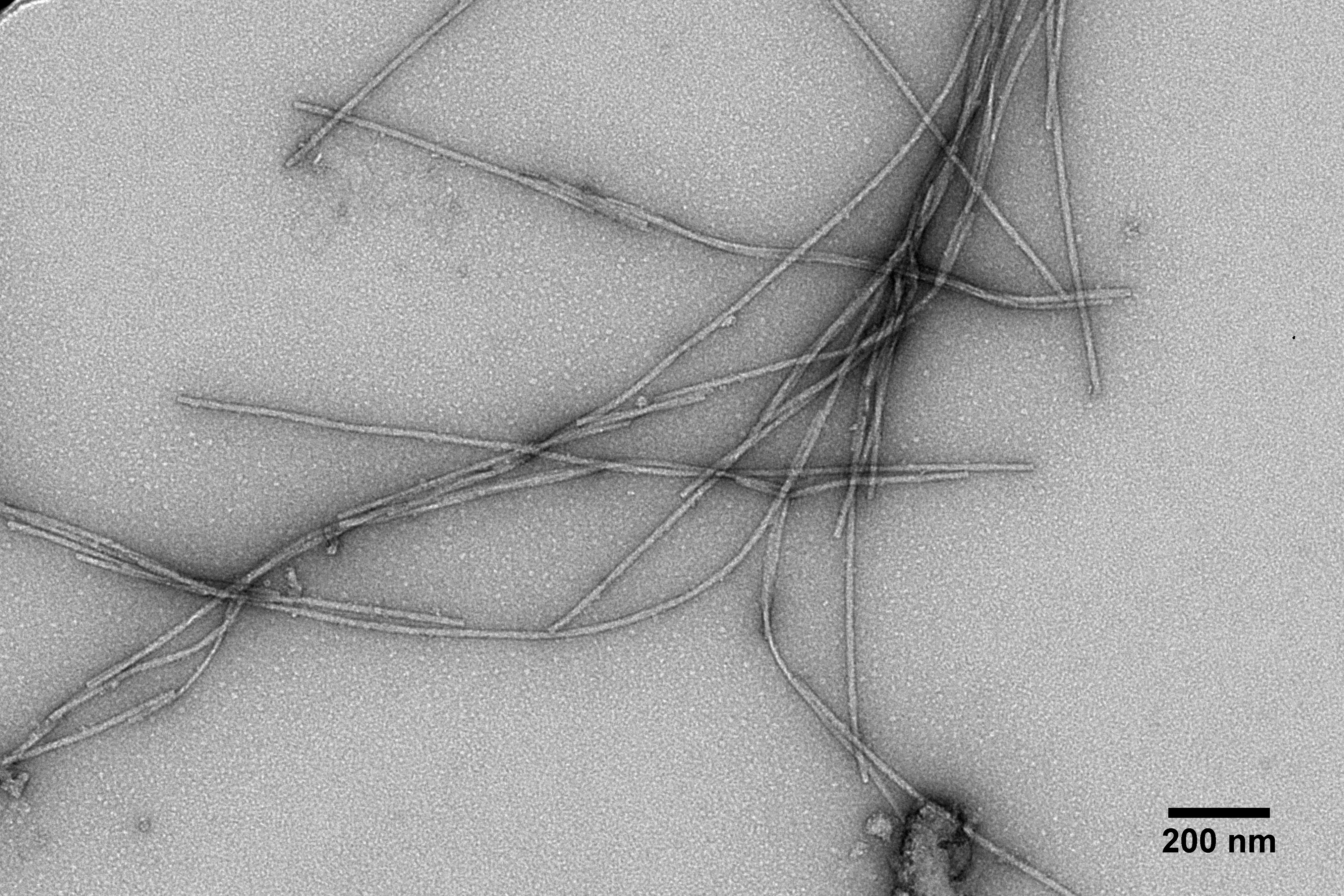

Supplement: Supplementary file 23 — Figure EV1 Source Data [file 44320_2026_199_MOESM23_ESM.zip › Figure_EV1/EV1A/DLB330.jpg]

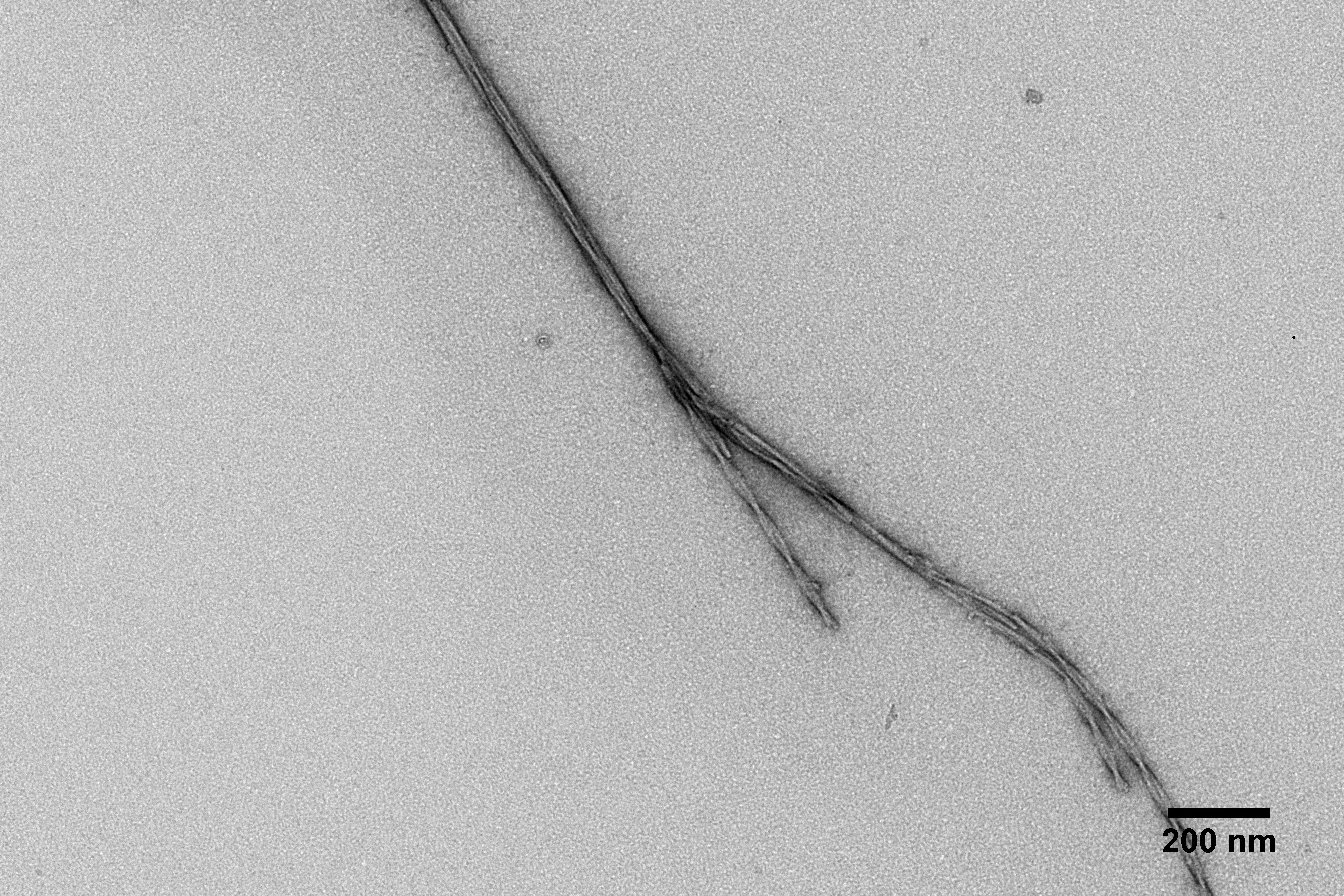

Supplement: Supplementary file 23 — Figure EV1 Source Data [file 44320_2026_199_MOESM23_ESM.zip › Figure_EV1/EV1A/PD341.jpg]

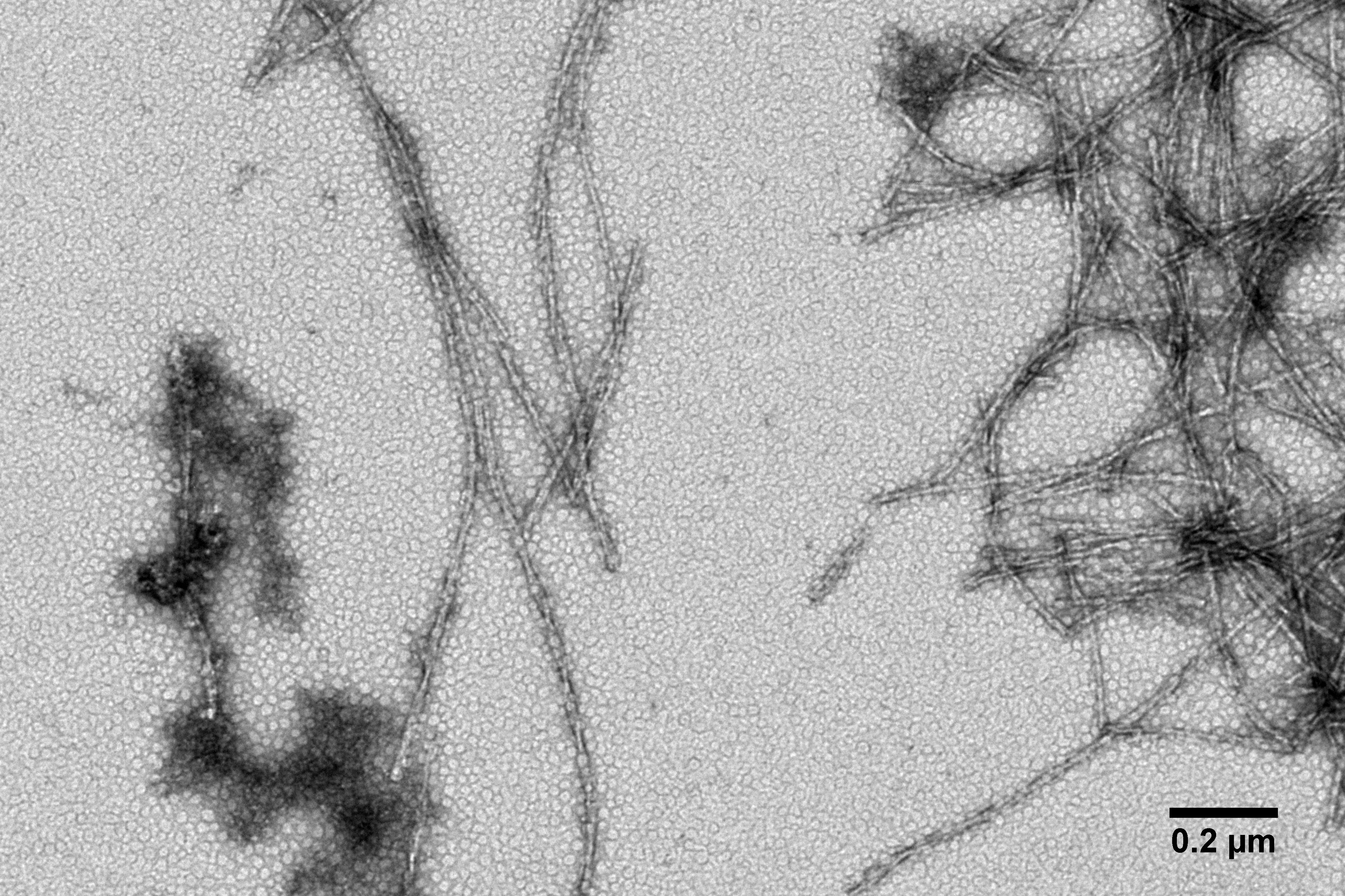

Supplement: Supplementary file 23 — Figure EV1 Source Data [file 44320_2026_199_MOESM23_ESM.zip › Figure_EV1/EV1A/MSA080.jpg]

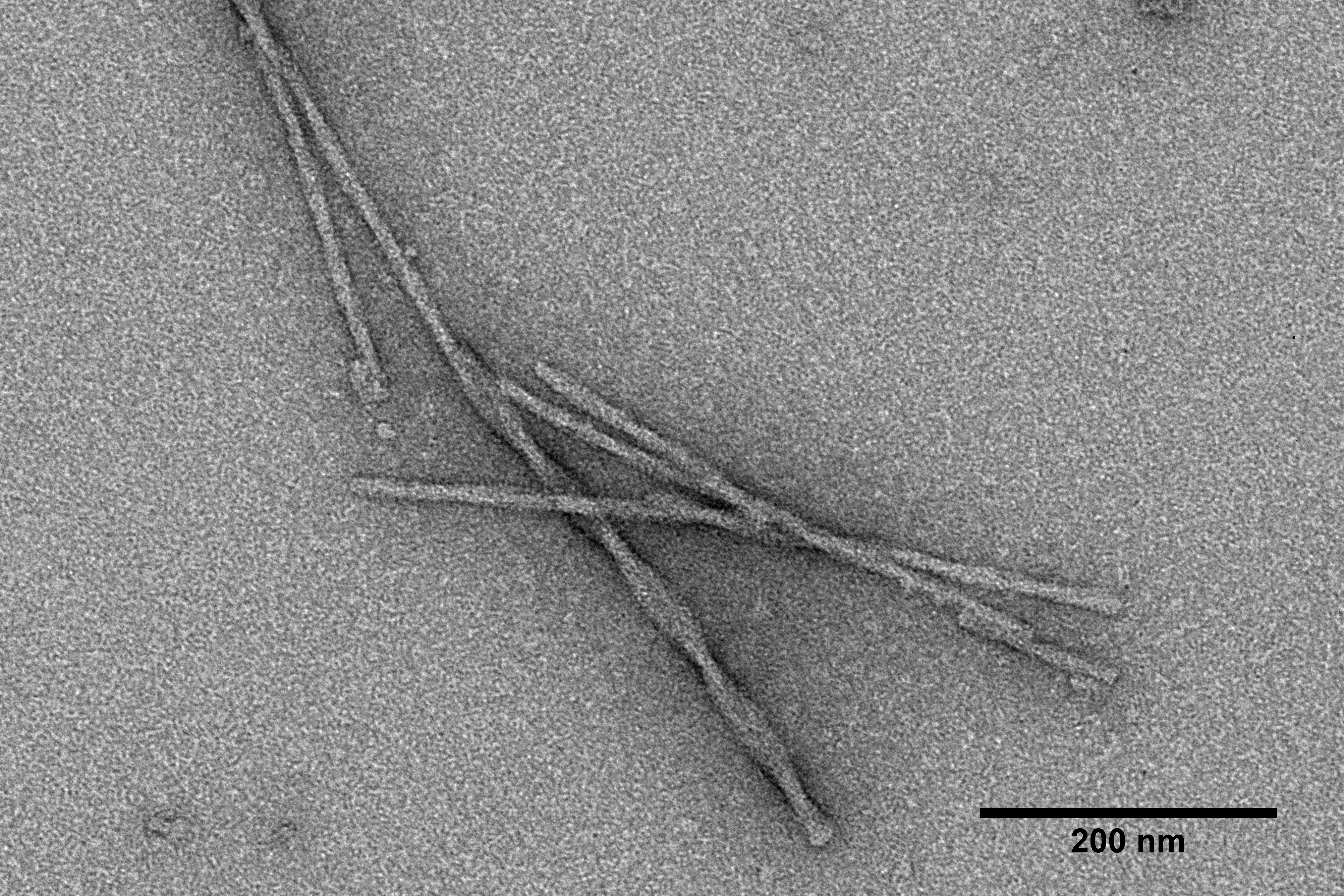

Supplement: Supplementary file 23 — Figure EV1 Source Data [file 44320_2026_199_MOESM23_ESM.zip › Figure_EV1/EV1A/MSA043.jpg]

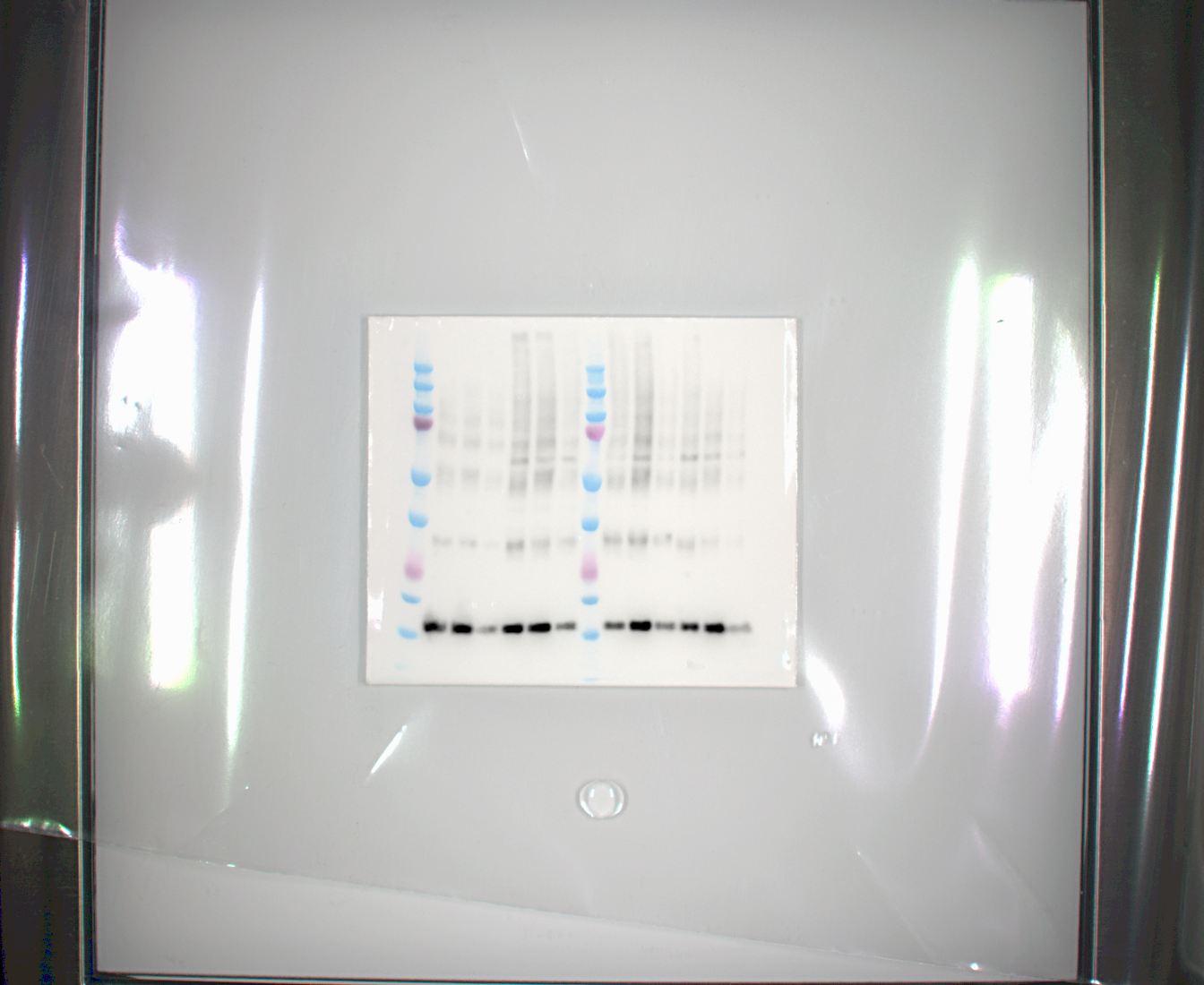

Supplement: Supplementary file 24 — Source data Fig. 2 [file 44320_2026_199_MOESM24_ESM.zip › Figure_2D_alpha_synuclein.png]

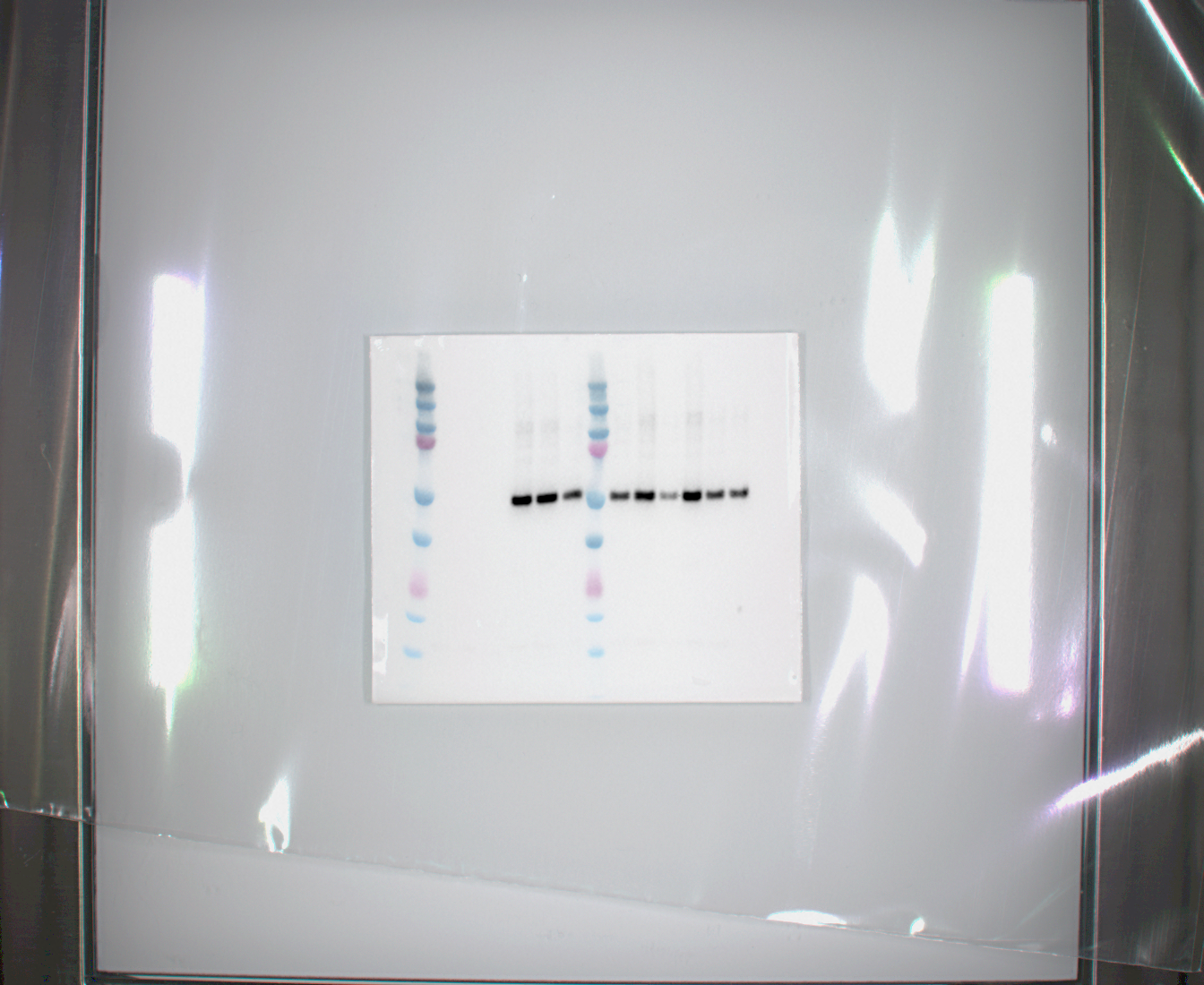

Supplement: Supplementary file 24 — Source data Fig. 2 [file 44320_2026_199_MOESM24_ESM.zip › Figure_2D_alpha_tubulin.png]

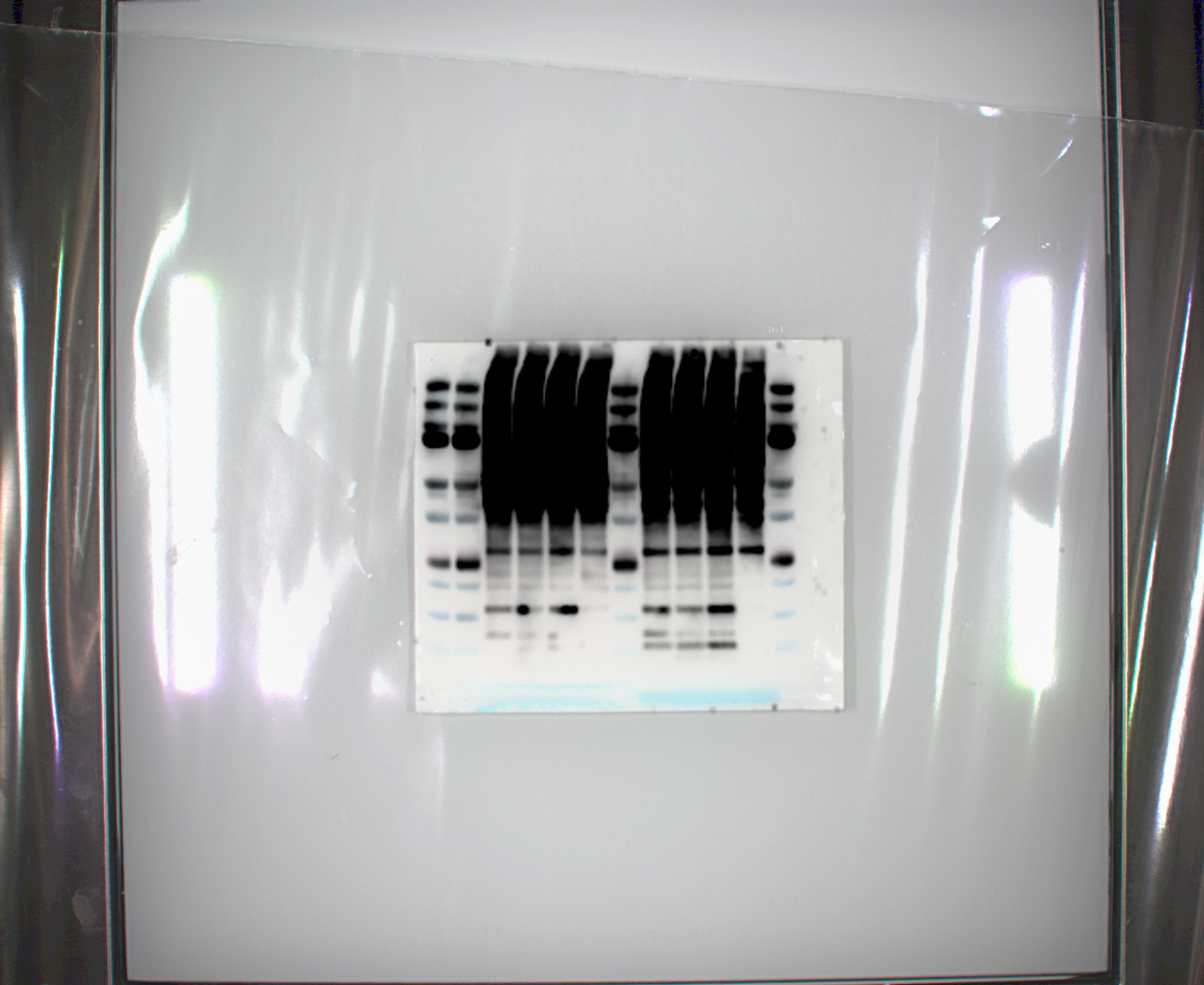

Supplement: Supplementary file 26 — Source data Fig. 4 [file 44320_2026_199_MOESM26_ESM.zip › Figure_4/Figure_4C_alpha_synuclein.Tif]

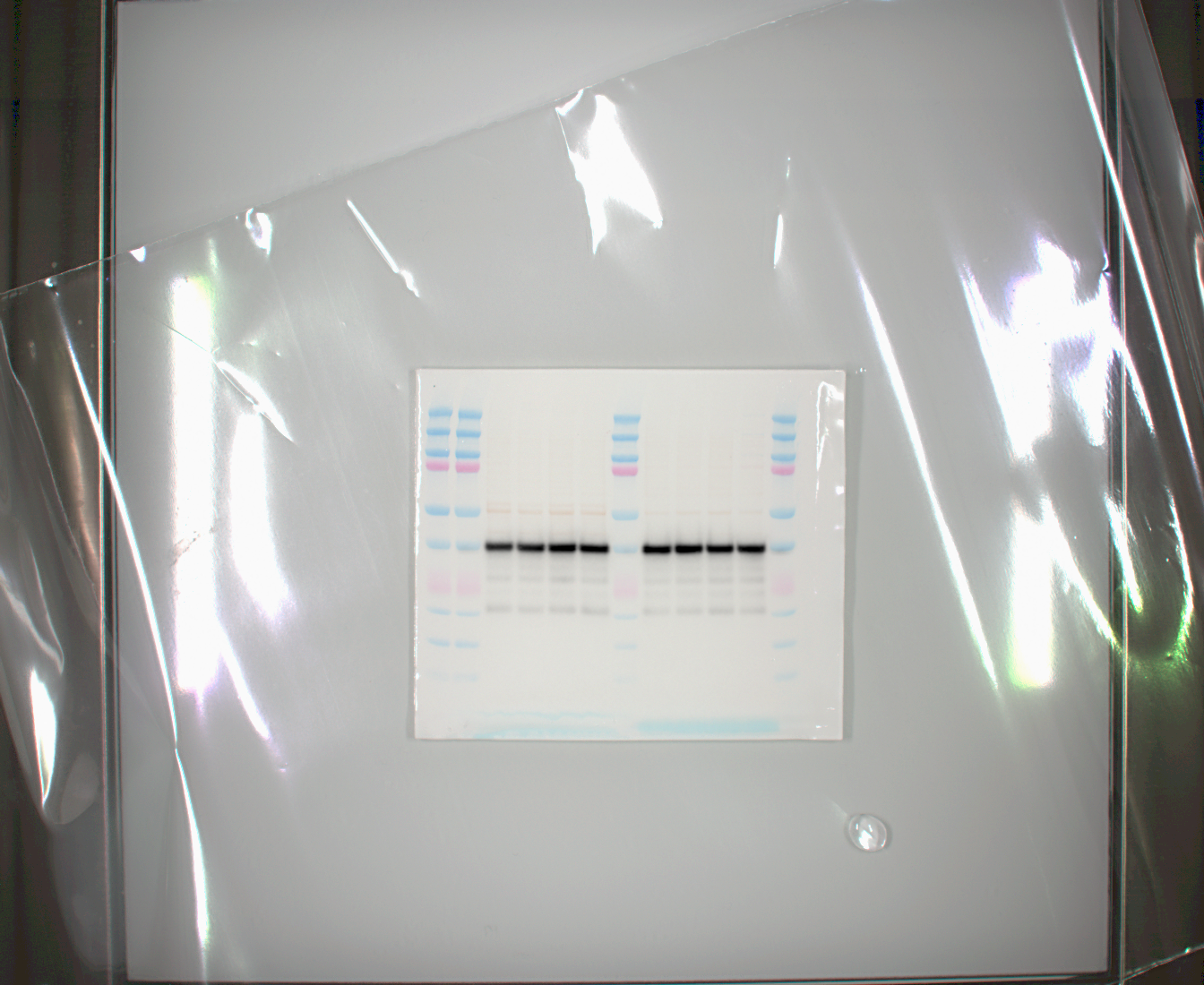

Supplement: Supplementary file 26 — Source data Fig. 4 [file 44320_2026_199_MOESM26_ESM.zip › Figure_4/Figure_4C_GAPDH.Tif]

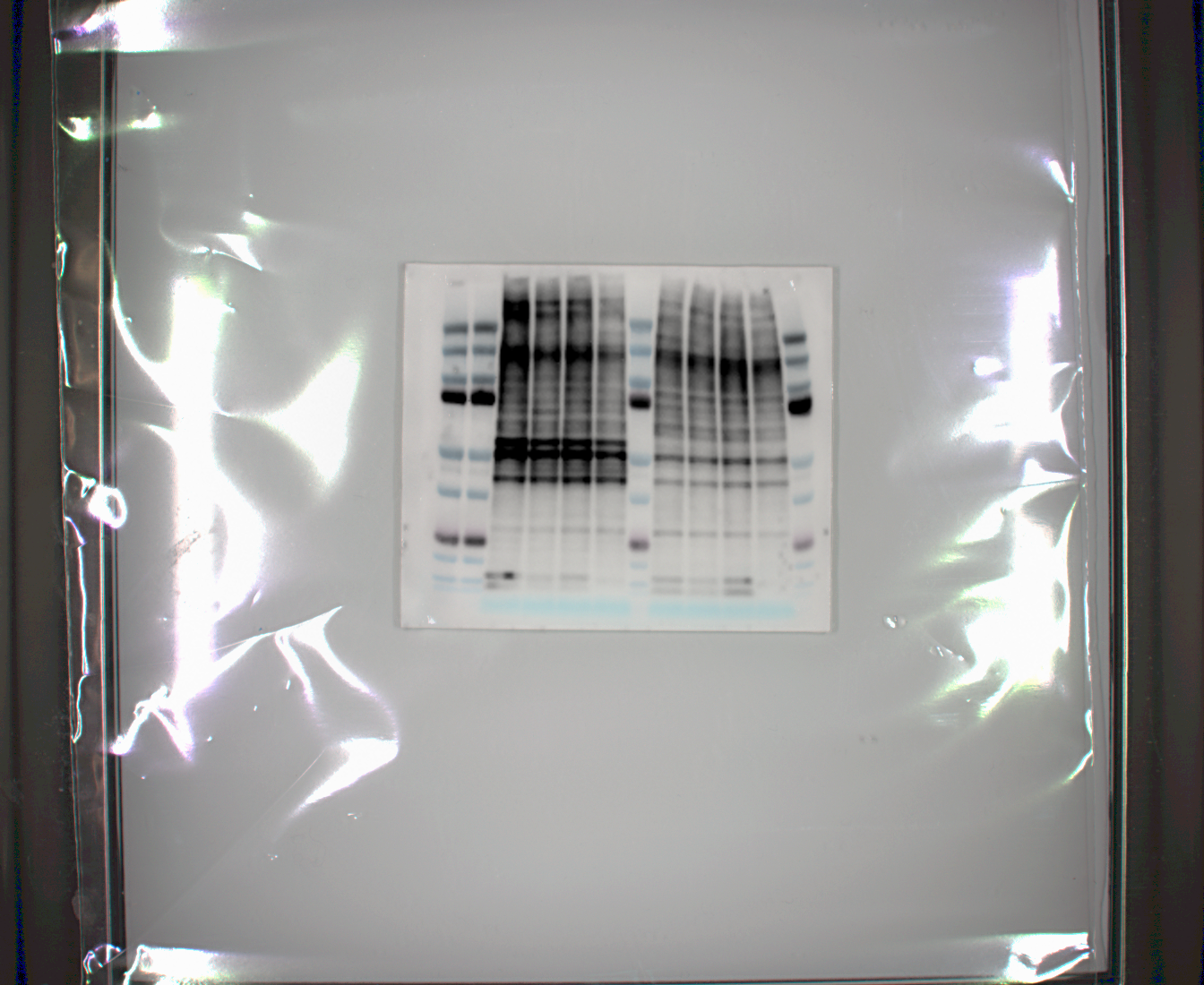

Supplement: Supplementary file 27 — Source data Fig. 5 [file 44320_2026_199_MOESM27_ESM.zip › Figure_5/Figure_5D_alpha_synuclein.Tif]

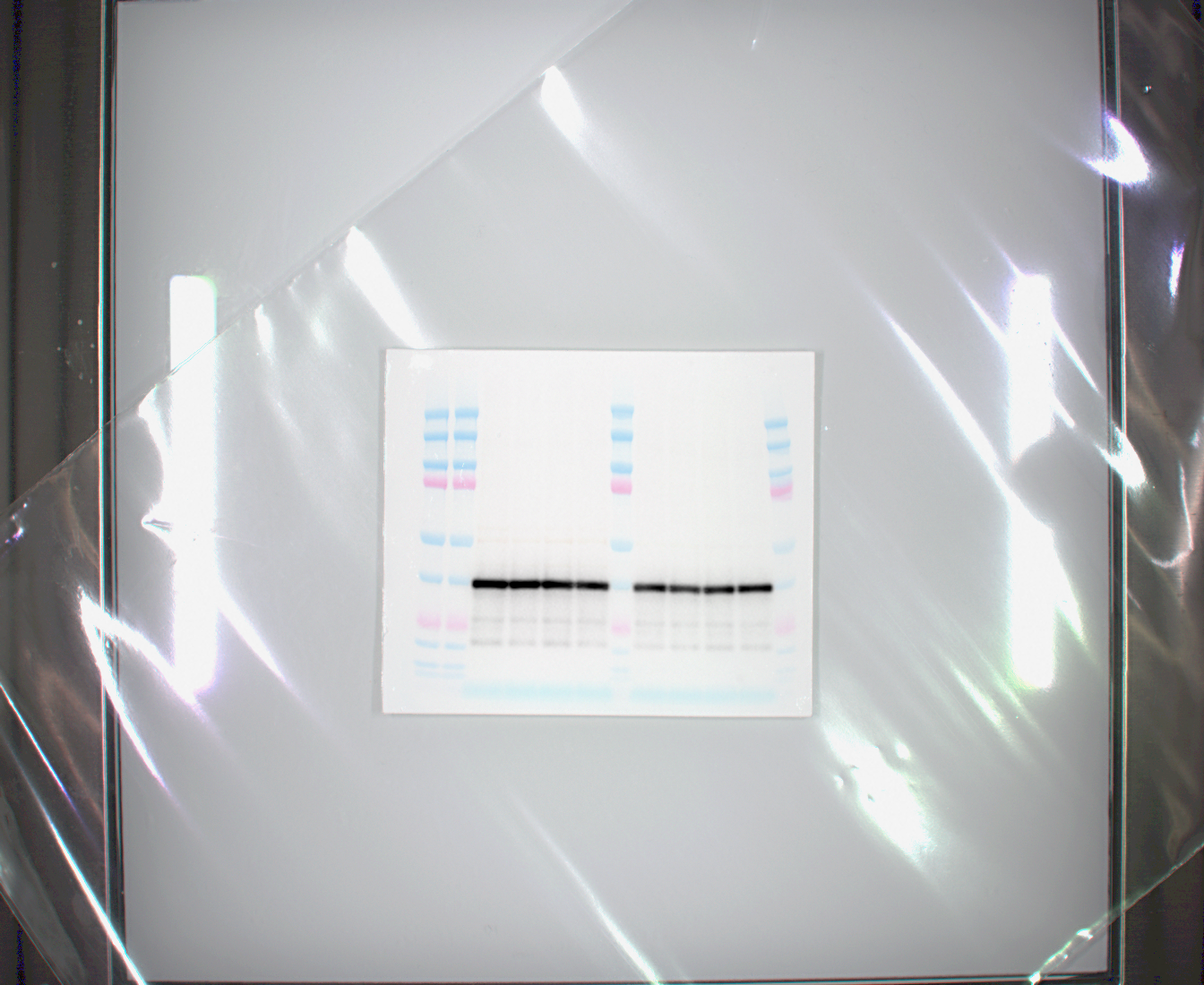

Supplement: Supplementary file 27 — Source data Fig. 5 [file 44320_2026_199_MOESM27_ESM.zip › Figure_5/Figure_5D_GAPDH.Tif]
