# Supplementary material for: Structure-function relationship of alpha-synuclein fibrillar polymorphs derived from distinct synucleinopathies
Source: Mol Syst Biol. 2026 Mar 11;22(6):868–901. doi: 10.1038/s44320-026-00199-5 (PMC13230553; doi:10.1038/s44320-026-00199-5)
Supplement: Supplementary file 28 — Expanded View Figures [file 44320_2026_199_MOESM28_ESM.pdf]

Expanded View Figures

Figure EV1. Characterization of αSyn fibrils PMCA-amplified from PD, MSA, and DLB-patient brains.

Transmission electron microscopy of patient-derived αSyn fibrils (**A**). Scale bar = 200 nm. Limited proteolytic profiles of patient-derived αSyn fibrils (**B**). Digestion of αSyn samples (100 μM monomeric concentration) in the presence of Proteinase K (3.8 μg/ml) was monitored over time at 37 °C and stopped after 0, 1, 5, 15, 60 min digestion by addition of 100 μM PMSF. Limited proteolysis products were dissolved by Hexafluoroisopropanol before denaturation, SDS-PAGE separation and Coomassie staining. Time (min), molecular weight markers (MW, kDa) are shown on the sides and the top of the gels. (**C**, **D**) Thioflavin T fluorescence of amplified strains (**C**) and of brain homogenates used as starting material for amplification. (**D**) The significance of differences between the fibrillar polymorphs derived from PD, DLB, and MSA was assessed using a two-tailed Welch's *t*-test and is indicated as follows: *p val <0.05, **p val <0.01, ***p val <0.001. Exact p values are: $p = 0.0108$ (PD vs MSA), $p = 0.0122$ (PD vs DLB), $p = 0.0093$ (DLB vs MSA) in (**B**), and $p = 0.0108$ (PD vs MSA), $p = 0.0122$ (PD vs DLB), $p = 0.0093$ (DLB vs MSA) and $p = 0.0048$ (PD vs MSA), $p = 0.0008$ (PD vs DLB), $p = 0.0005$ (DLB vs MSA), $p = 0.0032$ (PD vs Control), $p = 0.0008$ (DLB vs Control), and $p = 0.0008$ (MSA vs Control) in (**C**). Source data are available online for this figure.

 © The Author(s)

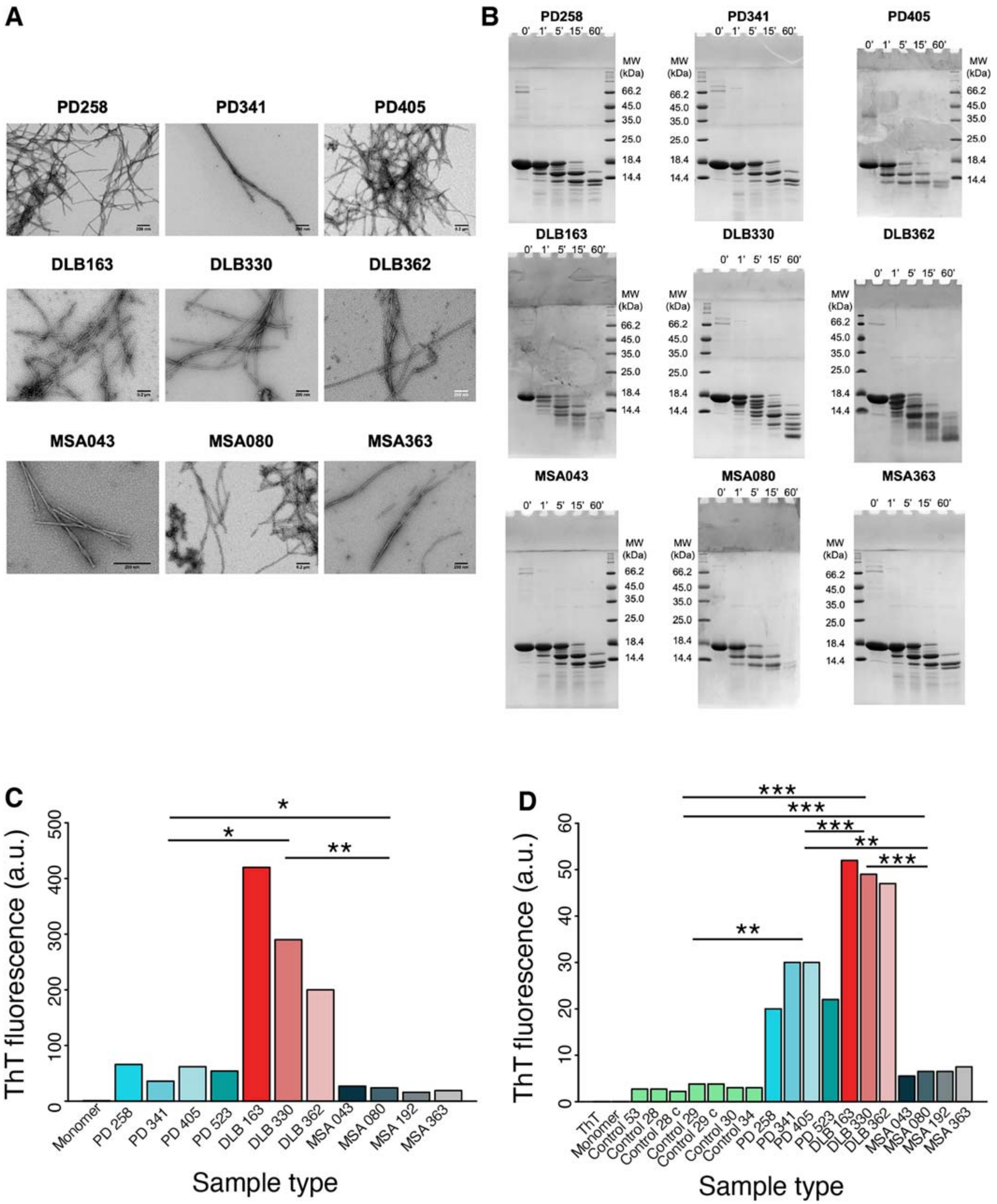
A
PD258
PD341
PD405
DLB163
DLB330
DLB362
MSA043
MSA080
MSA363
B
PD258
PD341
PD405
DLB163
DLB330
DLB362
MSA043
MSA080
MSA363
0' 1' 5' 15' 60'
MW (kDa)
66.2
45.0
35.0
25.0
18.4
14.4
C
ThT fluorescence (a.u.)
Sample type
Monomer
PD 258
PD 341
PD 405
PD 523
DLB 163
DLB 330
DLB 362
MSA 043
MSA 080
MSA 192
MSA 363
D
ThT
Control 53
Control 28
Control 28 c
Control 29
Control 29 c
Control 30
Control 34

© The Author(s)

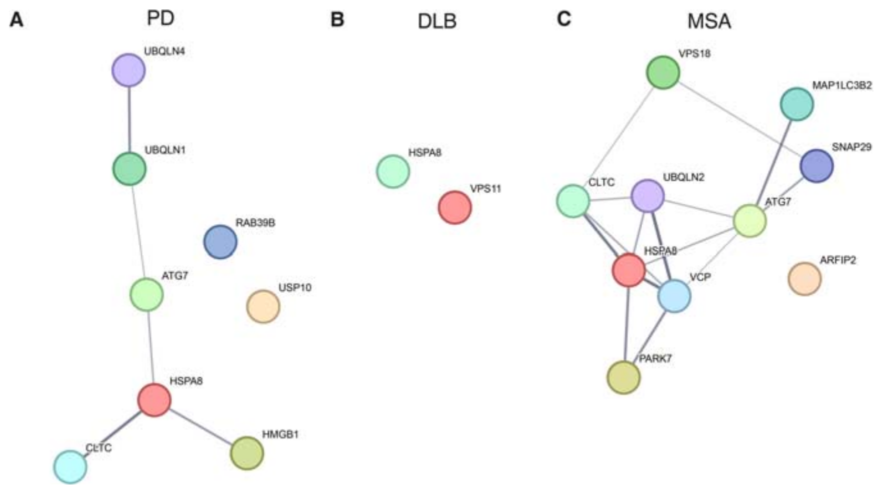

Figure EV2. Autophagy-related proteins respond differentially to αSyn fibrils of the three disease strains.

Hit proteins responding to αSyn fibril uptake (FC >1.5, *q* val <0.05) and involved in autophagy (based on annotation in Uniprot) were analyzed in String to reveal possible protein networks. Resulting plots are shown for PD (**A**), DLB (**B**), and MSA (**C**) strains. Nodes indicate proteins, edges indicate physical or functional interactions based on experiments, databases, and textmining. Line thickness indicates the strength of the data support.

 © The Author(s)
