# Supplementary material for: Structure-function relationship of alpha-synuclein fibrillar polymorphs derived from distinct synucleinopathies
Source: Mol Syst Biol. 2026 Mar 11;22(6):868–901. doi: 10.1038/s44320-026-00199-5 (PMC13230553; doi:10.1038/s44320-026-00199-5)
Supplement: Supplementary file 1 — Peer Review File [file 44320_2026_199_MOESM1_ESM.pdf]

molecular systems biology

Structure-function relationship of alpha-synuclein fibrils derived from distinct synucleinopathies

Tetiana Serdiuk, Virginie Redeker, Jimmy Savistchenko, Sandesh Neupane, Walther Haenseler, Yanick Fleischmann, Viviane Reber, Sabrina Keller, Cinzia Tiberi, Ruxandra Bachmann-Gagescu, Matthias Gstaiger, Thomas Braun, Roland Riek, Steve Gentleman, Adriano Aguzzi, Natalie de Souza, Ronald Melki, and Paola Picotti

Corresponding author(s): Paola Picotti (picotti@imsb.biol.ethz.ch) , Ronald Melki (ronald.melki@cnrs.fr)

Review Timeline:

Submission Date:	19th May 25
Editorial Decision:	23rd Jun 25
Revision Received:	13th Nov 25
Editorial Decision:	22nd Dec 25
Revision Received:	31st Jan 26
Accepted:	9th Feb 26

Editor: Jingyi Hou

Transaction Report:

(Note: With the exception of the correction of typographical or spelling errors that could be a source of ambiguity, letters and reports are not edited. Depending on transfer agreements, referee reports obtained elsewhere may or may not be included in this compilation. Referee reports are anonymous unless the Referee chooses to sign their reports.)

23rd Jun 2025

Manuscript Number: MSB-2025-13124-T
Title: Structure-function relationship of alpha-synuclein fibrils derived from distinct synucleinopathies
Author: Tetiana Serdiuk
Virginie Redeker
Jimmy Savistchenko
Sandesh Neupane
Walther Haenseler
Yanick Fleischmann
Sabrina Keller
Cinzia Tiberi
Ruxandra Bachmann-Gagescu
Matthias Gstaiger
Thomas Braun
Roland Riek
Steve Gentleman
Adriano Aguzzi
Natalie de Souza
Ronald Melki
Paola Picotti

Dear Dr. Picotti,

Thank you for submitting your work to Molecular Systems Biology. We have now heard back from the three reviewers who agreed to evaluate your manuscript. As you will see from the reports below, the reviewers acknowledge the interest of the study. They raise, however, a series of concerns, which we would ask you to address in a major revision.

While Reviewers #1 and #3 are generally supportive, Reviewer #2 raised concerns about the validation of the findings and whether the PMCA fibrils possess disease-relevant structures. Reviewer #2 also provided constructive suggestions for addressing these issues. These concerns should be carefully considered to strengthen the disease relevance of the study and enhance the conclusiveness of the findings.

All other issues need to be addressed as well. As you may already know, our editorial policy allows in principle a single round of major revision, and it is therefore essential to provide responses to the reviewers' comments that are as complete as possible. Please feel free to contact me in case you would like to discuss in further detail any of the issues raised by the reviewers.

On a more editorial level, we would ask you to address the following issues:

- Please provide a .docx formatted version of the manuscript text (including legends for main figures, EV figures and tables). Please make sure that the changes are highlighted to be clearly visible.

- Please provide individual production quality figure files as .eps, .tif, .jpg (one file per figure).

-Please provide a .docx formatted letter INCLUDING the reviewers' reports and your detailed point-by-point responses to their comments. As part of the EMBO Press transparent editorial process, the point-by-point response is part of the Review Process File (RPF), which will be published alongside your paper.

-Please note that all corresponding authors are required to supply an ORCID ID for their name upon submission of a revised manuscript.

-We replaced Supplementary Information with Expanded View (EV) Figures and Tables that are collapsible/expandable online (see examples in http://msb.embopress.org/content/11/6/812). A maximum of 5 EV Figures can be typeset. EV Figures should be cited as 'Figure EV1, Figure EV2" etc... in the text and their respective legends should be included in the main text after the legends of regular figures.

Additional Tables/Datasets should be labeled and referred to as Table EV1, Dataset EV1, etc. Legends have to be provided in a separate tab in case of .xls files. Alternatively, the legend can be supplied as a separate text file (README) and zipped together with the Table/Dataset file.
For the figures and tables that you do NOT wish to display as Expanded View figures, they should be bundled together with their legends in a single PDF file called *Appendix*, which should start with a short Table of Content. Each legend should be below

the corresponding Figure/Table in the Appendix. Appendix figures and tables should be referred to in the main text as: "Appendix Figure S1, Appendix Figure S2, Appendix Table S1" etc. See detailed instructions regarding expanded view here: https://www.embopress.org/page/journal/17444292/authorguide#expandedview.

-Before submitting your revision, primary datasets (and computer code, where appropriate) produced in this study need to be deposited in an appropriate public database (seehttp://msb.embopress.org/authorguide - dataavailability https://www.embopress.org/page/journal/17444292/authorguide#dataavailability).
Please remember to provide a reviewer password if the datasets are not yet public.
The accession numbers and database should be listed in a formal "Data Availability" section (placed after Materials & Method) that follows the model below (see also https://www.embopress.org/page/journal/17444292/authorguide#dataavailability). Please note that the Data Availability Section is restricted to new primary data that are part of this study.
Data availability
The datasets (and computer code) produced in this study are available in the following databases:
- RNA-Seq data: Gene Expression Omnibus GSE46843 (https://www.ncbi.nlm.nih.gov/geo/query/acc.cgi?acc=GSE46843)
- [data type]: [name of the resource] [accession number/identifier/doi] ([URL or identifiers.org/DATABASE:ACCESSION])
*** Note - All links should resolve to a page where the data can be accessed. ***

-At EMBO Press we ask authors to provide source data for the main figures. Our source data coordinator will contact you to discuss which figure panels we would need source data for and will also provide you with helpful tips on how to upload and organize the files.

- Our journal encourages inclusion of *data citations in the reference list* to directly cite datasets that were re-used and obtained from public databases. Data citations in the article text are distinct from normal bibliographical citations and should directly link to the database records from which the data can be accessed. In the main text, data citations are formatted as follows: "Data ref: Smith et al, 2001". In the Reference list, data citations must be labeled with "[DATASET]". A data reference must provide the database name, accession number/identifiers and a resolvable link to the landing page from which the data can be accessed at the end of the reference. Further instructions are available at .

- We updated our journal's competing interests policy in January 2022 and request authors to consider both actual and perceived competing interests. Please review the policy https://www.embopress.org/competing-interests and update your competing interests if necessary.
Please use the heading "Disclosure statement and competing interests".

- All Materials and Methods need to be described in the main text using our 'Structured Methods' format. According to this format, the Methods section includes a Reagents and Tools Table (listing key reagents, experimental models, software and relevant equipment and including their sources and relevant identifiers) followed by a Methods and Protocols section describing the methods, ideally using a step-by-step protocol format. The aim is to facilitate adoption of the methodologies across labs. Please download and fill our Reagents and Tools Table template (.docx), which you can find in our author guidelines: https://www.embopress.org/page/journal/17444292/authorguide#structuredmethods.
When submitting your revised manuscript, please do not include the Reagents and Tools Table in the Methods section of the manuscript but upload it as a separate file choosing the file type "Reagent Table".

An example of a Method paper with Structured Methods can be found here: https://www.embopress.org/doi/10.15252/msb.20178071.

-Regarding data quantification:
Please ensure to specify the name of the statistical test used to generate error bars and P values, the number (n) of independent experiments (please specify technical or biological replicates) underlying each data point and the test used to calculate p-values in each figure legend. Discussion of statistical methodology can be reported in the materials and methods section, but figure legends should contain a basic description of n, P and the test applied.
Graphs must include a description of the bars and the error bars (s.d., s.e.m.).
Please also include scale bars in all microscopy images.

- Please provide a "standfirst text" summarizing the study in one or two sentences (approximately 250 characters, including space), three to four "bullet points" highlighting the main findings and a "synopsis image" (550px width and 400-600 px height, PNG format) to highlight the paper on our homepage.
Here are a couple of examples:
https://www.embopress.org/doi/10.15252/msb.20199356
https://www.embopress.org/doi/10.15252/msb.20209475
https://www.embopress.org/doi/10.15252/msb.209495

When you resubmit your manuscript, please download our CHECKLIST (https://www.embopress.org/pb-assets/embo-site/EMBO%20Press%20Author%20Checklist-1642513524327.xlsx) and include the completed form in your submission. *Please note* that the Author Checklist will be published alongside the paper as part of the transparent process

(https://www.embopress.org/page/journal/17444292/authorguide#transparentprocess).

If you feel you can satisfactorily deal with these points and those listed by the referees, you may wish to submit a revised version of your manuscript. Please attach a covering letter giving details of the way in which you have handled each of the points raised by the referees. A revised manuscript will be once again subject to review and you probably understand that we can give you no guarantee at this stage that the eventual outcome will be favorable.

I look forward to receiving your revised manuscript soon.

Kind regards,
Jingyi

Jingyi Hou, PhD
Senior Editor
Molecular Systems Biology

We realize that it is difficult to revise to a specific deadline. In the interest of protecting the conceptual advance provided by the work, we recommend a revision within 3 months (21st Sep 2025). Please discuss the revision progress ahead of this time with the editor if you require more time to complete the revisions. Use the link below to submit your revision:

IMPORTANT: When you send your revision, we will require the following items:

1. the manuscript text in LaTeX, RTF or MS Word format
2. a letter with a detailed description of the changes made in response to the referees. Please specify clearly the exact places in the text (pages and paragraphs) where each change has been made in response to each specific comment given
3. three to four 'bullet points' highlighting the main findings of your study
4. a short 'blurb' text summarizing in two sentences the study (max. 250 characters)
5. a 'thumbnail image' (550px width and max 400px height, Illustrator, PowerPoint or jpeg format), which can be used as 'visual title' for the synopsis section of your paper.
6. Please include an author contributions statement after the Acknowledgements section (see https://www.embopress.org/page/journal/17444292/authorguide)
7. Please complete the CHECKLIST available at (https://bit.ly/EMBOPressAuthorChecklist).
Please note that the Author Checklist will be published alongside the paper as part of the transparent process (https://www.embopress.org/page/journal/17444292/authorguide#transparentprocess).
8. When assembling figures, please refer to our figure preparation guideline in order to ensure proper formatting and readability in print as well as on screen:
https://bit.ly/EMBOPressFigurePreparationGuideline
See also figure legend guidelines: https://www.embopress.org/page/journal/17444292/authorguide#figureformat
9. Please note that corresponding authors are required to supply an ORCID ID for their name upon submission of a revised manuscript (EMBO Press signed a joint statement to encourage ORCID adoption).
(https://www.embopress.org/page/journal/17444292/authorguide#editorialprocess)
Currently, our records indicate that the ORCID for your account is 0000-0002-4109-3552.

Please click the link below to modify this ORCID:
Link Not Available

10. At EMBO Press we ask authors to provide source data for the main manuscript figures. You will receive a separate email with instructions for providing source data with your revised manuscript, including how to upload and organize the files.

11. Include a Reagents and Tools Table, which can be downloaded from our author guidelines (https://www.embopress.org/page/journal/17444292/authorguide#structuredmethods)

As a matter of course, please make sure that you have correctly followed the instructions for authors as given on the submission website.

*** PLEASE NOTE *** As part of the EMBO Press transparent editorial process initiative (see our Editorial at https://dx.doi.org/10.1038/msb.2010.72), Molecular Systems Biology publishes online a Review Process File with each accepted

manuscripts. This file will be published in conjunction with your paper and will include the anonymous referee reports, your point-by-point response and all pertinent correspondence relating to the manuscript. If you do NOT want this File to be published, please inform the editorial office at contact@molsystbiol.org within 14 days upon receipt of the present letter.

Reviewer #1:

This manuscript presents a comprehensive proteomic study characterizing structural and functional differences among α-synuclein (αSyn) fibrillar polymorphs derived from patients with Parkinson's disease (PD), dementia with Lewy bodies (DLB), and multiple system atrophy (MSA). In particular, the authors-recognized international experts in LiP-MS-apply their methods to investigate the structure-function relationship of αSyn in both in vitro and cellular contexts, including SH-SY5Y cells, iPSC-derived neurons, and patient brain homogenates. Overall, while the study remains preliminary for disease mechanistic biology, it represents a large-scale effort with methodological innovations, rich datasets, and interesting biological insights and discoveries. We therefore think it is a strong candidate for publication in MSB following revision.

Major:
1. Mechanistic Gap in DLB-Associated αSyn Clearance Defect:
One of the most striking findings seems to be the pronounced accumulation of DLB-derived αSyn fibrils across models, suggesting suppressed clearance (Figures 2C, 5C, 6E). However, the mechanistic basis for this defect remains unclear to us after reading through the paper. The CRISPR activation experiments show that overexpression of UPS regulators such as TRIM25, UBE3A, HUWE1, and VCP reduces αSyn inclusions in PD and MSA, but has (even) less effect in the DLB context. This may suggest that UPS engagement is relatively less important in DLB overall, or that DLB fibrils evade or impair degradation? We view this as a major interpretive gap that warrants further discussion, or ideally, experimental follow-up. Is the reduced clearance in DLB due to structural masking, impaired recognition, altered trafficking, or proteasome inhibition? We encourage the authors to address this through either new experiments, or a strengthened discussion, or proposed experimental directions (e.g., subcellular localization, UPS activity assays, etc.). What do the authors think is really happening?

2. Loss-of-Function Data: The authors exclusively use CRISPRa-based gain-of-function approaches to show that certain UPS regulators reduce αSyn inclusions. However, complementary loss-of-function experiments (e.g., CRISPRi or RNAi knockdown of TRIM25, HUWE1, or VCP) would better clarify whether these factors are necessary for αSyn turnover, particularly in the PD and MSA conditions. At a minimum, some discussion of this limitation would be appreciated.

3. αSyn Interactome Data: The strain-specific αSyn interactome reported here is very interesting. Is there a way to enhance this aspect, e.g., by incorporating or referencing existing αSyn interactome datasets or AP-MS datasets, if available?

Minor:
1. In the Methods, both TEM and LiP-MS are performed after 20 minutes of sonication. Was covalent painting also performed after sonication? If so, how do the authors ensure that such intense treatment does not alter the conformational states of disease-derived αSyn fibrils?
2. Line 146: ThT Fluorescence Interpretation:
Please explain what the ThT fluorescence signal differences imply in terms of fibril structure, and whether they are consistent with the LiP-MS findings.
3. Figure 1A: Covalent Painting Illustration:
The schematic for covalent painting (red peptides) could be improved to match the clarity and informativeness of the LiP-MS illustration.
4. Line 197: Agreement Between LiP-MS and Painting Results:
The authors state that LiP-MS results for the NAC and pre-NAC regions agree with covalent painting. However, K80 appears least accessible in DLB rather than MSA. Please clarify this point or reconcile the discrepancy.
5. Complementarity of LiP-MS and Covalent Painting: While both methods are informative, the correlation between their results appears limited to us. The authors may consider further emphasizing their "complementarity" in terms of structural resolution and coverage.
6. Line 282 and 325: These sections appear to describe results from the same experiment? Please clarify.
7. Line 864: The final statement regarding the number of altered proteins for drug development seems exaggerated. As compared to the total proteome?
8. PMCA Clarification: Although PMCA-based amplification has been published previously, it would be helpful to briefly describe its workflow and clarify again its fidelity. Additionally, it remains unclear to us how the patient-derived αSyn were sufficient for the large number of experiments presented? Were all fibrils used PMCA-amplified? Please clarify.
9. Figure 2E: The concept of relative ubiquitination as presented is still not fully clear. The authors should improve the explanation of how this was measured and interpreted.
10. Line 1107: Please cite the version and reference of Spectronaut and clarify whether GlyGly(K) modifications were searched across the entire proteome or targeted to αSyn only. Genearlly, please improve the description here.
11. Line 976: it is interesting to think about how much αSyn fibril was spiked into the various systems? Is the concentration used (e.g., 250 nM) based on physiological estimates, or chosen for practical detection reasons?

Reviewer #2:

In this manuscript, Serdiuk et al. generate amyloid fibrils of recombinant alpha-synuclein (asyn) using protein misfolding cyclic amplification (PMCA) with PD, DLB and MSA brain homogenates. The authors characterise the resulting PMCA fibrils using TEM, limited proteolysis coupled to mass spectrometry (LiP-MS), ThT fluorescence and lysine accessibility (covalent molecular painting), showing that PMCA fibrils generated with different brain homogenates (PD, DLB and MSA) have distinct structural properties. The authors then perform proteome-wide LiP-MS on PMCA fibrils incubated with SH-SY5Y cells and lysates, and iPSC-derived cortical neurons. This supports the conclusion that the PMCA fibrils generated with different brain homogenates (PD, DLB and MSA) have distinct structural properties and suggests that they also have distinct interactomes. The authors then perform LiP-MS directly on PD, DLB and MSA brain homogenates, which matches a subset of the interactomes identified in the cell experiments. Finally, the authors over-express candidate protein degradation-related interactors identified by LiP-MS in HEK293 cells over-expressing asyn and incubated with PMCA fibrils. This results in reductions in pS129 asyn-positive cells in a fibril structure-dependent manner, consistent with the interactions predicted by LiP-MS.

Overall, this work is conducted to a high technical standard, combining the complementary expertise of the co-senior authors' groups in LiP-MS and amyloid fibril biology. The finding that alpha-synuclein fibrils with different structural properties have distinct interactomes with functional relevance to their degradation is conceptually important, as it supports the hypothesis that fibrils with distinct structures act as 'strains' to give rise to different diseases. The main caveat of this work is that it is not possible to assess the disease-relevance of many of the specific interactions identified, because it is not clear if the PMCA fibrils have disease-relevant structures. More detail on this and other, more minor, comments is provided below.

Major comments:
1. It is not clear if the PMCA method used to generate fibrils for this study produces disease-relevant structures. The structures of the ordered cores of fibrils from PD, DLB and MSA brain have been determined using cryo-EM (Schweighauser et al. 2020. Nature 585, 464-469; Yang et al. 2022. Nature 610, 791-795). However, the structure of the ordered core of fibrils generated by seeding recombinant alpha-synuclein with fibrils from MSA brain was different (Lövestam et al. 2021. FEBS Open Bio 11, 999-1013), showing that such 'seeding' methods do not necessarily reproduce disease-relevant structures. The authors should compare the structures of the ordered cores of their PMCA fibrils to the published structures of the ordered cores of fibrils in PD, DLB and MSA. Ideally, this would be achieved by determining the structures using cryo-EM or NMR. If this is not possible, the authors could assess if their TEM analysis (projected widths, helical crossover distances), covalent molecular painting (exposed lysine residues) and LiP-MS analysis (protease-accessible sites) are consistent with the published structures. In the absence of such analysis, it is also not clear how reliable the authors' structural findings relating to regions outside of the ordered fibril cores are.

2. There is a general lack of validation of candidate fibril interactors identified by LiP-MS. In Fig. 3, the authors validate the interaction of UCHL1 (identified in LiP-MS of PMCA fibrils incubated with SH-SY5Y cell lysates) with alpha-synuclein monomers and all PMCA fibrils. It would be beneficial to also validate candidate fibril interactions identified by LiP-MS of PMCA fibrils incubated with SH-SY5Y cells and iPSC-derived cortical neurons, as well as of the human brain homogenates. In additiona, it would be helpful to validate candidate interactors that are specific to each of the alpha-synuclein species (monomer and PD/ DLB/ MSA homogenate-derived PMCA fibrils). This would also help to confirm that there are alpha-synuclein species-specific interactors and that this is not instead dependent on the sensitivity of LiP-MS.

Minor comments:
1. Are differences in ubiquitination of PMCA fibrils in SH-SY5Y lysate due to differences in lysine availability measured by covalent molecular painting?
2. Line 353: Should 'are' be 'were?'
3. Line 370: Appears to be repetition of line 367 (referencing stability and ubiquitination)
4. Presumably the lysis of the SH-SY5Y cells and iPSC-derived neurons prior to LiP-MS will contribute artificial interactions? Do the authors have any way to control for this?
5. Line 698: is Why asyn overexpression used here and not in previous cell experiments? Does this lead to any spontaneous fibril formation that could confound the effects of the PMCA fibrils? Do the authors observe seeded aggregation of the over-expressed asyn (i.e. is it the seeds or seeded aggregates that become phosphorylated at S129)?
6. Fig 8C: The n number not given and error bars are not present. Are two-tailed t-tests suitable, since multiple conditions are being compared to a control?

Reviewer #3:

Summary
In their manuscript, Serdiuk et al study alpha-Synuclein aggregation and its effects in different synucleinopathies, Parkinson's Disease (PD), Dementia with Lewy Bodies (DLB) and Multiple System Atrophy (MSA). They make use of different MS-based techniques to study aggregation patterns in the different disease phenotypes and identify proteins interacting with and affected

by each specific synucleinopathy. They first investigate stability and conformational space for each PD, DLB, and MSA patient-derived αSyn polymorphs in patient brain lysates. The authors then analyze the stability of different aSyn polymorphs in a neuroblastoma cell lysate, and study the respective interactomes and structural proteomic effects upon exposure. They identify both known and novel interaction partners for each synucleinopathy, including E3 ligases such as HUWE1, DUBs such as UCHL1 and chaperones such as VCP. The authors then examine in aSyn-expressing HEK293 cells if overexpression of five of these candidate proteins affects clearance of aSyn fibrils after exposure to fibrillary polymorphs from the different synucleinopathies.
Taken together, the authors gain new insights into the structural dynamics of aSyn in different neurodegenerative diseases and identify potential targets for diagnostics and therapy. They make use of sensitive LC-MS-based approaches (LiP-MS and molecular painting) and validate and complement these findings biochemically and via NMR, working either directly with patient-derived samples or in cell culture model systems.

General remarks
The authors present a consistent and convincing story. The data are solid, and the conclusions are valid. Overall this manuscript provides important new data for a-Syn fibril structures and interaction partners in different neurobiological diseases, providing new insights that may have clinical relevance. aSyn fibrils are notoriously difficult to study in situ in their cellular environment, and structural proteomics methods such as LiP-MS offer a unique advantage to monitor both the target protein (aSyn) as well as other, affected proteins in different synucleinopathies. As such, this manuscript will be of interest to a broad audience of neurobiologists and I fully support publication in Mol Sys Biol once the points listed below have been addressed:

Major points
Overall this is comprehensive and nice piece of work. I only have a few points that need to be addressed, and some additional suggestions most likely for future studies.

- The authors use brain homogenates from cingulate cortex for PD and DLB, but cerebellum for MSA. Are there known differences in aSyn aggregates in different areas of the brain? What are the difference in aSyn for MSA in cingulate cortex, and for PD and DLB in the cerebellum? If no differences are expected this would serve as a good internal sanity check for physiological relevance.

- The term "mitochondria" comes up in multiple experiments. In what way are mitochondria affected upon aSyn treatment of the cells - are they still "ok" morphologically, are they undergoing mitophagy or are there any other effects detectable using immunofluorescence or other orthogonal approaches?

- Figure 2: The data shown in panels B and C appear inconsistent with panel D: The Western blot in panel D PD displays the strongest band for aSyn, whereas DLB shows the highest aSyn abundance in panel C. If this discrepancy derives from the loaded protein amounts in D (a-tub input control is higher too though), then it may make sense to show a normalized box plot with error bars

- The authors show several affected interfaces on target proteins such as Rab13, DDC, CFAP298, VATB2, UCHL1 and VCP upon cellular aSyn exposure (Fig.3/5/6/7). While the primary amino acid sequence of these proteins may be quite different, did the authors examine if there are any conserved structural motifs or domains present in these proteins? A simple alignment is probably not sufficient, but a comparison of the respective domains may yield interesting insights.

Optional points (involving significant amounts of work, not mandatory for this reviewer but food for thought for future studies:

- The authors discuss protein stability extensively. The way to systematically study aSyn fibril stability - and its effect on other, associated proteins - would be to conduct a pulsed SILAC experiment and determine aSyn and proteome-wide protein synthesis and degradation rates. This would be straightfoward in the SH-SY5Y cells but would still be a substantial amount of work, and be particularly challenging for short time points.

- There is a section on sarkosyl precipitation of aSyn fibrils: It would make sense to compare aSyn from sarkosyl precipitations from the brain to fibrils amplified directly from native patient brain homogenates using LiP-MS, to examine and compare the two model systems systematically.

Minor points
- Section 459-482 needs to be clarified a bit (the section on mitochondria is a bit vague, see above; the paragraph reads more like a concluding remark rather than the start of a section)

Molecular Systems Biology, MSB-2025-13124-T

Structure-function relationship of alpha-synuclein fibrils derived from distinct synucleinopathies

Response to all Referees and Editor

We thank all the reviewers for recognizing the novelty and scientific impact of our study, as well as for their constructive comments, which have helped us to further improve and strengthen the manuscript. We have now performed additional experiments and analyses to address their critique and strengthen the manuscript. In particular, we have:

- Provided additional evidence supporting amplification fidelity.
- Performed new experiments to validate strain-specific interactors.
- Expanded the discussion of our results related to strain degradation, the effects of fibrils on mitochondria, and future experimental directions.
- Addressed several technical and methodological points.

A detailed, point-by-point response follows below.

Point-by-point response to the reviewers

Reviewer #1:

This manuscript presents a comprehensive proteomic study characterizing structural and functional differences among α-synuclein (αSyn) fibrillar polymorphs derived from patients with Parkinson's disease (PD), dementia with Lewy bodies (DLB), and multiple system atrophy (MSA). In particular, the authors-recognized international experts in LiP-MS-apply their methods to investigate the structure-function relationship of αSyn in both in vitro and cellular contexts, including SH-SY5Y cells, iPSC-derived neurons, and patient brain homogenates. Overall, while the study remains preliminary for disease mechanistic biology, it represents a large-scale effort with methodological innovations, rich datasets, and interesting biological insights and discoveries. We therefore think it is a strong candidate for publication in MSB following revision.

We thank the reviewer for their encouraging comments.
Major:
1. Mechanistic Gap in DLB-Associated αSyn Clearance Defect:
One of the most striking findings seems to be the pronounced accumulation of DLB-derived αSyn fibrils across models, suggesting suppressed clearance (Figures 2C, 5C, 6E). However, the mechanistic basis for this defect remains unclear to us after reading through the paper. The CRISPR activation experiments show that overexpression of UPS regulators such as TRIM25, UBE3A, HUWE1, and VCP reduces αSyn inclusions in PD and MSA, but has (even) less effect in the DLB context. This may suggest that UPS engagement is relatively less important in DLB overall, or that DLB fibrils evade or impair degradation? We view this as a major interpretive gap that warrants further discussion, or ideally, experimental follow-up. Is the reduced clearance in DLB due to structural masking, impaired recognition, altered trafficking, or proteasome inhibition? We encourage the authors to address this through either new experiments, or a strengthened discussion, or proposed experimental directions (e.g., subcellular localization, UPS activity assays, etc.). What do the authors think is really happening?

We thank the reviewer for their question. We also find the DLB-associated defect in αSyn clearance very interesting. In our view, addressing this effect experimentally falls beyond the

scope of the current manuscript and will be pursued in a dedicated follow-up study. As suggested, we have included a discussion of possible mechanisms underlying this effect, along with future experimental directions (lines 835-852):

"The observed DLB-associated defect of αSyn clearance provides further strong evidence for the importance of fibrillar shape and exposed surfaces in determining disease phenotype. Across multiple experimental systems (SH-SY5Y lysate, SH-SY5Y living cells and iPSCs-derived cortical neurons), we have observed significantly higher relative accumulation of DLB as compared to PD and MSA fibrils. Consistent with this, the response of E3 ubiquitin ligases and UPS in general was lower for the DLB polymorph, based on both LiP-MS and CRISPR results (Figures 5 and 8). This effect could be due to different scenarios: (i) masking of the DLB strain from the UPS system by other interactors (proteins or lipid membranes), (ii) buried localization of amino-acids critical for recognition and targeting for degradation in the structure of DLB fibrils (including specific post-translational modifications), (iii) blockage of the proteasome by the DLB strain, (IV) disease-specific differences in cellular localization of the fibrils. To understand this effect more in detail, follow up studies will be necessary. These should involve assessment of the sub-cellular localization of DLB strain using high-resolution fluorescence microscopy techniques and investigation of the effects of different inhibitors (e.g., proteasomal inhibitors, autophagy and lysosomal inhibitors). Furthermore, SILAC pulse-chase experiments on neurons seeded with heavy-labelled disease-specific fibrils could shed light on the dynamics of disease-specific fibril degradation and its interplay with endogenous αSyn seeding."

2. Loss-of-Function Data: The authors exclusively use CRISPRa-based gain-of-function approaches to show that certain UPS regulators reduce αSyn inclusions. However, complementary loss-of-function experiments (e.g., CRISPRi or RNAi knockdown of TRIM25, HUWE1, or VCP) would better clarify whether these factors are necessary for αSyn turnover, particularly in the PD and MSA conditions. At a minimum, some discussion of this limitation would be appreciated.

We thank the reviewer for raising this point. We chose to study activation rather than loss-of-function because E3 ubiquitin ligases and chaperones (like VCP) often have broad and redundant functions, which can complicate the interpretation of gene silencing experiments. By focusing on activation, we aimed to identify specific gain-of-function effects that might be masked or compensated in models based on silencing. However, we agree that loss-of-function studies would be complementary and may provide valuable insight and have included a discussion on these limitations in the Discussion section of our manuscript (lines 747-750):

"While CRISPR activation experiments have shown the strain-specific effect of TRIM25, UBE3A, HUWE1 and VCP on pSer129 aggregates, the corresponding loss-of-function experiments will be important in the future to define whether these modulators are required for αSyn strain-specific turnover."

3. αSyn Interactome Data: The strain-specific αSyn interactome reported here is very interesting. Is there a way to enhance this aspect, e.g., by incorporating or referencing existing αSyn interactome datasets or AP-MS datasets, if available?

We thank the reviewer for their suggestion. As recommended, we have now compared our LiP-MS-based disease-specific αSyn candidate interactomes (which we expect to include both direct and indirect interactors) with previously reported disease-specific αSyn interactomes based on a different method. Specifically, we compared our hits to a dataset generated by an interesting in situ proximity-labelling approach called biotinylation by antibody recognition (BAR-MS); this was applied to identify the protein "proxisome" of αSyn in brain lysates from PD/DLB and MSA[1] patients, e.g proteins within at least a 14 nm radius. We have found that among 245 proteins identified by BAR-MS (using MJFR1 antibodies) for PD/DLB, 62 were also identified in our LIP-MS experiments (Figure 3, Table EV15). For MSA, 25 out of 175 proteins

reported by BAR-MS were also detected by LiP-MS (Table EV15). We have added a comment on this to the manuscript discussion (lines 898-815):

"Applying LiP-MS to lysates treated with αSyn polymorphs revealed disease-specific structural changes of proteins including potential disease-specific interactors of αSyn fibrils. A recent study used an orthogonal in situ proximity-labeling approach (BAR-MS) to characterize proteins in the vicinity of pathogenic αSyn in PD/DLB and MSA human brain homogenates[1]. BAR-MS employed either anti-pS129 or anti–total αSyn antibodies. We compared our LiP-MS results with the anti–total αSyn dataset, as pS129-positive αSyn was not detected in our setup, likely due to short lysate exposure (15 min) and low endogenous αSyn levels in SH-SY5Y lysates critical for pS129 inclusion formation.

For PD/DLB, of the 245 proteins reported to localize in close proximity to pathogenic αSyn by BAR-MS, 62 were identified as LiP-MS hits (Table EV15). For MSA, 25 of the 175 proteins identified by BAR-MS were also identified by LiP-MS. Importantly, BAR-MS identifies proteins in proximity (within at least a 14 nm radius) to aggregated αSyn, not necessarily in direct contact with the target protein. Furthermore, BAR-MS and LiP-MS were performed on different sample types (patient brain homogenates as compared to SH-SY5Y lysates used in our study) and brain regions (forebrain/midbrain as compared to cerebellum for MSA and cingulate gyrus for PD/DLB in our study). This likely contributes to the differences we highlight. Notably, LiP-MS also provides potential interaction sites for proteins found by both studies, which could be considered high-confidence hits to prioritize in future functional studies (Table EV15)."

Minor:
1. In the Methods, both TEM and LiP-MS are performed after 20 minutes of sonication. Was covalent painting also performed after sonication? If so, how do the authors ensure that such intense treatment does not alter the conformational states of disease-derived αSyn fibrils?

We thank the reviewer for the question. Covalent painting was performed on unfragmented fibrils. We clarified this issue in the methods section of the manuscript.
We have tested the effect of sonication and have seen that sonication does not change the PK digestion fingerprint by SDS-PAGE fingerprinting[2,3] . Further, multiple studies have shown that fibril sonication can break the amyloid fibrils into smaller fragments (on the fibril elongation axis), but that their structure is preserved[4-7]).

The following text has been added to the methods (lines 989-990):

"In contrast to LiP-MS, covalent surface painting has been performed on unfragmented fibrils."

2. Line 146: ThT Fluorescence Interpretation:
Please explain what the ThT fluorescence signal differences imply in terms of fibril structure, and whether they are consistent with the LiP-MS findings.

We used ThT fluorescence in this study as an orthogonal approach to evaluate structural differences between fibrils. Previous work, including ours, has shown that ThT can reliably distinguish fibrils amplified from patients with distinct synucleinopathies, as well as de novo–generated fibrillar strains[8-10]. Recent structural studies revealed that ThT interacts not only with the amyloid core but also with the N-terminal region of αSyn fibrils, forming a binding pocket that involves both NAC and N-terminal residues[11].

In our molecular painting results, DLB fibrils have the most accessible lysine residues in the N-terminal region, while MSA fibrils show the lowest solvent accessibility in this region. This suggests that the N-terminal region is generally more exposed in DLB fibrils than in MSA fibrils, which may affect ThT binding. The higher ThT signal observed for DLB fibrils compared with MSA fibrils likely reflects this greater surface accessibility of N-terminal residues,

consistent with molecular painting results. In contrast, LiP-MS probes also backbone flexibility, so it is less straightforward to predict how differences in fibril rigidity would affect ThT binding. Overall, our ThT data on both amplified fibrils and brain homogenates recapitulate previous findings showing lower ThT emission for MSA fibrils compared to PD fibrils in human CSF[9] and mouse brain[12]. This is consistent with our LiP-MS and molecular painting results, which show overall higher protection in MSA fibrils, in both the NAC and the N-terminal regions (Fig 1B and D), further supporting that our amplification procedure preserves disease-specific structural features.

We added the following text to the manuscript (lines 148-153):

"ThT has been recently shown to bind a pocket that includes residues from both the NAC and N-terminal regions of αSyn[11]. Differences in ThT fluorescence observed here likely reflect differences in surface accessibility of these residues. Indeed, molecular painting results suggest that N-terminal residues are most accessible in DLB fibrils and least accessible in MSA fibrils. Furthermore, LIP-MS results suggest that the NAC region is less accessible in MSA fibrils."

3. Figure 1A: Covalent Painting Illustration:
The schematic for covalent painting (red peptides) could be improved to match the clarity and informativeness of the LiP-MS illustration.

We have improved the schematics for covalent painting (Revised Figure 1A).

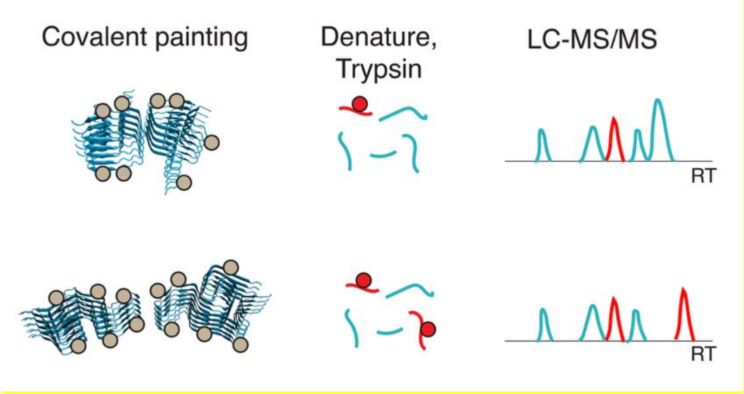

Figure R1 (Revised Figure 1A). Schematic of covalent painting experiment. First, protein surfaces are covalently painted by NHS-biotin. Subsequently, samples are denatured and digested by trypsin for bottom-up MS analysis.

4. Line 197: Agreement Between LiP-MS and Painting Results:
The authors state that LiP-MS results for the NAC and pre-NAC regions agree with covalent painting. However, K80 appears least accessible in DLB rather than MSA. Please clarify this point or reconcile the discrepancy.

Authors: We thank the reviewer for this question. Overall, both techniques showed that K80 is the least protected in the PD strain. However, the reviewer is right that, based on molecular painting K80 is least accessible in the DLB vs MSA strain, but this is the other way around based on LiP-MS (Figure 1). We clarified this in the manuscript (lines 225-243).

There could be several reasons for this discrepancy. LiP-MS and molecular painting probe different aspects of protein structural accessibility. LiP-MS detects flexible backbone regions accessible to protease cleavage. Consequently, rigid amyloid segments can show a low degree

of protease susceptibility despite the presence of exposed side chains, whereas molecular painting measures residue reactivity, which can still be high for structurally rigid protein regions. Thus, LiP-MS and molecular painting appear highly complementary for yielding a more complete structural map through their abilities to probe in detail protease susceptibility and report on side chains exposure to solvent-exposure, respectively.
We added the following text in the corresponding section of the revised manuscript (lines 225-243):

"Covalent painting revealed that αSyn N-terminal part was most accessible to solvent in the DLB strain, while it was least accessible in the MSA strain (Figure 1F). It further showed that αSyn NAC region (residue 80) was most accessible in the PD strain compared to DLB and MSA strains (Figure 1B). LiP-MS showed that αSyn NAC and pre-NAC regions were most protected in the DLB and MSA strains as compared to PD strains (Figure 1G) in overall agreement with covalent painting. Furthermore, DLB-derived fibrils were more protected than PD, both in the NAC region based on covalent painting (residue 80) and LiP-MS analysis, and in the pre-NAC region based on LiP-MS (Figure 1F, G). The C-terminal stretch spanning residues 97-117 showed a structural difference between MSA and PD strains; this was less pronounced between the MSA and DLB strains, and we observed no significant difference in C-terminal structure between the PD and DLB strains (Figure 1H). The apparent discrepancy observed at residue 80 between covalent labeling and LiP-MS results for DLB and MSA strains might be explained by the fact that LiP-MS and molecular painting probe different aspects of protein structural accessibility. LiP-MS detects flexible backbone regions accessible to protease cleavage. Consequently, rigid amyloid segments can show a low degree of protease susceptibility despite the presence of exposed side chains, whereas molecular painting measures residue reactivity, which can still be high for structurally rigid protein regions. Thus, LiP-MS and molecular painting are highly complementary, providing a more complete structural map by jointly probing protease susceptibility and side-chain exposure. "

5. Complementarity of LiP-MS and Covalent Painting: While both methods are informative, the correlation between their results appears limited to us. The authors may consider further emphasizing their "complementarity" in terms of structural resolution and coverage.
LiP-MS and molecular painting interrogate different dimensions of protein structural accessibility. LiP-MS detects backbone regions that are both accessible and sufficiently flexible for protease cleavage, so rigid amyloid segments often appear protected even when some side chains are solvent-exposed. In contrast, molecular painting measures the chemical reactivity of specific residues (e.g., lysines) toward labeling reagents, which can remain high on structurally rigid surfaces. This fundamental difference means that full correlation between the two datasets is not expected. Rather, their complementarity lies in the fact that LiP-MS offers high resolution on backbone flexibility, while molecular painting provides broader coverage of solvent-exposed side chains, together yielding a more complete structural and surface accessibility map.
We added the following text in the corresponding section of the revised manuscript (lines 237-243):
"LiP-MS and molecular painting probe different aspects of protein structural accessibility. LiP-MS detects flexible backbone regions accessible to protease cleavage. Consequently, rigid amyloid segments can show a low degree of protease susceptibility despite the presence of exposed side chains, whereas molecular painting measures residue reactivity, which can still be high for structurally rigid protein regions. Thus, LiP-MS and molecular painting are highly complementary, providing a more complete structural map by jointly probing protease susceptibility and side-chain exposure."

6. Line 282 and 325: These sections appear to describe results from the same experiment? Please clarify.

The reviewer is correct that the experimental set-up is technically the same. However, the data presented here were from two independent experiments. In both of these experiments,

we saw similar behaviour of the strains (e.g., higher accumulation of DLB compared to PD and MSA strains).

We added the following text into the revised manuscript (lines 338-339):

“This setup is the same as that described above and corresponds to a further repetition of the experiment. “

7. Line 864: The final statement regarding the number of altered proteins for drug development seems exaggerated. As compared to the total proteome?

Following the reviewer’s comment, we have toned down the statement. Indeed, we meant it in comparison to the total proteome. The revised final statement reads as follows (lines 938-940):

“Our results present a resource of structural changes of proteins and protein interfaces that can be prioritized in follow-up functional studies and might serve in the future as potential drug targets. “

8. PMCA Clarification: Although PMCA-based amplification has been published previously, it would be helpful to briefly describe its workflow and clarify again its fidelity. Additionally, it remains unclear to us how the patient-derived αSyn were sufficient for the large number of experiments presented? Were all fibrils used PMCA-amplified? Please clarify.

As emphasized by the reviewer, the amplification procedure we use has been described at length in other papers we refer to. We agree though with the reviewer that this is important to describe. We therefore added the following section in the methods section (lines 948-985):

“Frozen brain tissues were weighed in falcon tubes (15 or 50 ml depending on the total weight). The samples were diluted in PMCA buffer (150 mM KCl, 50 mM Tris–HCl pH 7.5, containing cOmplete protease inhibitors cocktail (Roche) and PhosSTOP (Roche), following the manufacturers recommendations) to obtain a homogenate at 20% (weight:volume). Tissue homogenization was performed by sonication using an SFX 150 Cell Disruptor sonicator with a 3.17 mm microtip probe (Branson) for 1 min, with 10 s pulses followed by 10 s pauses in a biosafety level 3 environment (BSL-3). The resulting homogenates were aliquoted, flash frozen in liquid nitrogen and stored at − 80 °C. PMCA was performed the following way: the patient brain homogenates were diluted 10 fold (e.g. 2% weight:volume) into 1.5 ml Eppendorf tubes containing 500µl of PMCA buffer containing monomeric αSyn (100µM). The resulting mix was split in two tubes of PCR strips (BIOplastics, Landgraaf, The Netherlands). The first cycle of PMCA amplification was performed in octuplicates for each patient using the Q700 generator and a 431MPX horn (Qsonica, Fisher scientific, Illkirch, France). The power of the horn was set to 30% of maximal amplitude. The program of amplification consisted in 15 s of sonication and 5 min pause at 31 °C. Every hour, 5 µl were withdrawn from each tube of one set of quadrupliates and diluted in 300 µl of 10 µM of Thioflavin T in water the increase in Thioflavin T fluorescence was measured using a Cary Eclipse Fluorescence Spectrophotometer (Agilent, Les Ulis, France) set at 440 nm for excitation and 480 nm for emission. Cycles 2, 3, and 4 were performed using the same procedure, except that 1% of the product from the preceding PD- or DLB-PMCA amplification cycle, or 5% of the product from the preceding MSA-PMCA cycle, was used to seed the aggregation of monomeric αSyn (100 µM). Fibrils resulting from the fourth amplification cycle were spun for 30 min at 50,000g, 30°C in a CN150NX table top ultracentrifuge (Hitachi). The amount of monomeric αSyn in the supernatant was assessed spectrophotometrically allowing the precise determination of the amounts of pelleted fibrils. The latter were resuspended in adjusted volumes of phosphate-buffered saline (PBS) buffer to a final concentration of 100µM. The resuspended fibrils were fragmented when used in cell culture experiments by sonication for 20 min in 2-ml Eppendorf tubes in a Vial Tweeter powered by an ultrasonic processor UIS250v (250 W, 2.4 kHz; Hielscher Ultrasonic, Teltow, Germany), maintained in a water bath at 20°C. The unfragmented or fragmented fibrils were

aliquoted, flash frozen in liquid nitrogen and stored at − 80 °C. The morphology of the PMCA amplified αSyn fibrils was assessed before and after fragmentation by transmission electron microscopy in a Jeol 1400 transmission electron microscope following adsorption onto carbon coated 200 mesh grids and negative staining with 1% uranyl acetate. The images were recorded using a Gatan Orius CCD camera (Gatan) the length of the unfragmented fibrils was highly variable while that of the fragmented fibrils was homogeneous (40±10 nm) on average. The Proteinase proteolytic patterns of the resulting fibrillar strains was analyzed by SDS-PAGE as previously described[3,13]."

Yes, for all these experiments we used PMCA-amplified fibrils.

9. Figure 2E: The concept of relative ubiquitination as presented is still not fully clear. The authors should improve the explanation of how this was measured and interpreted.

We thank the reviewer for raising this issue. We are happy to clarify our analysis. First, we searched for the GlyGly modification remnant of ubiquitin on αSyn in a targeted manner. We identified 4 sites that passed quality control; this involved retaining peptides based on consistent retention time, relative fragment ion intensity agreement between DIA and DDA (library) spectra, chromatographic co-elution of fragment ions, and reproducible MS peak shape across replicates. In addition, peptides observed in less than 50% of the 12 replicates per condition were discarded. The four identified peptides were: AK[GlyGly(K)]EGVVAAAEK (K12), EGVVAAAEK[GlyGly(K)]TK (K21), K[GlyGly(K)]TVEGAGSIAAATGFVK (K80), TVEGAGSIAAATGFVK[GlyGly(K)]K (K96). For each of the four ubiquitinated peptides, we compared intensities between conditions. The maximum detected intensity was set to 1 and the intensities of the same ubiquitinated peptide in the other conditions were normalized accordingly. We did not compare the intensities across different peptides, but across different conditions (PD, DLB, MSA, and Monomer) and assessed the significance of differences for each ubiquitinated peptide by statistical testing (Table EV3).

The following text has been added to the manuscript (lines 1191-1202):

"To identify ubiquitinated residues, we searched for the GlyGly(K) modification remnant of ubiquitin on the αSyn sequence in a targeted manner. Four ubiquitination sites (K12, K21, K80, and K96) passed our quality control after manual removal of peptides that might have been misidentified or were detected in fewer than 50% of the 12 replicates per condition. For each of the four ubiquitinated peptides (AK[GlyGly(K)]EGVVAAAEK (K12), EGVVAAAEK[GlyGly(K)]TK (K21), K[GlyGly(K)]TVEGAGSIAAATGFVK (K80), TVEGAGSIAAATGFVK[GlyGly(K)]K (K96)), intensities were compared across conditions (PD, DLB, MSA and Monomer). The maximum detected intensity for a given ubiquitinated peptide was set to 1, and intensities in the other conditions were normalized accordingly. Intensities were compared across conditions but not across different peptides. To assess significance, statistical testing was performed for each ubiquitinated peptide corresponding to its respective site (Table EV3)."

10. Line 1107: Please cite the version and reference of Spectronaut and clarify whether GlyGly(K) modifications were searched across the entire proteome or targeted to αSyn only. Genearlly, please improve the description here.

Thank you for the question. We used Spectronaut 17 and 18, and GlyGly(K) modification was searched in a targeted way.
The following text has been revised in the methods section of the manuscript (lines 1188-1202):

"Hybrid libraries were generated by Pulsar search in Spectronaut (Biognosys AG, Schlieren, Switzerland, versions 17 and 18)[14,15], using data from both Data-Independent Acquisition (DIA) and Data-Dependent Acquisition modes. Compared to the default settings, the digestion type

was set to ‘semi-specific’ To identify ubiquitinated residues, we searched for the GlyGly(K) modification remnant of ubiquitin on the αSyn sequence in a targeted manner. Four ubiquitination sites (K12, K21, K80, and K96) passed our quality control after manual removal of peptides that might have been misidentified or were detected in fewer than 50% of the 12 replicates per condition. For each of the four ubiquitinated peptides (AK[GlyGly(K)]EGVVAAAEK (K12), EGVVAAAEK[GlyGly(K)]TK (K21), K[GlyGly(K)] TVEGAGSIAAATGFVK (K80), TVEGAGSIAAATGFVK[GlyGly(K)]K (K96)), intensities were compared across conditions (PD, DLB, MSA and Monomer). The maximum detected intensity for a given ubiquitinated peptide was set to 1, and intensities in the other conditions were normalized accordingly. Intensities were compared across conditions but not across different peptides. To assess significance, statistical testing was performed for each ubiquitinated peptide corresponding to its respective site (Table EV3).”

11. Line 976: it is interesting to think about how much αSyn fibril was spiked into the various systems? Is the concentration used (e.g., 250 nM) based on physiological estimates, or chosen for practical detection reasons?

The concentration (e.g., 250 nM) we used falls within the range widely used in seeding experiments. At higher concentrations (e.g. 1µM) some toxicity, is observed with increased DNA fragmentation and intracellular reactive oxygen species[16,17].

Reviewer #2:

In this manuscript, Serdiuk et al. generate amyloid fibrils of recombinant alpha-synuclein (asyn) using protein misfolding cyclic amplification (PMCA) with PD, DLB and MSA brain homogenates. The authors characterise the resulting PMCA fibrils using TEM, limited proteolysis coupled to mass spectrometry (LiP-MS), ThT fluorescence and lysine accessibility (covalent molecular painting), showing that PMCA fibrils generated with different brain homogenates (PD, DLB and MSA) have distinct structural properties. The authors then perform proteome-wide LiP-MS on PMCA fibrils incubated with SH-SY5Y cells and lysates, and iPSC-derived cortical neurons. This supports the conclusion that the PMCA fibrils generated with different brain homogenates (PD, DLB and MSA) have distinct structural properties and suggests that they also have distinct interactomes. The authors then perform LiP-MS directly on PD, DLB and MSA brain homogenates, which matches a subset of the interactomes identified in the cell experiments. Finally, the authors over-express candidate protein degradation-related interactors identified by LiP-MS in HEK293 cells over-expressing asyn and incubated with PMCA fibrils. This results in reductions in pS129 asyn-positive cells in a fibril structure-dependent manner, consistent with the interactions predicted by LiP-MS.

Overall, this work is conducted to a high technical standard, combining the complementary expertise of the co-senior authors' groups in LiP-MS and amyloid fibril biology. The finding that alpha-synuclein fibrils with different structural properties have distinct interactomes with functional relevance to their degradation is conceptually important, as it supports the hypothesis that fibrils with distinct structures act as 'strains' to give rise to different diseases. The main caveat of this work is that it is not possible to assess the disease-relevance of many of the specific interactions identified, because it is not clear if the PMCA fibrils have disease-relevant structures. More detail on this and other, more minor, comments is provided below.

We thank the reviewer for the positive comments.

Major comments:
1. It is not clear if the PMCA method used to generate fibrils for this study produces disease-relevant structures. The structures of the ordered cores of fibrils from PD, DLB and MSA brain have been determined using cryo-EM (Schweighauser et al. 2020. Nature 585, 464-469; Yang et al. 2022. Nature 610, 791-795). However, the structure of the ordered core of fibrils generated by seeding recombinant alpha-synuclein with fibrils from MSA brain was different

(Lövestam et al. 2021. FEBS Open Bio 11, 999-1013), showing that such 'seeding' methods do not necessarily reproduce disease-relevant structures. The authors should compare the structures of the ordered cores of their PMCA fibrils to the published structures of the ordered cores of fibrils in PD, DLB and MSA. Ideally, this would be achieved by determining the structures using cryo-EM or NMR. If this is not possible, the authors could assess if their TEM analysis (projected widths, helical crossover distances), covalent molecular painting (exposed lysine residues) and LiP-MS analysis (protease-accessible sites) are consistent with the published structures. In the absence of such analysis, it is also not clear how reliable the authors' structural findings relating to regions outside of the ordered fibril cores are.

We agree with the reviewer on the importance of structurally comparing amplified fibrils to fibrils in vivo, i.e., in patient brains. This would require (i) resolving disease-specific fibril structures *in situ* (within patient brain tissue) at high resolution, and (ii) characterizing all fibrillar polymorphs present in samples amplified from brain tissue with detailed particle classification. Such a study would be technically very challenging and is beyond the scope of this study, despite recent advances in *in situ* cryo-electron tomography (cryo-TEM).

We note that the structures the reviewer mentions have been solved by cryo-EM on Sarkosyl precipitated material from patient brains. Notably, cryo-EM resolves only the “most solvable” conformations within highly heterogeneous fibril samples, often representing a minority (~12–30%) of the total fibril population (Schweighauser et al. 2020, Yang et al, 2022). Importantly, the authors of these studies observed that only a minority of fibrils exhibited helical twist—necessary for structure determination by helical reconstruction—further highlighting that these resolved structures represent just a subset of fibril polymorphs fractionated from the brain homogenates in the presence of the detergent Sarkosyl by ultracentrifugation through sucrose gradients. Consequently, comparison of TEM parameters of Sarkosyl precipitated fibrils with TEM parameters of PMCA amplified fibrils in terms of projected widths or helical crossover distances must be interpreted with caution.

Furthermore, the Sarkosyl treatment and differential centrifugation that have been used to prepare samples for structural characterization can bias the sample composition and even alter fibril conformations. Sarkosyl solubilizes significant fractions of disease-specific fibrils in a disease-dependent manner (up to 48% in PD and 20% in MSA within 30 min[10]), thus challenging the assumption that Sarkosyl-precipitated fibrils accurately represent all in vivo structures. Finally, several reports indicate that such purified fibrils exhibit poor seeding propensity[10].

Nevertheless, in accordance with the reviewer’s suggestion, we mapped our molecular painting data onto published Sarkosyl-extracted fibril structures of MSA (Figure R2). Molecular painting was selected over LiP-MS for this analysis to avoid potential confounding factors from PK secondary cleavages during limited proteolysis. Note that such potential secondary cleavage events would not affect the relative quantification analyses we performed in our study, as we previously showed[18,19], but would affect the analyses requested by the reviewer. We now mapped the average fold changes of Lys accessibilities that we had determined by covalent painting for the MSA polymorph vs monomer (Figure 1B) onto the MSA structure[20], (Figure R2).

This mapping revealed a correlation between lysine protection and the structural core identified by cryo-EM, since we observed strong protection of the core residues Lys 80, 58, 34, 32, 96, but no or low protection for Lys 97, which is solvent-exposed. For Lys 60, also a core residue, we observed no such protection, which might be due to the poor reactivity of this Lys, even in monomeric αSyn and, as a consequence, difficulties in measuring protection in the MSA amplified fibrils. The major discrepancy we observed was for Lys 43 and 45, which are located in the core but appear as non-protected in our averaged covalent painting results. Interestingly, when we examined individual patients, we found that one of the three patients

(MSA 080) did show significant protection at these residues (Figure R2, right panels); we thus present these data for individual patients in Appendix Figure S1.

We note as well that the cryo-EM structure of MSA fibrils lacks information for the αSyn N-terminal end. We observed protection for Lys 21 and 23 (i.e., near the N terminus) suggesting that the αSyn N terminus is folded and /or interacts with the MSA fibril core.

No such comparison is possible for PD and DLB strains as the cryo-EM structure lacks inter-protofilament interfaces that affect amino-acid residues exposure to solvent. It remains to be determined whether the PD and DLB fibrils are indeed single-protofilament structures or whether they represent fibrils partially disassembled by Sarkosyl.

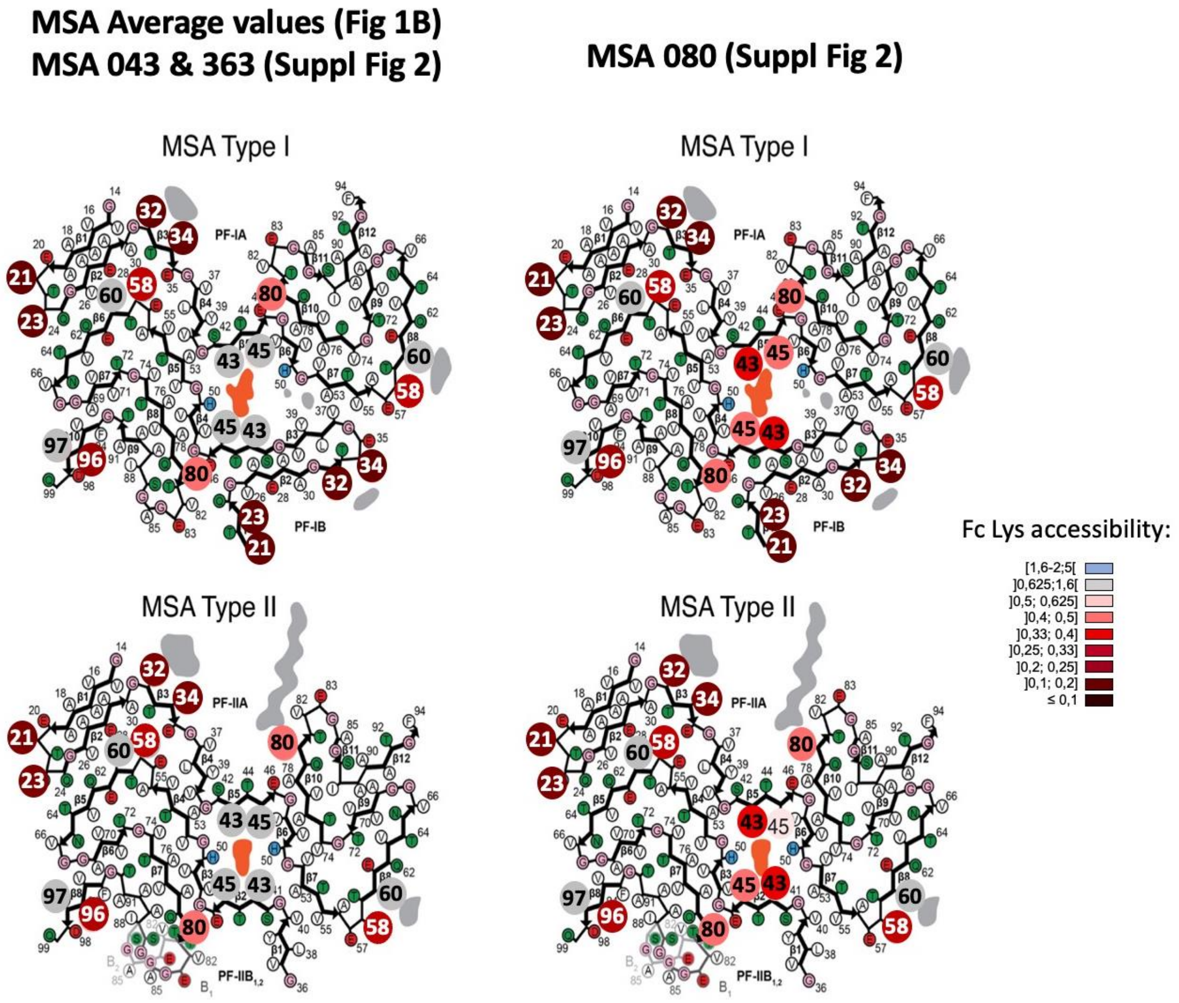

Figure R2. Mapping of molecular painting results on MSA-amplified fibrils onto the structures of Sarkosyl-precipitated fibrils from MSA[20]. The top and bottom panels on the left show fold changes of Lys accessibilities to NHS-biotin, as determined by covalent painting, for MSA type I and type II fibrils, respectively. Average fold changes of Lys accessibility for the three MSA patients relative to monomers are shown. Darker red shades correspond to lower accessibility compared to the monomer. The panels on the right show the same covalent painting data but for a single patient (MSA 080). All lysine residues within the amino-acid stretch 21 to 97 are mapped as large, filled circles, and labeled with the same scale and color code as in Figure 1B. For the remaining residues, negatively charged residues are shown in red, positively charged residues in blue, polar residues in green, apolar residues in white, and glycines in pink. Thick connecting lines with arrowheads indicate β-strands.

The following additional arguments support disease-relevance of our PMCA-amplified fibrils:

- We have previously shown that our amplification procedure works with high fidelity for synthetic fibrils as we were able to reproduce the PK digestion pattern of pure *de novo* assembled fibrils and ribbons before and after amplification (Figure R3):

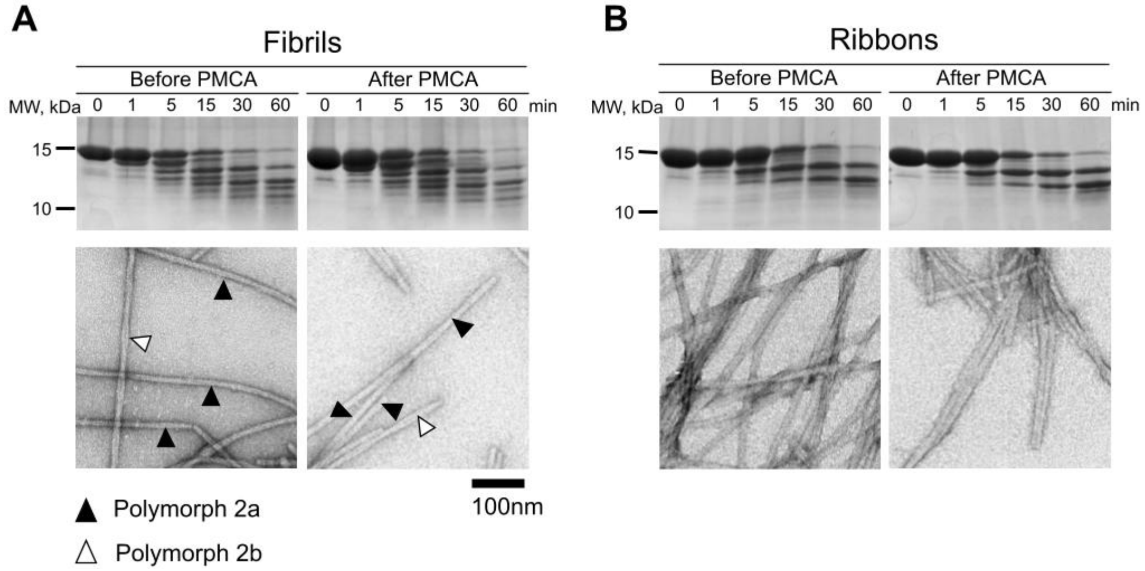

Figure R3. Verification of PMCA amplification fidelity, using two structurally distinct in vitro assembled fibrils. (A) “Fibrils” proteinase K proteolytic profiles before and after amplification, and negative stain transmission electron micrographs of Polymorph 2a/b (PDB id:6SST, 6SSX) filaments before and after amplification. Limited proteolysis pattern indicates a similar accessibility to protease indicative of a common fold. Typical helical repeat signature of Polymorph 2a/b is indicated by black for Polymorph 2a, and white for Polymorph 2b arrowheads. (B) “Ribbons” filaments proteinase K proteolytic profiles before and after amplification, and negative stain TEM images before and after amplification. Limited proteolysis pattern indicates a similar accessibility to protease, indicative of a common fold. The typical non helical signature of ribbons is observed before and after PMCA (taken from[21]).

- We have previously shown that fibrils amplified directly from native brain homogenates (under exactly the same conditions as in the current study) triggered different pathology in cellular and animal models, and importantly, that amplified fibrils could faithfully capture disease-specific effects of brain homogenates themselves[13]. Most strikingly, patient-derived amplified MSA fibrils consistently triggered the strongest response in terms of nigrostriatal neurodegeneration, αSyn pathology, spreading, motor deficits, and inflammation upon inoculation into rat brain, as was also seen for MSA brain homogenates. This strongly suggests that the structure-pathology relationship of αSyn fibrils is preserved in the amplification process. To our knowledge, there is no equivalent functional data for brain-derived fibrils prepared with the alternative Sarkosyl-precipitation based protocol.

- Our ThT data on both amplified fibrils and brain homogenates recapitulate previous findings showing lower ThT emission for MSA fibrils compared to PD fibrils in human CSF[9] and mouse brain[12].

- Fibril amplification is a well-established method used by many research groups in the field (over 200 occurrences in Medline), including groups of Virginia Lee[22,23], Björn Falkenburger[24], Claudio Soto[9,25], Paul Kotzbauer[26]. This method is based on the fundamental principle governing the prion concept, i.e., the ability of the aggregated form of infectious protein aggregates to grow through recruitment of their constituent protein and imprint their intrinsic structure on the latter (over 23.500 studies in Medline).

- Reviewer 2's concerns are based in part on Lövestam S, et al. Seeded assembly in vitro does not replicate the structures of α-synuclein filaments from multiple system atrophy (FEBS Open Bio. 2021[27]). We would like to emphasize here that the authors of that study used Sarkosyl-precipitated fibrils as seeds, instead of patient native brain homogenates as we do, without optimizing seed concentration or performing seeds fragmentation. They also conducted amplification at low pH (6.5), which may favour the formation of a different set of polymorph(s) as described[28], and did not include a control demonstrating that their setup could amplify different synthetic fibrils with high structural fidelity, as we did (Figure R3). All of these points weaken the conclusions of Lövestam et al[27], and do not apply to our own work.

- We note that, beyond comparing the structures of αSyn strains, the main point of this paper is more broadly to reveal the molecular players and processes that are affected in different synucleinopathies. We have assessed this aspect (i.e., structurally altered proteins) also directly in brain material of patients. We could validate some hits from the cell seeding model with amplified fibrils directly in native patient brain homogenates, making these very interesting candidates for prioritization as therapeutic targets. We also recapitulated our finding of structural differences between αSyn strains in patient brain homogenates. In other words, our work has revealed proteins and structural regions that change both in cell culture models and in human brain. Notably, for a subset of these proteins we found that the structural changes occurred exactly on the same sites (Figure 7F, H).

2. There is a general lack of validation of candidate fibril interactors identified by LiP-MS. In Fig. 3, the authors validate the interaction of UCHL1 (identified in LiP-MS of PMCA fibrils incubated with SH-SY5Y cell lysates) with alpha-synuclein monomers and all PMCA fibrils. It would be beneficial to also validate candidate fibril interactions identified by LiP-MS of PMCA fibrils incubated with SH-SY5Y cells and iPSC-derived cortical neurons, as well as of the human brain homogenates. In additiona, it would be helpful to validate candidate interactors that are specific to each of the alpha-synuclein species (monomer and PD/ DLB/ MSA homogenate-derived PMCA fibrils). This would also help to confirm that there are alpha-synuclein species-specific interactors and that this is not instead dependent on the sensitivity of LiP-MS.
We thank the reviewer for their question.
We would like to stress that in our experiments, whether on fibril-treated SH-SY5Y cells, iPSCs-derived neurons, and patient's brain, LiP-MS is not necessarily reporting on direct interactions of αSyn fibrils. Rather, LiP-MS reveals structural changes of proteins, affected processes and pathways that can be downstream or upstream effects of the direct interactors, in other words direct and indirect interactors and more. Therefore, the LiP-MS hits for these experiments should be evaluated not on the direct binding level, but on the functional level, including strain specificity. This was exactly the goal of our CRISPR activation experiments (Figure 8C). The strain specificity of our functionally validated LiP-MS hilts, UBE3A, TRIM25, HUWE1, and VCP was preserved at the functional level.

We nonetheless chose to validate the binding of UCHL1 because it was a hit in multiple LiP-MS screens as it is a genetic risk factor for PD. We note that we had also validated the MSA-specific binding of VCP to αSyn fibrils (Figure 8D) which should address the reviewer's request.

We agree that further validation of our LiP-MS hits would be very interesting, and in fact could serve as an avenue for future research directions. We have now added an AP-MS experiment probing the interactions of UBE3A with αSyn (Figure R5, newly added Appendix Figure S30). We pulled-down UBE3A from the SH-SY5Y lysate (Methods) that was spiked with the disease-specific αSyn strains. In line with LiP-MS, reciprocal AP-MS showed interaction of UBE3A with MSA and PD but not with the DLB αSyn strain, further supporting the specificity of the LiP-MS-identified interactions.

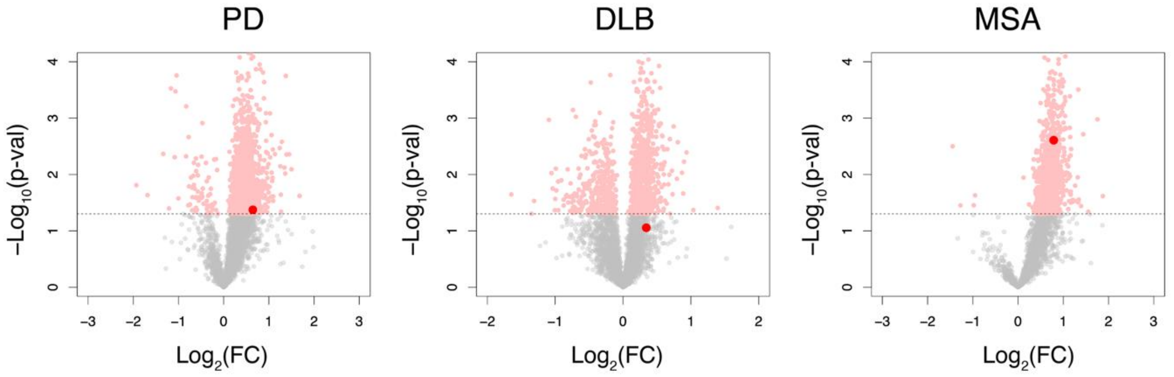

Figure R5 (**Appendix Figure S30**). AP-MS validation of UBE3A interaction with the MSA and PD patient-derived fibrillar polymorph. UBE3A was immunoprecipitated from SH-SY5Y lysate that was pre-incubated with each of the αSyn fibrillar polymorph. The volcano plots show proteins that change upon anti-UBE3A immunoprecipitation versus isotype-specific IgG control; the red dot indicates αSyn and the significance threshold is indicated (p-val< 0.05).

Beside the new data described above, we added further discussion to clarify this point in the discussion section (lines 341-343, 893-895 and lines 747-750):

"We use the term 'interactome' here to include both direct interactors of αSyn and proteins whose susceptibility to proteolysis changes indirectly as a consequence of αSyn addition."

"The strain specificity of our LiP-MS hits was functionally validated using a CRISPR activation setup. UBE3A, TRIM25, HUWE1, and VCP showed the same strain specificity in a CRISPR activation model, as suggested by LiP-MS screens."

"AP-MS data also supported the strain specificity of the interaction between UBE3A and PD or MSA αSyn strains, consistent with LiP-MS and CRISPR activation experiments (Appendix Figure S30). "

Minor comments:
1. Are differences in ubiquitination of PMCA fibrils in SH-SY5Y lysate due to differences in lysine availability measured by covalent molecular painting?

We thank the reviewer for the question. We think there are several overlapping effects that could cause the polymorph-specific ubiquitination we observe.

First, the structural differences between polymorphs and differential exposures of lysine residues could lead to differential ubiquitination, possibly because they are substrates for different E3 ligases. Consistent with this, we observed different sets of E3 ligases as hits for fibrils of the different strains. Second, since we assess relative ubiquitination within the complex lysate, cellular interactors might mask ubiquitination sites. All these factors may contribute collectively. We added a corresponding discussion to the revised manuscript (lines 278-281).

“Structural differences between the fibrils and their ability to interact differentially with proteins that may mask ubiquitination sites, including E3 ubiquitin ligases binding sites, may contribute to disease-specific ubiquitination patterns (Figure 2).”

2. Line 353: Should 'are' be 'were?'
We fixed this inconsistency in the text.

3. Line 370: Appears to be repetition of line 367 (referencing stability and ubiquitination)

We removed this repetition.

4. Presumably the lysis of the SH-SY5Y cells and iPSC-derived neurons prior to LiP-MS will contribute artificial interactions? Do the authors have any way to control for this?

The reviewer raises an important point. Cellular architecture is not preserved during the limited proteolysis step, and some organellar proteins are released from their native compartments (e.g., endoplasmic reticulum or mitochondria). This likely introduces noise into the LiP-MS signal and indeed may contribute to artificial interactions.

We did not include a specific control for this effect in the current study. We are actively developing an in-cell version of LiP-MS[29], which we anticipate will overcome these limitations by preserving cellular compartmentalization during limited proteolysis step. We have added the following statement to the revised manuscript (lines 816-821):

“One of the limitations of our current LiP-MS experimental setup is that it is performed after native cell lysis, where organellar architecture is not preserved. This lysis step may lead to artificial interactions and contribute noise to the LiP-MS signal. Consequently, interactions detected involving proteins from subcellular compartments (e.g., lysosomal proteins) should be interpreted with caution. In the future, in-cell LiP-MS may provide conditions that more accurately reflect the in-cell environment.[29]”

5. Line 698: is Why asyn overexpression used here and not in previous cell experiments? Does this lead to any spontaneous fibril formation that could confound the effects of the PMCA fibrils? Do the authors observe seeded aggregation of the over-expressed asyn (i.e. is it the seeds or seeded aggregates that become phosphorylated at S129)?

We understand the reviewer concerns. In most of our experiments we used SH-SY5Y cells, which show very low expression of αSyn, to have a clear structural signal from the disease-specific fibrils and characterize their processing independently from templating processes. For the experiments the referee mentions (i.e., screening for effects on pSer129), we turned to the HEK overexpression system because we have observed in the past that to achieve the formation of pathological pSer129 positive inclusions, the presence of endogenous αSyn is critical[3,30]. In fact, we observed that endogenous αSyn and not the exogenous seeds show the pSer 129 signature. Although no spontaneous fibril formation is detected in this system, inclusions containing phosphorylated αSyn are formed and are thought to be the pathological hallmark of αSyn toxicity[31]. We would like finally to stress that iPSC-derived cortical neurons, which we also use for LiP-MS screens, express physiological levels of endogenous αSyn and should capture potential seeding effects.

We added the following text to our manuscript (lines 719-721, and 701-704):

“This model was used to evaluate the effects of our selected hits on the formation of pathological inclusions of αSyn phosphorylated at position 129 (P-αSyn), which depends on the expression of endogenous αSyn[3,30,31]”

And:
“We therefore asked whether modulation of the identified degradation pathways affects cellular levels of pathogenic, aggregated αSyn, and whether strain specificity is preserved in the model of seeded αSyn aggregation when sufficient amount of endogenous αSyn is present for templating.”

6. Fig 8C: The n number not given and error bars are not present. Are two-tailed t-tests suitable, since multiple conditions are being compared to a control?

We thank the reviewer for pointing this out. We have added *n* values and error bars to the figure and legend. We used 4 replicates (i.e., separate cell cultures) for each strain and condition.
To account for multiple comparisons to a single control, we re-analyzed the data using a one-way ANOVA followed by Dunnett’s post hoc test. Dunnett’s test is specifically designed for comparing multiple conditions against a single control while controlling the family-wise error rate. The conclusions of the study remain largely unchanged, except that the effect of activation of HUWE1 on the PD strain is no longer statistically significant at p-adj <0.05, though it approaches the significance cut-off (p-adj=0.078). We also introduced different levels of significance, instead of a single threshold that we had used in our initial version of the manuscript.

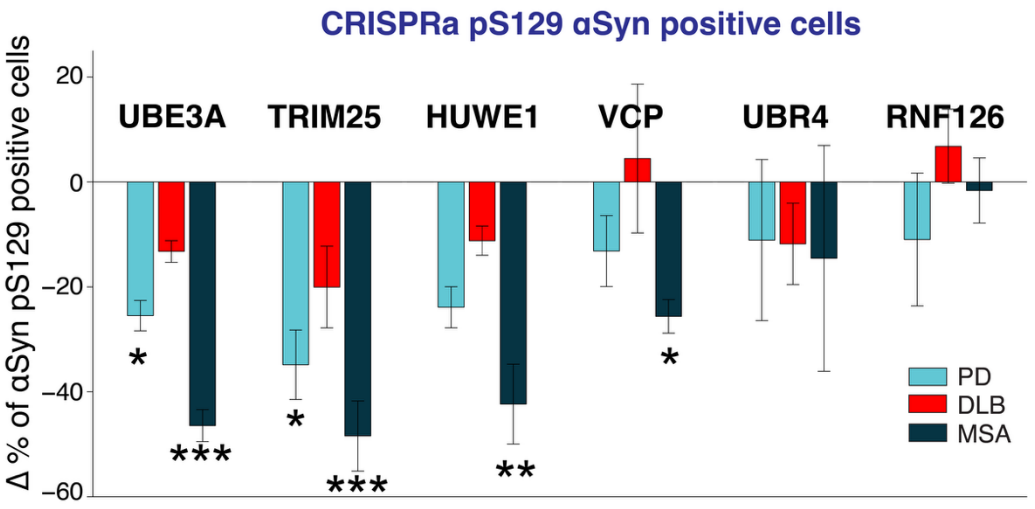

Figure R6 (Revised Figure 8C) (**C**) The plot shows the percentage of change of the fraction of p129 αSyn positive cells, upon exposure to the indicated fibrillar polymorph (PD in blue, DLB in red, MSA in grey) and activation of the indicated gene. As a control, non-activated cells infected with the same concentration of the fibrillar polymorphs were used. Asterisks indicate significance (*p-val<0.05, **p-val<0.005, ***p-val<0.001) relative to control based on a one-way ANOVA followed by Dunnett’s post hoc test. *N* = 4 biological replicates (independent cell cultures) per strain and condition.

Reviewer #3:

Summary
In their manuscript, Serdiuk et al study alpha-Synuclein aggregation and its effects in different synucleinopathies, Parkinson's Disease (PD), Dementia with Lewy Bodies (DLB) and Multiple System Atrophy (MSA). They make use of different MS-based techniques to study aggregation patterns in the different disease phenotypes and identify proteins interacting with and affected by each specific synucleinopathy. They first investigate stability and conformational

space for each PD, DLB, and MSA patient-derived αSyn polymorphs in patient brain lysates. The authors then analyze the stability of different aSyn polymorphs in a neuroblastoma cell lysate, and study the respective interactomes and structural proteomic effects upon exposure. They identify both known and novel interaction partners for each synucleinopathy, including E3 ligases such as HUWE1, DUBs such as UCHL1 and chaperones such as VCP. The authors then examine in aSyn-expressing HEK293 cells if overexpression of five of these candidate proteins affects clearance of aSyn fibrils after exposure to fibrillary polymorphs from the different synucleinopathies.
Taken together, the authors gain new insights into the structural dynamics of aSyn in different neurodegenerative diseases and identify potential targets for diagnostics and therapy. They make use of sensitive LC-MS-based approaches (LiP-MS and molecular painting) and validate and complement these findings biochemically and via NMR, working either directly with patient-derived samples or in cell culture model systems.

General remarks
The authors present a consistent and convincing story. The data are solid, and the conclusions are valid. Overall this manuscript provides important new data for a-Syn fibril structures and interaction partners in different neurobiological diseases, providing new insights that may have clinical relevance. aSyn fibrils are notoriously difficult to study in situ in their cellular environment, and structural proteomics methods such as LiP-MS offer a unique advantage to monitor both the target protein (aSyn) as well as other, affected proteins in different synucleinopathies. As such, this manuscript will be of interest to a broad audience of neurobiologists and I fully support publication in Mol Sys Biol once the points listed below have been addressed:

We thank the reviewer for the encouraging comments.

Major points
Overall this is comprehensive and nice piece of work. I only have a few points that need to be addressed, and some additional suggestions most likely for future studies.

Authors: we thank the reviewer for this encouraging comments.

- The authors use brain homogenates from cingulate cortex for PD and DLB, but cerebellum for MSA. Are there known differences in aSyn aggregates in different areas of the brain? What are the difference in aSyn for MSA in cingulate cortex, and for PD and DLB in the cerebellum? If no differences are expected this would serve as a good internal sanity check for physiological relevance.

We thank the reviewer for this thoughtful question. αSyn aggregates are indeed expected to differ across brain regions. For example, in PD and DLB, αSyn fibrils are typically not present in the cerebellum.

We have not (yet) analysed the structural features of αSyn across different brain regions. This is an interesting future research direction but was not the focus of our study. Rather, the brain regions we analysed are known to be affected in each respective pathology. The cerebellum is affected in MSA and the cingulate cortex in PD and DLB. We did not use substantia nigra tissue for PD because it is often severely affected in advanced PD cases, with disappearance of the dopaminergic neurons that are most sensitive to Lewy pathology and the remaining GABAergic neurons not particularly affected, making it technically challenging to perform consistent biochemical analysis.

Since the manuscript already included the information that the samples for different diseases were taken from different brain regions, we did not reinforce this statement. We now mention the issue of an explicit comparison of brain regions in the revised discussion section (lines 858-860):

"A systematic mapping of the structural features of αSyn across different brain regions depending on disease type, would be an interesting direction for follow up studies."

- The term "mitochondria" comes up in multiple experiments. In what way are mitochondria affected upon aSyn treatment of the cells - are they still "ok" morphologically, are they undergoing mitophagy or are there any other effects detectable using immunofluorescence or other orthogonal approaches?

We did not directly assess mitochondrial morphology or function in this study. However, we have previously shown that αSyn fibril polymorphs significantly impair mitochondrial respiration and ATP production in neurons[3,32] (Figure R7).
The link between αSyn aggregates and mitochondria has been extensively studied by various groups, including ours. In cell culture models, αSyn fibrillar treatment has been shown to induce mitochondrial fragmentation and condensation[33-35], which aligns with the presence of abnormal mitochondria observed in Lewy pathology in the patients' brain[36]. Aggregates of αSyn have also been shown to induce mitophagy[33]. In short, our own previous work and that of others supports a broad picture of αSyn-induced mitochondrial dysfunction[3].

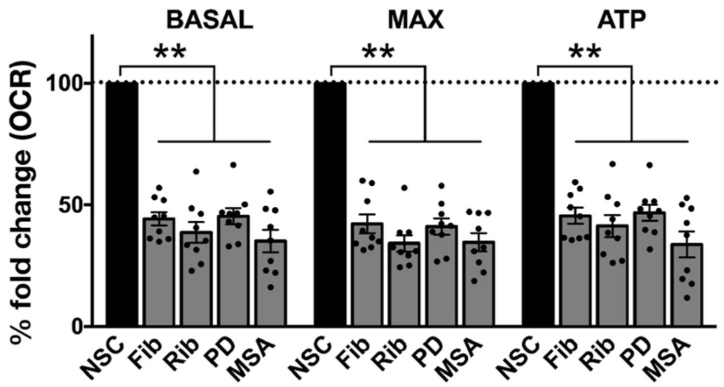

Figure R7. De novo fibrils, ribbons, or brain-amplified fibrils similarly reduced oxygen consumption rate (OCR), maximal respiration and ATP production measured 2 weeks post seeding ($n = 9$). $^{*}P < 0.05$, $^{**}P < 0.01$, $^{***}P < 0.001$, by one-way ANOVA followed by Tukey's multiple comparison test. (Taken from[3])

We added the following text to the manuscript (lines 483-490):

"This list includes multiple functionally-important mitochondrial proteins (Figure 4E) such as ATP synthase subunits, Opa1, the MICOS complex, VDAC3, the TOM complex and many other proteins performing crucial mitochondrial functions. Mitochondria disfunction has been shown to be involved in synucleinopathies, with supporting evidence at the genetic, environmental and pathological levels. In particular, αSyn was recently suggested to affect mitochondrial ATP synthesis in a conformation-dependent manner [32,37-41]. Mitophagy, mitochondrial fusion and fission, as well as the transport along microtubules have been demonstrated to be affected by αSyn[42,43]"

- Figure 2: The data shown in panels B and C appear inconsistent with panel D: The Western blot in panel D PD displays the strongest band for aSyn, whereas DLB shows the highest aSyn abundance in panel C. If this discrepancy derives from the loaded protein amounts in D (a-tub input control is higher too though), then it may make sense to show a normalized box plot with error bars.

Yes, indeed the issue raised by the reviewer can be explained by differences in the loading amount, based on loading control (α-tubulin). We have normalized the data and added a bar plot in Revised Figure 2D (and Figure R8) as requested. We can't, however, present these data with error bars, because the replication that we performed (Appendix Figure S9) was carried out independently as a biological replicate (on another gel and blot), with another batch of SH-SY5Y cells, independent lysate preparation, and independent control (we used GAPDH instead of α-tubulin as a loading control). Both Western blots showed the same results qualitatively, namely, relatively higher accumulation of DLB strain. Furthermore, the same effect was observed for intact SH-SY5Y cells and iPSCs-derived cortical neurons treated with the fibrillar polymorphs (Figures 5 and 6).

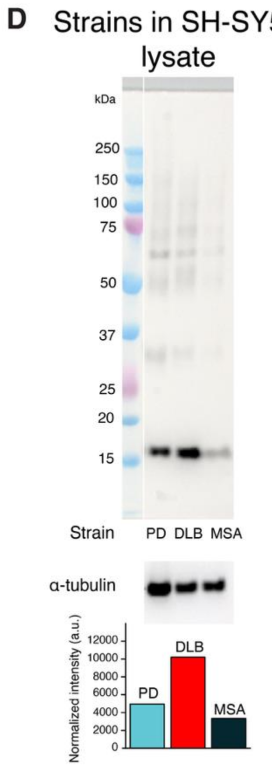

Figure R8 (**Revised Figure 2D**). Western blot analysis of αSyn (anti-αSyn antibody 5G4 (against aa 47-52)) after the same amount of αSyn (1.5 µg) of each strain was incubated for 15 min in cell lysate. SDS-PAGE was done using MES buffer. α-tubulin served as a loading control on the same blot. Integrated intensity normalized on loading control (a-tubulin) is shown on the lower panel.

- The authors show several affected interfaces on target proteins such as Rab13, DDC, CFAP298, VATB2, UCHL1 and VCP upon cellular aSyn exposure (Fig.3/5/6/7). While the primary amino acid sequence of these proteins may be quite different, did the authors examine if there are any conserved structural motifs or domains present in these proteins? A simple alignment is probably not sufficient, but a comparison of the respective domains may yield interesting insights.

We thank the reviewer for their interesting suggestions. We have performed a domain enrichment analysis for the candidate interactors of αSyn (Figure R9, Appendix Figure S18, Figure 3). We chose this dataset as it should be the most informative about direct binding interfaces of αSyn structures. While some indirect interactors may also be present, other experiments (e.g., exposure of live cells to αSyn fibrils) are expected to capture a higher proportion of downstream effects, thus further diluting the signal from direct interactions.

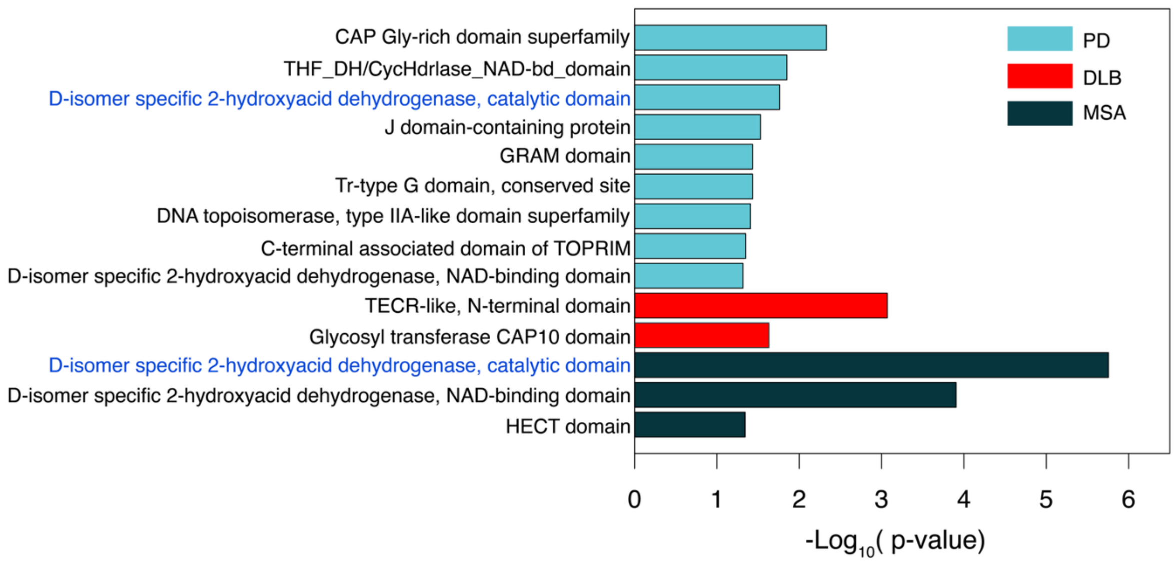

Figure R9 (**Appendix Figure S18**). Enrichment analysis of the protein domains for αSyn disease-specific strains hits revealed by LiP-MS. The domains enriched for multiple αSyn fibrillar polymorphs are shown in blue.

This analysis indeed revealed that the proteins that undergo conformational changes upon αSyn spike-in, among which we expect both candidate direct and indirect interactors of αSyn fibrils, are enriched in specific domains, and moreover that these enrichments are strain-dependent. Interestingly, the catalytic domain of D-isomer specific 2-hydroxyacid dehydrogenases was enriched for both PD and MSA trains, while NAD-binding domain also for PD strains. J domain containing proteins were enriched among candidate interactors of the PD strain as were proteins with a GRAM domain, which has an important role in membrane associated processes. These data have been added to the Appendix (Appendix Figure S18) and a comment was added (lines 419-427):

"We examined which protein domains are overrepresented among the protein regions that we identified as potential binding interfaces of αSyn structures (Appendix Figure S18). This analysis revealed that candidate direct and indirect interactors of αSyn fibrils are enriched in specific domains, and that these enrichments are strain-dependent. Interestingly, the catalytic domain of D-isomer–specific 2-hydroxyacid dehydrogenases was enriched in both PD and MSA strains, whereas the NAD-binding domain was enriched in PD strains only. J-domain–containing proteins were enriched among candidate interactors of the PD strain, as were proteins with a GRAM domain, which plays an important role in membrane-associated processes."

Optional points (involving significant amounts of work, not mandatory for this reviewer but food for thought for future studies:

We thank the reviewer for their great suggestions.

- The authors discuss protein stability extensively. The way to systematically study aSyn fibril stability - and its effect on other, associated proteins - would be to conduct a pulsed SILAC experiment and determine aSyn and proteome-wide protein synthesis and degradation rates. This would be straightfoward in the SH-SY5Y cells but would still be a substantial amount of work, and be particularly challenging for short time points.

We agree with the reviewer. The SILAC experiments would be indeed very interesting and powerful to understand the process of αSyn degradation and synthesis in the context of

different synucleinopathies in depth. We would be happy to address this question in a follow-up study.
We have now added a sentence suggesting these follow-up studies (lines 849-852):

"Furthermore, SILAC pulse-chase experiments on neurons seeded with heavy-labelled disease-specific fibrils could shed light on the dynamics of disease-specific fibril degradation and its interplay with endogenous αSyn seeding."

- There is a section on sarkosyl precipitation of aSyn fibrils: It would make sense to compare aSyn from sarkosyl precipitations from the brain to fibrils amplified directly from native patient brain homogenates using LiP-MS, to examine and compare the two model systems systematically.

We agree that comparing our PMCA-amplified structures with the structures of Sarkosyl precipitated fibrils would be interesting. Both methods are state-of the art but both methods also have different limitations. We will compare these two models systematically in the future. We added a corresponding sentence to the discussion (lines 775-777):

"Our current workflow combining LiP-MS and molecular painting may be used in the future to compare Sarkosyl-precipitated and PMCA-amplified fibrils."

Minor points
- Section 459-482 needs to be clarified a bit (the section on mitochondria is a bit vague, see above; the paragraph reads more like a concluding remark rather than the start of a section)

We revised the above-mentioned paragraph as follows (lines 483-490):

"This list includes multiple functionally-important mitochondrial proteins (Figure 4E) such as ATP synthase subunits, Opa1, the MICOS complex, VDAC3, the TOM complex and many other proteins performing crucial mitochondrial functions. Mitochondria disfunction has been shown to be involved in synucleinopathies, with supporting evidence at the genetic, environmental and pathological levels. In particular, αSyn was recently suggested to affect mitochondrial ATP synthesis in a conformation-dependent manner [32,37-41]. Mitophagy, mitochondrial fusion and fission, as well as the transport along microtubules have been demonstrated to be affected by αSyn[42,43]"

References:

1 Choi, S. G. *et al.* Proximity proteomics reveals unique and shared pathological features between multiple system atrophy and Parkinson's disease. *Acta Neuropathol Commun* **13**, 65 (2025). https://doi.org:10.1186/s40478-025-01958-5
2 Shrivastava, A. N. *et al.* Differential Membrane Binding and Seeding of Distinct alpha-Synuclein Fibrillar Polymorphs. *Biophys J* **118**, 1301-1320 (2020). https://doi.org:10.1016/j.bpj.2020.01.022
3 Tanudjojo, B. *et al.* Phenotypic manifestation of alpha-synuclein strains derived from Parkinson's disease and multiple system atrophy in human dopaminergic neurons. *Nat Commun* **12**, 3817 (2021). https://doi.org:10.1038/s41467-021-23682-z
4 Sanami, S., Purton, T. J., Smith, D. P., Tuite, M. F. & Xue, W. F. Comparative Analysis of the Relative Fragmentation Stabilities of Polymorphic Alpha-Synuclein Amyloid Fibrils. *Biomolecules* **12** (2022). https://doi.org:10.3390/biom12050630
5 Tarutani, A. *et al.* The Effect of Fragmented Pathogenic alpha-Synuclein Seeds on Prion-like Propagation. *J Biol Chem* **291**, 18675-18688 (2016). https://doi.org:10.1074/jbc.M116.734707
6 Abdelmotilib, H. *et al.* alpha-Synuclein fibril-induced inclusion spread in rats and mice correlates with dopaminergic Neurodegeneration. *Neurobiol Dis* **105**, 84-98 (2017). https://doi.org:10.1016/j.nbd.2017.05.014

7 Polinski, N. K. *et al.* Best Practices for Generating and Using Alpha-Synuclein Pre-Formed Fibrils to Model Parkinson's Disease in Rodents. *J Parkinsons Dis* **8**, 303-322 (2018). https://doi.org:10.3233/JPD-171248
8 Chisholm, T. S., Melki, R. & Hunter, C. A. Ligand Profiling as a Diagnostic Tool to Differentiate Patient-Derived alpha-Synuclein Polymorphs. *ACS Chem Neurosci* **15**, 2080-2088 (2024). https://doi.org:10.1021/acschemneuro.4c00178
9 Shahnawaz, M. *et al.* Discriminating alpha-synuclein strains in Parkinson's disease and multiple system atrophy. *Nature* **578**, 273-277 (2020). https://doi.org:10.1038/s41586-020-1984-7
10 Gram, H., Theologidis, V., Boesen, T. & Jensen, P. H. Sarkosyl differentially solubilizes patient-derived alpha-synuclein fibril strains. *Front Mol Biosci* **10**, 1177556 (2023). https://doi.org:10.3389/fmolb.2023.1177556
11 Tao, Y. *et al.* Structural mechanism for specific binding of chemical compounds to amyloid fibrils. *Nat Chem Biol* **19**, 1235-1245 (2023). https://doi.org:10.1038/s41589-023-01370-x
12 Burger, D. *et al.* Synthetic alpha-synuclein fibrils replicate in mice causing MSA-like pathology. *Nature* (2025). https://doi.org:10.1038/s41586-025-09698-1
13 Van der Perren, A. *et al.* The structural differences between patient-derived alpha-synuclein strains dictate characteristics of Parkinson's disease, multiple system atrophy and dementia with Lewy bodies. *Acta Neuropathol* **139**, 977-1000 (2020). https://doi.org:10.1007/s00401-020-02157-3
14 Gillet, L. C. *et al.* Targeted data extraction of the MS/MS spectra generated by data-independent acquisition: a new concept for consistent and accurate proteome analysis. *Mol Cell Proteomics* **11**, O111 016717 (2012). https://doi.org:10.1074/mcp.O111.016717
15 Baker, C. P., Bruderer, R., Abbott, J., Arthur, J. S. C. & Brenes, A. J. Optimizing Spectronaut Search Parameters to Improve Data Quality with Minimal Proteome Coverage Reductions in DIA Analyses of Heterogeneous Samples. *J Proteome Res* **23**, 1926-1936 (2024). https://doi.org:10.1021/acs.jproteome.3c00671
16 Bousset, L. *et al.* Structural and functional characterization of two alpha-synuclein strains. *Nat Commun* **4**, 2575 (2013). https://doi.org:10.1038/ncomms3575
17 Pieri, L. *et al.* Cellular response of human neuroblastoma cells to alpha-synuclein fibrils, the main constituent of Lewy bodies. *Biochim Biophys Acta* **1860**, 8-19 (2016). https://doi.org:10.1016/j.bbagen.2015.10.007
18 Piazza, I. *et al.* A Map of Protein-Metabolite Interactions Reveals Principles of Chemical Communication. *Cell* **172**, 358-372 e323 (2018). https://doi.org:10.1016/j.cell.2017.12.006
19 Cappelletti, V. *et al.* Dynamic 3D proteomes reveal protein functional alterations at high resolution in situ. *Cell* **184**, 545-559 e522 (2021). https://doi.org:10.1016/j.cell.2020.12.021
20 Schweighauser, M. *et al.* Structures of alpha-synuclein filaments from multiple system atrophy. *Nature* **585**, 464-469 (2020). https://doi.org:10.1038/s41586-020-2317-6
21 Burger, D. F., A.; Bousset, L.; Stahlberg, H.; Melki, R. . Cryo-EM structure of alpha-synuclein fibrils amplified by PMCA from PD and MSA patient brains. *bioRxiv* (2021). https://doi.org: https://doi.org/10.1101/2021.07.08.451588
22 Marotta, N. P. *et al.* Alpha-synuclein from patient Lewy bodies exhibits distinct pathological activity that can be propagated in vitro. *Acta Neuropathol Commun* **9**, 188 (2021). https://doi.org:10.1186/s40478-021-01288-2
23 Uemura, N. *et al.* alpha-Synuclein aggregates amplified from patient-derived Lewy bodies recapitulate Lewy body diseases in mice. *Nat Commun* **14**, 6892 (2023). https://doi.org:10.1038/s41467-023-42705-5
24 Brauer, S., Schniewind, I., Dinter, E. & Falkenburger, B. H. Recursive seed amplification detects distinct alpha-synuclein strains in cerebrospinal fluid of patients with Parkinson's disease. *Acta Neuropathol Commun* **13**, 13 (2025). https://doi.org:10.1186/s40478-024-01923-8
25 Siderowf, A. *et al.* Assessment of heterogeneity among participants in the Parkinson's Progression Markers Initiative cohort using alpha-synuclein seed amplification: a cross-sectional study. *Lancet Neurol* **22**, 407-417 (2023). https://doi.org:10.1016/S1474-4422(23)00109-6
26 Dhavale, D. D. *et al.* Structure of alpha-synuclein fibrils derived from human Lewy body dementia tissue. *Nat Commun* **15**, 2750 (2024). https://doi.org:10.1038/s41467-024-46832-5
27 Lovestam, S. *et al.* Seeded assembly in vitro does not replicate the structures of alpha-synuclein filaments from multiple system atrophy. *FEBS Open Bio* **11**, 999-1013 (2021). https://doi.org:10.1002/2211-5463.13110

28 Buell, A. K. *et al.* Solution conditions determine the relative importance of nucleation and growth processes in alpha-synuclein aggregation. *Proc Natl Acad Sci U S A* **111**, 7671-7676 (2014). https://doi.org:10.1073/pnas.1315346111
29 Franziska Elsässer, R. F., Felix Räsch, Karsten Weis, Natalie de Souza, Paola Picotti. Limited proteolysis-coupled mass spectrometry captures proteome-wide protein structural alterations and biomolecular condensation in living cells. *bioRxiv* (2025).
30 Courte, J. *et al.* The expression level of alpha-synuclein in different neuronal populations is the primary determinant of its prion-like seeding. *Sci Rep* **10**, 4895 (2020). https://doi.org:10.1038/s41598-020-61757-x
31 Luk, K. C. *et al.* Exogenous alpha-synuclein fibrils seed the formation of Lewy body-like intracellular inclusions in cultured cells. *Proc Natl Acad Sci U S A* **106**, 20051-20056 (2009). https://doi.org:10.1073/pnas.0908005106
32 Serdiuk, T. *et al.* Alpha-synuclein interacts with regulators of ATP homeostasis in mitochondria. *Nat Commun* **16**, 7651 (2025). https://doi.org:10.1038/s41467-025-62895-4
33 Lurette, O. *et al.* Aggregation of alpha-synuclein disrupts mitochondrial metabolism and induce mitophagy via cardiolipin externalization. *Cell Death Dis* **14**, 729 (2023). https://doi.org:10.1038/s41419-023-06251-8
34 Faustini, G. *et al.* Mitochondria and alpha-Synuclein: Friends or Foes in the Pathogenesis of Parkinson's Disease? *Genes (Basel)* **8** (2017). https://doi.org:10.3390/genes8120377
35 Kamp, F. *et al.* Inhibition of mitochondrial fusion by alpha-synuclein is rescued by PINK1, Parkin and DJ-1. *EMBO J* **29**, 3571-3589 (2010). https://doi.org:10.1038/emboj.2010.223
36 Shahmoradian, S. H. *et al.* Lewy pathology in Parkinson's disease consists of crowded organelles and lipid membranes. *Nat Neurosci* **22**, 1099-1109 (2019). https://doi.org:10.1038/s41593-019-0423-2
37 Borsche, M., Pereira, S. L., Klein, C. & Grunewald, A. Mitochondria and Parkinson's Disease: Clinical, Molecular, and Translational Aspects. *J Parkinsons Dis* **11**, 45-60 (2021). https://doi.org:10.3233/JPD-201981
38 Malpartida, A. B., Williamson, M., Narendra, D. P., Wade-Martins, R. & Ryan, B. J. Mitochondrial Dysfunction and Mitophagy in Parkinson's Disease: From Mechanism to Therapy. *Trends Biochem Sci* **46**, 329-343 (2021). https://doi.org:10.1016/j.tibs.2020.11.007
39 Przedborski, S., Tieu, K., Perier, C. & Vila, M. MPTP as a mitochondrial neurotoxic model of Parkinson's disease. *J Bioenerg Biomembr* **36**, 375-379 (2004). https://doi.org:10.1023/B:JOBB.0000041771.66775.d5
40 Wang, X. *et al.* Pathogenic alpha-synuclein aggregates preferentially bind to mitochondria and affect cellular respiration. *Acta Neuropathol Commun* **7**, 41 (2019). https://doi.org:10.1186/s40478-019-0696-4
41 Ganjam, G. K. *et al.* Mitochondrial damage by alpha-synuclein causes cell death in human dopaminergic neurons. *Cell Death Dis* **10**, 865 (2019). https://doi.org:10.1038/s41419-019-2091-2
42 Grassi, D. *et al.* Identification of a highly neurotoxic alpha-synuclein species inducing mitochondrial damage and mitophagy in Parkinson's disease. *Proc Natl Acad Sci U S A* **115**, E2634-E2643 (2018). https://doi.org:10.1073/pnas.1713849115
43 Pozo Devoto, V. M. & Falzone, T. L. Mitochondrial dynamics in Parkinson's disease: a role for alpha-synuclein? *Dis Model Mech* **10**, 1075-1087 (2017). https://doi.org:10.1242/dmm.026294

22nd Dec 2025

Manuscript Number: MSB-2025-13124R
Title: Structure-function relationship of alpha-synuclein fibrils derived from distinct synucleinopathies
Author: Tetiana Serdiuk
Virginie Redeker
Jimmy Savistchenko
Sandesh Neupane
Walther Haenseler
Yanick Fleischmann
Viviane Reber
Sabrina Keller
Cinzia Tiberi
Ruxandra Bachmann-Gagescu
Matthias Gstaiger
Thomas Braun
Roland Riek
Steve Gentleman
Adriano Aguzzi
Natalie de Souza
Ronald Melki
Paola Picotti

Dear Paola,

Thank you for submitting your revised manuscript. We have now received feedback from the three reviewers who were asked to re-evaluate your study. As you will see below, the reviewers think that most of their previous comments have been satisfactorily addressed, and they are overall supportive of the work.

Before we can formally accept your manuscript, we ask that you address the following remaining issues:

1. Reviewer #2 has raised a number of remaining concerns, most of which involve requests for clarification and minor textual revisions.

On a more editorial level, please do the following:

1. Please include up to five keywords in the manuscript file.

2. Remove "Authors' contribution" section from the manuscript file.

3. Please make sure all callouts are listed sequentially; Add missing callout for Fig. 6E, 7D.

4. "Conflict of interest statement" should be renamed to "disclosure and competing interests statement".

5. Please ensure that the funding information in the manuscript matches the details entered in the submission system. Currently, all grant numbers are missing from the submission system and need to be added.

6. Data availability
- The "data availability statement" should be renamed to "Data Availability."
- Provide specific URLs for the datasets with the following dataset identifiers: PXD058124, PXD058443, PXD058439, PXD058440, and PXD058444. Remove the reviewer token and ensure that the datasets will be made publicly available upon manuscript acceptance.
- The "Code availability statement" should be included within the Data Availability section, and the separate heading should be removed. The computer code used in the study should be deposited in a public database and the link should be provided as well.

7. The appendix file must be in PDF format, and please add page numbers for the listed items in the Table of Contents.

8. During our figure check, our data intergrity officer identified the following potential figure reuse:

• Possible blot reuse between Figure 2D and Appendix Figure S3
• Possible blot reuse between Figure 4C and Appendix Figure S20

Any figure reuse must be explicitly indicated in the corresponding figure legends.

In addition, there may be possible blot reuse within Figure EV1B; however, the image resolution is too low to allow proper assessment. Several blot and microscopy images appear pixelated and of insufficient quality for authentication. Please ensure that the entire image set is uploaded at high resolution using the original captured images.

9. Please provide a "standfirst text" summarizing the study in one or two sentences (approximately 250 characters, including space), three to four "bullet points" highlighting the main findings and a " visual abstract" (550px width and 400-600 px height, PNG format) to highlight the paper on our homepage.
Please refer to published papers for examples.

10. Since these files are rather complex, source file names, titles, legends, and manuscript callouts should be updated to "Dataset EV1-EV12" instead of Tables EV1, EV3, EV5, EV6, EV8, EV12-EV14, and EV16-EV19. Legends should be included as a separate tab or sheet within each Excel file. The remaining Tables EV2, EV4, EV7, EV9-EV11, EV15 should then be renumbered to Table EV1-EV7 with legends included above tables in each Excel file. Their callouts need to be updated accordingly as well.

11. The references need to be formatted according to the Molecular Systems Biology reference style. Please list up to 10 co-authors of a paper before adding et al. in the reference list. Citations should be listed in alphabetical order. DOI numbers should be removed for published articles.

12. Please download and fill our Reagents and Tools Table template (.docx), which you can find in our author guidelines: https://link.springer.com/journal/44320/submission-guidelines#structuredmethods.

When submitting your revised manuscript, please do not include the Reagents and Tools Table in the Methods section of the manuscript but upload it as a separate file choosing the file type "Reagent Table".

13. Please address the following issues in figure legends :
- Please note that the exact p values are not provided in the legends of figures 2b,c; 5c; 6e; 8c; EV-1c,d.
- Please indicate the statistical test used for data analysis in the legends of figures 2b,c; 5c; 6e; 7e; 8d; EV-1c,d.
- Please note that the box plots need to be defined in terms of minima, maxima, centre, bounds of box and whiskers, and percentile in the legends of figures 2b,c; 5c.
- Please note that information related to n is missing in the legends of 2b,c; 5c.

14. Sections need to be named and the order should be corrected: Title page - Abstract - Keywords - Introduction - Results - Discussion - Methods - Data Availability - Acknowledgements -Disclosure and Competing Interests Statement - References - Figure Legends - Table(s) - Expanded View Figure Legends.

Please resubmit your revised manuscript online, with a covering letter listing amendments and responses to each point raised by the referees.

As a matter of course, please make sure that you have correctly followed the instructions for authors as given on the submission website.

Thank you for submitting this paper to Molecular Systems Biology.

Kind regards,
Jingyi

Jingyi Hou, PhD
Senior Editor
Molecular Systems Biology

*** PLEASE NOTE *** As part of the EMBO Press transparent editorial process initiative (see our Editorial at https://dx.doi.org/10.1038/msb.2010.72 , Molecular Systems Biology will publish online a Review Process File to accompany

accepted manuscripts. When preparing your letter of response, please be aware that in the event of acceptance, your cover letter/point-by-point document will be included as part of this File, which will be available to the scientific community. More information about this initiative is available in our Instructions to Authors. If you have any questions about this initiative, please contact the editorial office (msb@embo.org).

Reviewer #1:

The authors addressed most of our questions in the revision. We are now supportive! Great job!

Reviewer #2:

The authors have added clarifying statements to the manuscript to satisfactorily address most of my comments. Comments that require additional clarification are below.

Regarding my major comment 1 that it was not clear if the PMCA method used to generate fibrils for this study produces disease-relevant structures:
- A structural comparison could be achieved using single-particle cryo-EM, without the need to use cryo-ET. However, I agree that this is beyond the scope of this study.
- The authors critique of cryo-EM structures of asyn fibrils from patient brain omits the fact that comparison of the 2D averages of fibrils without a helical twist with those of fibrils with a helical twist showed that they shared the same structure. As such, cryo-EM accounts for the vast majority of fibrils.
- I am not aware of any data showing that sarkosyl alters the composition/conformation of fibrils (of asyn or any other protein) from patient brains. The cited paper (Gram et al. 2023. Front. Mol. Biosci.) used asyn fibrils generated in vitro, so it is unclear how their findings relate to patient brain/disease. For tau fibrils from patient brains, the structures were the same when extracted using sarkosyl or immunoprecipitation in the absence of detergent (Shi et al. 2021. Acta Neuropathol. 141:697-708).
- The comparison of the authors' covalent molecular painting data with the cryo-EM structures of asyn fibrils from MSA shows that there is a poor correlation between lysine accessibility and if the residue is buried or exposed in the cryo-EM structures. Therefore, it would be prudent to include a sentence in the manuscript to say that it is not enitely clear if/how the structures of the PMCA-generated fibrils relate to those from patient brain, raising the possibility that findings that were not validated using patient brain homogenates may not relate to disease and will require validation.

Regarding my major comment 2 that the validation of interactions was lacking:
- The AP-MS experiment is a nice addition. Thank you also for clarifying that LiP-MS is not necessarily reporting on fibril interactions. In this case, the use of the terms 'interactome' and 'interactions' when referring to LiP-MS data appear to be inappropriate and should be removed unless an interaction is verified, such as for UCHL1, VCP and UBE3A.

Regarding my minor comment 5, questioning the use of asyn overexression:
- The first sentence added (lines 719-721) is helpful to clairfy why overexpression was used.
- However, the second sentence added (lines 701-704) does not appear to be supported by data. How do the authors know that templated seeding has occurred in the absence of comapring the structures of the seed and seeded fibrils? It appears that the term 'templating' should be removed.

Reviewer #3:

The authors conducted substantial experiments and addressed the points I raised in the initial revision. From my side this manuscript is suitable for publication in Molecular Systems Biology.

Molecular Systems Biology, MSB-2025-13124-R

'Structure-function relationship of alpha-synuclein fibrillar polymorphs derived from distinct synucleinopathies'

Response to all Referees and Editor

We thank the reviewers for their positive comments. We have addressed their requested clarifications and text modifications, as well as editorial requests.

Point-by-point response to the reviewers

Reviewer #1:

The authors addressed most of our questions in the revision. We are now supportive! Great job!

We thank the reviewer for their encouraging comments and support.

Reviewer #2:

The authors have added clarifying statements to the manuscript to satisfactorily address most of my comments. Comments that require additional clarification are below.

We thank the reviewer for their positive feedback.

Regarding my major comment 1 that it was not clear if the PMCA method used to generate fibrils for this study produces disease-relevant structures:
- A structural comparison could be achieved using single-particle cryo-EM, without the need to use cryo-ET. However, I agree that this is beyond the scope of this study.

We appreciate the reviewer's acknowledgement that a detailed structural comparison of PMCA-generated fibrils is beyond the scope of this study.

- The authors critique of cryo-EM structures of asyn fibrils from patient brain omits the fact that comparison of the 2D averages of fibrils without a helical twist with those of fibrils with a helical twist showed that they shared the same structure. As such, cryo-EM accounts for the vast majority of fibrils.

We acknowledge that the cryo-EM study compared 2D class averages of fibrils with and without a helical twist and reported similarities between these populations. However, establishing direct structural equivalence between twisted and non-twisted fibrils based solely on 2D class averages is not straightforward. Since these points were only discussed in the rebuttal and not in the manuscript, we did not introduce any modifications to the manuscript.

- I am not aware of any data showing that sarkosyl alters the composition/conformation of fibrils (of asyn or any other protein) from patient brains. The cited paper (Gram et al. 2023. Front. Mol. Biosci.) used asyn fibrils generated in vitro, so it is unclear how their findings relate to patient brain/disease. For tau fibrils from patient brains, the structures were the same when extracted using sarkosyl or immunoprecipitation in the absence of detergent (Shi et al. 2021. Acta Neuropathol. 141:697-708).

We agree that in the cited paper Gram et al., 2023 [1], the αSyn fibrils were not directly isolated from patient brain tissue but were instead amplified from cerebrospinal fluid. Nevertheless, this study

demonstrated that sarkosyl dissolves a substantial fraction of αSyn fibrils within 30 minutes (up to ~50%), and importantly, that this effect is strain-dependent.
We assessed the seeding propensity of pathogenic αSyn upon purifying it from 3 PD and 5 MSA patient brains in the presence of sarkosyl following the method described in Tarutani et al., 2018[2]. While the original brain homogenates had seeding propensity under the PMCA experimental conditions, no seeding was observed for the sarkosyl extracted αSyn under the same conditions even upon increasing its concentrations (10 fold as assessed P-αSyn quantification by filter trapping). We did not publish yet these observations.

Furthermore, while equivalent fibril structures have been reported for tau isolated from patient brains following either sarkosyl extraction or immunoprecipitation in the absence of detergent, comparable evidence is currently lacking for αSyn, as noted in the peer review file associated with the cryo-EM study of αSyn fibrils isolated from PD brain[3]. Since it cannot be assumed that different amyloid proteins behave identically with respect to detergent extraction, further investigations are needed to answer this question.

As suggested by the reviewer, we have added a sentence to the manuscript noting that:

"Currently, it is not entirely clear to what extent PMCA-generated fibrils fully recapitulate those derived from patient brain tissue; therefore, findings that have not been validated using patient brain homogenates may require further confirmation.". This sentence has been added to the Discussion (lines 833-835).

- The comparison of the authors' covalent molecular painting data with the cryo-EM structures of asyn fibrils from MSA shows that there is a poor correlation between lysine accessibility and if the residue is buried or exposed in the cryo-EM structures. Therefore, it would be prudent to include a sentence in the manuscript to say that it is not enitely clear if/how the structures of the PMCA-generated fibrils relate to those from patient brain, raising the possibility that findings that were not validated using patient brain homogenates may not relate to disease and will require validation.

See above. Such a sentence was added to the Discussion.

Regarding my major comment 2 that the validation of interactions was lacking:
- The AP-MS experiment is a nice addition. Thank you also for clarifying that LiP-MS is not necessarily reporting on fibril interactions. In this case, the use of the terms 'interactome' and 'interactions' when referring to LiP-MS data appear to be inappropriate and should be removed unless an interaction is verified, such as for UCHL1, VCP and UBE3A.

We thank the reviewer for acknowledging the addition of the AP-MS data for UBE3A interactions.

Regarding the terminology used, we note that the terms "interactome" or "interactors" have been broadly applied to describe biophysical changes in proteins detected upon addition of a molecule of interest to a proteome extract, as done here. This usage is common not only in LiP-MS studies by us and others, but also in studies employing the related thermal proteome profiling (TPP) approach. In TPP analyses, interactomes of a molecule of interest are identified by detecting proteins whose solubility changes upon addition of the molecule to a proteome extract.

For both TPP and LiP-MS, the ability to identify direct protein–molecule interactions from cell lysates has been demonstrated by us and others using a variety of positive controls and validation experiments (see for example Cappelletti et al, 2021[4], Piazza et al, 2020[5], Piazza et al, 2018[6],Zijlmans et al, 2023[7] , Searle, 2024[8], Li et al, 2025[9], Reinhard et al, 2015[10]). Since secondary effects, for example proteins exhibiting altered proteolytic accessibility due to changes in their binding to direct targets, can contribute to the observed readout for a fraction of identified

proteins, we have revised our terminology to "putative interactors/interactomes" throughout. For proteins with validated interactions, such as UCHL1, VCP, and UBE3A, we retained the term "interactions".

Regarding my minor comment 5, questioning the use of asyn overexression:
- The first sentence added (lines 719-721) is helpful to clairfy why overexpression was used.
- However, the second sentence added (lines 701-704) does not appear to be supported by data. How do the authors know that templated seeding has occurred in the absence of comapring the structures of the seed and seeded fibrils? It appears that the term 'templating' should be removed.

To address the reviewer's comment, we removed the term "templating" from this sentence. The revised version now reads as follows (lines 745-748):

"We therefore asked whether modulation of the identified degradation pathways affects cellular levels of pathogenic, aggregated αSyn, and whether strain specificity is preserved in the model of seeded αSyn aggregation when sufficient amount of endogenous αSyn is present."

Reviewer #3:

The authors conducted substantial experiments and addressed the points I raised in the initial revision. From my side this manuscript is suitable for publication in Molecular Systems Biology.

We thank the reviewer for their positive and encouraging comments.

References

1 Gram, H., Theologidis, V., Boesen, T. & Jensen, P. H. Sarkosyl differentially solubilizes patient-derived alpha-synuclein fibril strains. *Front Mol Biosci* **10**, 1177556 (2023). https://doi.org:10.3389/fmolb.2023.1177556

2 Tarutani, A., Arai, T., Murayama, S., Hisanaga, S. I. & Hasegawa, M. Potent prion-like behaviors of pathogenic alpha-synuclein and evaluation of inactivation methods. *Acta Neuropathol Commun* **6**, 29 (2018). https://doi.org:10.1186/s40478-018-0532-2

3 Yang, Y. *et al.* Structures of alpha-synuclein filaments from human brains with Lewy pathology. *Nature* **610**, 791-795 (2022). https://doi.org:10.1038/s41586-022-05319-3

4 Cappelletti, V. *et al.* Dynamic 3D proteomes reveal protein functional alterations at high resolution in situ. *Cell* **184**, 545-559 e522 (2021). https://doi.org:10.1016/j.cell.2020.12.021

5 Piazza, I. *et al.* A machine learning-based chemoproteomic approach to identify drug targets and binding sites in complex proteomes. *Nat Commun* **11**, 4200 (2020). https://doi.org:10.1038/s41467-020-18071-x

6 Piazza, I. *et al.* A Map of Protein-Metabolite Interactions Reveals Principles of Chemical Communication. *Cell* **172**, 358-372 e323 (2018). https://doi.org:10.1016/j.cell.2017.12.006

7 Zijlmans, D. W. *et al.* STPP-UP: An alternative method for drug target identification using protein thermal stability. *J Biol Chem* **299**, 105279 (2023). https://doi.org:10.1016/j.jbc.2023.105279

8 Searle, B. C. Characterizing protein-protein interactions with thermal proteome profiling. *Curr Opin Struct Biol* **89**, 102946 (2024). https://doi.org:10.1016/j.sbi.2024.102946

9 Li, K. *et al.* A peptide-centric local stability assay enables proteome-scale identification of the protein targets and binding regions of diverse ligands. *Nat Methods* **22**, 278-282 (2025). https://doi.org:10.1038/s41592-024-02553-7

10 Reinhard, F. B. *et al.* Thermal proteome profiling monitors ligand interactions with cellular membrane proteins. *Nat Methods* **12**, 1129-1131 (2015). https://doi.org:10.1038/nmeth.3652

9th Feb 2026
Manuscript number: MSB-2025-13124RR
Title: Structure-function relationship of alpha-synuclein fibrils derived from distinct synucleinopathies

Dear Dr. Picotti,

Thank you again for sending us your revised manuscript. We are now satisfied with the modifications made and I am pleased to inform you that your paper has been accepted for publication.

Your manuscript will be processed for publication by EMBO Press. It will be copy edited and you will receive page proofs prior to publication. Please note that you will be contacted by Springer Nature Author Services to complete licensing and payment information.

You may qualify for financial assistance for your publication charges - either via a Springer Nature fully open access agreement or an EMBO initiative. Check your eligibility: https://link.springer.com/journal/44320/how-to-publish-with-us

Should you be planning a Press Release on your article, please get in contact with embo_production@springernature.com as early as possible in order to coordinate publication and release dates.

If you have any questions, please do not hesitate to contact the Editorial Office. Thank you for your contribution to Molecular Systems Biology.

Sincerely,
Jingyi

Jingyi Hou, PhD
Senior Editor
Molecular Systems Biology

>>> Please note that it is Molecular Systems Biology policy for the transcript of the editorial process (containing referee reports and your response letter) to be published as an online supplement to each paper. If you do NOT want this, you will need to inform the Editorial Office via email immediately. More information is available here: https://link.springer.com/partners/embo-press/editorial-policies#Peer%20review

EMBO Press Author Checklist

Corresponding Author Name: Paola Picotti, Ronald Melki	
Journal Submitted to: Molecular Systems Biology	
Manuscript Number: MSB-2025-13124-T	

USEFUL LINKS FOR COMPLETING THIS FORM
The EMBO Journal - Author Guidelines
EMBO Reports - Author Guidelines
Molecular Systems Biology - Author Guidelines
EMBO Molecular Medicine - Author Guidelines

Reporting Checklist for Life Science Articles (updated January
This checklist is adapted from Materials Design Analysis Reporting (MDAR) Checklist for Authors. MDAR establishes a minimum set of requirements in transparent reporting in the life sciences (see Statement of Task: 10.31222/osf.io/9sm4x). Please follow the journal's guidelines in preparing your manuscript.
Please note that a copy of this checklist will be published alongside your article.

Abridged guidelines for figures
1. Data
The data shown in figures should satisfy the following conditions:
- the data were obtained and processed according to the field's best practice and are presented to reflect the results of the experiments in an accurate and unbiased manner.
- ideally, figure panels should include only measurements that are directly comparable to each other and obtained with the same assay.
- plots include clearly labeled error bars for independent experiments and sample sizes. Unless justified, error bars should not be shown for technical
- if n<5, the individual data points from each experiment should be plotted. Any statistical test employed should be justified.
- Source Data should be included to report the data underlying figures according to the guidelines set out in the authorship guidelines on Data

2. Captions
Each figure caption should contain the following information, for each panel where they are relevant:
- a specification of the experimental system investigated (eg cell line, species name).
- the assay(s) and method(s) used to carry out the reported observations and measurements.
- an explicit mention of the biological and chemical entity(ies) that are being measured.
- an explicit mention of the biological and chemical entity(ies) that are altered/varied/perturbed in a controlled manner.
- the exact sample size (n) for each experimental group/condition, given as a number, not a range;
- a description of the sample collection allowing the reader to understand whether the samples represent technical or biological replicates (including how many animals, litters, cultures, etc.).
- a statement of how many times the experiment shown was independently replicated in the laboratory.
- definitions of statistical methods and measures:
  - common tests, such as t-test (please specify whether paired vs. unpaired), simple χ2 tests, Wilcoxon and Mann-Whitney tests, can be unambiguously identified by name only, but more complex techniques should be described in the methods section;
  - are tests one-sided or two-sided?
  - are there adjustments for multiple comparisons?
  - exact statistical test results, e.g., P values = x but not P values < x;
  - definition of 'center values' as median or average;
  - definition of error bars as s.d. or s.e.m.

Please complete ALL of the questions below.
Select "Not Applicable" only when the requested information is not relevant for your study.

Materials

Newly Created Materials	Information included in the manuscript?	In which section is the information available? (Reagents and Tools Table, Materials and Methods, Figures, Data Availability Section)
New materials and reagents need to be available; do any restrictions apply?	Not Applicable	

Antibodies	Information included in the manuscript?	In which section is the information available? (Reagents and Tools Table, Materials and Methods, Figures, Data Availability Section)
For **antibodies** provide the following information:<br>- Commercial antibodies: RRID (if possible) or supplier name, catalogue number and or/clone number<br>- Non-commercial: RRID or citation	Yes	Materials and Methods

DNA and RNA sequences	Information included in the manuscript?	In which section is the information available? (Reagents and Tools Table, Materials and Methods, Figures, Data Availability Section)
**Short novel DNA or RNA including primers, probes**: provide the sequences.	Yes	Materials and Methods

Cell materials	Information included in the manuscript?	In which section is the information available? (Reagents and Tools Table, Materials and Methods, Figures, Data Availability Section)
**Cell lines:** Provide species information, strain. Provide accession number in repository **OR** supplier name, catalog number, clone number, and/**OR** RRID.	Yes	Materials and Methods
**Primary cultures:** Provide species, strain, sex of origin, genetic modification status.	Not Applicable	
Report if the cell lines were recently **authenticated** (e.g., by STR profiling) and tested for mycoplasma contamination.	Yes	Materials and Methods

Experimental animals	Information included in the manuscript?	In which section is the information available? (Reagents and Tools Table, Materials and Methods, Figures, Data Availability Section)
**Laboratory animals or Model organisms:** Provide species, strain, sex, age, genetic modification status. Provide accession number in repository **OR** supplier name, catalog number, clone number, **OR** RRID.	Not Applicable	
**Animal observed in or captured from the field:** Provide species, sex, and age where possible**.**	Not Applicable	
Please detail **housing and husbandry conditions**.	Not Applicable	

Plants and microbes	Information included in the manuscript?	In which section is the information available? (Reagents and Tools Table, Materials and Methods, Figures, Data Availability Section)
**Plants:** provide species and strain, ecotype and cultivar where relevant, unique accession number if available, and source (including location for collected wild specimens).	Not Applicable	
**Microbes:** provide species and strain, unique accession number if available, and source.	Not Applicable	

Human research participants	Information included in the manuscript?	In which section is the information available? (Reagents and Tools Table, Materials and Methods, Figures, Data Availability Section)
If collected and within the bounds of privacy constraints report on age, sex and gender or ethnicity for all study participants.	Yes	Materials and Methods

Core facilities	Information included in the manuscript?	In which section is the information available? (Reagents and Tools Table, Materials and Methods, Figures, Data Availability Section)
If your work benefited from core facilities, was their service mentioned in the acknowledgments section?	Yes	Acknowledgements

Design

Study protocol	Information included in the manuscript?	In which section is the information available? (Reagents and Tools Table, Materials and Methods, Figures, Data Availability Section)
If study protocol has been **pre-registered, provide DOI in the manuscript**. For clinical trials, provide the trial registration number **OR** cite DOI.	Not Applicable	
Report the **clinical trial registration number** (at ClinicalTrials.gov or equivalent), where applicable.	Not Applicable	

Laboratory protocol	Information included in the manuscript?	In which section is the information available? (Reagents and Tools Table, Materials and Methods, Figures, Data Availability Section)
Provide DOI OR other citation details if **external detailed step-by-step protocols** are available.	Not Applicable	

Experimental study design and statistics	Information included in the manuscript?	In which section is the information available? (Reagents and Tools Table, Materials and Methods, Figures, Data Availability Section)
Include a statement about **sample size** estimate even if no statistical methods were used.	Yes	Materials and Methods
Were any steps taken to minimize the effects of subjective bias when allocating animals/samples to treatment (e.g. **randomization procedure**)? If yes, have they been described?	Not Applicable	
Include a statement about **blinding** even if no blinding was done.	Yes	Materials and Methods
Describe **inclusion/exclusion criteria** if samples or animals were excluded from the analysis. Were the criteria pre-established?<br><br>If sample or data points were omitted from analysis, report if this was due to attrition or intentional exclusion and provide justification.	Not Applicable	
For every figure, are **statistical tests** justified as appropriate? Do the data meet the assumptions of the tests (e.g., normal distribution)? Describe any methods used to assess it. Is there an estimate of variation within each group of data? Is the variance similar between the groups that are being statistically compared?	Yes	Figure legends, Materials and Methods

Sample definition and in-laboratory replication	Information included in the manuscript?	In which section is the information available? (Reagents and Tools Table, Materials and Methods, Figures, Data Availability Section)
In the figure legends: state number of times the experiment was **replicated** in laboratory.	Yes	Figure legends
In the figure legends: define whether data describe **technical or biological replicates**.	Yes	Figure legends, Results

Ethics

Ethics	Information included in the manuscript?	In which section is the information available? (Reagents and Tools Table, Materials and Methods, Figures, Data Availability Section)
Studies involving **human participants**: State details of **authority granting ethics approval** (IRB or equivalent committee(s), provide reference number for approval.	Yes	Materials and Methods
Studies involving **human participants**: Include a statement confirming that **informed consent** was obtained from all subjects and that the experiments conformed to the principles set out in the WMA Declaration of Helsinki and the Department of Health and Human Services Belmont Report.	Yes	Materials and Methods
Studies involving **human participants:** For publication of **patient photos**, include a statement confirming that consent to publish was obtained.	Not Applicable	
Studies involving experimental **animals**: State details of **authority granting ethics approva**l (IRB or equivalent committee(s), provide reference number for approval. Include a statement of compliance with ethical regulations.	Not Applicable	
Studies involving **specimen and field samples:** State if relevant **permits** obtained, provide details of authority approving study; if none were required, explain why.	Not Applicable	

Dual Use Research of Concern (DURC)	Information included in the manuscript?	In which section is the information available? (Reagents and Tools Table, Materials and Methods, Figures, Data Availability Section)
Could your study fall under dual use research restrictions? Please check biosecurity documents and list of **select agents and toxins** (CDC): https://www.selectagents.gov/sat/list.htm	Not Applicable	
If you used a select agent, is the security level of the lab appropriate and reported in the manuscript?	Not Applicable	
If a study is subject to dual use research of concern regulations, is the name of the **authority granting approval and reference number** for the regulatory approval provided in the manuscript?	Not Applicable	

Reporting

The MDAR framework recommends adoption of discipline-specific guidelines, established and endorsed through community initiatives. Journals have their own policy about requiring specific guidelines and recommendations to complement MDAR.

Adherence to community standards	Information included in the manuscript?	In which section is the information available? (Reagents and Tools Table, Materials and Methods, Figures, Data Availability Section)
State if relevant guidelines or checklists (e.g., **ICMJE, MIBBI, ARRIVE, PRISMA**) have been followed or provided.	Not Applicable	
For **tumor marker prognostic studies,** we recommend that you follow the **REMARK** reporting guidelines (see link list at top right). See author guidelines, under 'Reporting Guidelines'. Please confirm you have followed these guidelines.	Not Applicable	
For **phase II and III randomized controlled trials**, please refer to the **CONSORT** flow diagram (see link list at top right) and submit the CONSORT checklist (see link list at top right) with your submission. See author guidelines, under 'Reporting Guidelines'. Please confirm you have submitted this list.	Not Applicable	

Data Availability

Data availability	Information included in the manuscript?	In which section is the information available? (Reagents and Tools Table, Materials and Methods, Figures, Data Availability Section)
Have **primary datasets** been deposited according to the journal's guidelines (see 'Data Deposition' section) and the respective accession numbers provided in the Data Availability Section?	Yes	Data Availabiliy Section
Were **human clinical and genomic datasets** deposited in a public access-controlled repository in accordance to ethical obligations to the patients and to the applicable consent agreement?	Not Applicable	
Are **computational models** that are central and integral to a study available without restrictions in a machine-readable form? Were the relevant accession numbers or links provided?	Not Applicable	
If publicly available data were reused, provide the respective **data citations in the reference list**.	Not Applicable	
